# NSP7 Molecular Degrader Attenuates Coronaviral Infection Through the β‐TrCP1/FBXO5 Axis

**DOI:** 10.1002/advs.202500798

**Published:** 2025-06-27

**Authors:** Yao Tong, Travis B. Lear, Ferhan Tuncer, John J. Villandre, Áine N. Boudreau, Bo Lin, Irene Alfaras, Jason R. Kennerdell, Daniel P. Camarco, Mads B. Larsen, Yun Hua, Yanwen Chen, Meigin E. Chandler, Ricardo Pineda, Simon M. Barratt‐Boyes, John W. Evankovich, Toren Finkel, Yuan Liu, Bill B. Chen

**Affiliations:** ^1^ Aging Institute University of Pittsburgh/UPMC Pittsburgh PA 15219 USA; ^2^ Department of Medicine Division of Pulmonary Allergy and Critical Care Medicine Acute Lung Injury Center of Excellence University of Pittsburgh Pittsburgh PA 15213 USA; ^3^ Department of Infectious Diseases and Microbiology School of Public Health University of Pittsburgh Pittsburgh PA 15213 USA; ^4^ Department of Medicine Division of Cardiology University of Pittsburgh Pittsburgh PA 15213 USA

**Keywords:** antiviral therapies, β‐TrCP1, FBXO5, non‐structural protein 7, TAF1, ubiquitination

## Abstract

NSP7 (Non‐Structural Protein 7) of SARS‐CoV‐2 is a crucial component for viral replication and transcription. In this study, it is revealed that the host E3 ubiquitin ligase FBXO5 suppresses viral replication by mediating NSP7 lysine‐48‐linked ubiquitination and subsequent proteasomal degradation. Interestingly, it is also determined that NSP7 expression impairs the host antiviral response by inhibiting the ISGylation of melanoma differentiation‐associated protein 5 (MDA5), a key sensor for viral RNA. Through an unbiased esiRNA screen, it is identified that NSP7 ubiquitination is coregulated by β‐TrCP1 and the kinase TAF1. Additionally, this study uncovers a small molecule FBXO5 stabilizer that disrupts the β‐TrCP1–FBXO5 interaction, thereby markedly enhancing NSP7 degradation and effectively mitigating SARS‐CoV‐2 infection. Taken together, the findings reveal a novel mechanism for NSP7 regulation and suggest that small‐molecule activators of the E3 ubiquitin ligase FBXO5 represent a promising new class of host‐directed antiviral therapies.

## Graphical abstract

Host E3 ligase FBXO5 as a key regulator that promotes K48‐linked ubiquitination and proteasomal degradation of SARS‐CoV‐2 NSP7, thereby suppressing viral replication. NSP7 ubiquitination is co‐regulated by β‐TrCP1 and TAF1. Small molecule BC24877 disrupts β‐TrCP1‐mediated degradation of FBXO5, stabilizing FBXO5 and enhancing NSP7 clearance, highlighting a promising host‐directed antiviral approach.

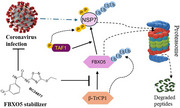

## 1 Introduction

SARS‐CoV‐2 is a novel coronavirus responsible for the COVID‐19 pandemic, characterized by its high transmissibility, ability to cause severe respiratory illness, and capacity for immune evasion and rapid mutation.^[^ ^1^, ^2^ ^]^ NSP7 is one of the most conserved non‐structural proteins (NSPs) among SARS‐related coronaviruses.^[^ ^3^ ^]^ It is a vital component of the SARS‐CoV‐2 replication and transcription machinery, forming a heterodimer with NSP8 to maintain the function of RNA‐dependent RNA polymerase (NSP12).^[^ ^4^, ^5^ ^]^ This trimeric NSP12/NSP7/NSP8 complex plays a pivotal role in viral RNA synthesis, facilitating efficient replication of the viral genome.^[^ ^6^ ^]^ The essential role of NSP7 in viral replication and its involvement in immune evasion make it a potential target for therapeutic intervention. A comprehensive understanding of the mechanisms by which NSP7 functions is essential for the development of effective strategies aimed at inhibiting SARS‐CoV‐2 replication.

Ubiquitination is a key regulatory mechanism affecting the stability and function of SARS‐CoV‐2 viral proteins, thereby modulating viral replication and pathogenicity. Multiple E3 ligases, including RNF5 and CRL4B complexes, promote the ubiquitin‐dependent degradation of ORF9b, ORF6, and the envelope protein, effectively limiting viral replication.^[^ ^7^, ^8^, ^9^, ^10^ ^]^ Conversely, deubiquitinases, including USP29 and USP1, counteract these effects by stabilizing viral proteins, enhancing their function and supporting viral persistence.^[^ ^11^, ^12^ ^]^ Notably, USP33 inhibition has been shown to promote ubiquitin‐mediated degradation of the SARS‐CoV‐2 envelope (E) protein, thereby reducing viral replication.^[^ ^13^ ^]^ These findings collectively highlight the complex interplay between ubiquitination machinery and SARS‐CoV‐2 viral proteins, presenting potential therapeutic targets for antiviral intervention.

F‐box proteins are part of the SCF (Skp1‐Cullin‐F‐box) ubiquitin ligase complex, where they function as substrate recognition components, targeting specific proteins for ubiquitin‐mediated degradation.^[^ ^14^, ^15^, ^16^ ^]^ FBXO5, also known as Emi1 (early mitotic inhibitor 1), is a member of the F‐box protein family that plays a critical role in regulating cell cycle progression by inhibiting the anaphase‐promoting complex/cyclosome (APC/C), a key ubiquitin ligase responsible for initiating anaphase and regulating mitotic exit.^[^ ^17^, ^18^ ^]^ FBXO5 itself is regulated by β‐TrCP1, another F‐box protein, which targets phosphorylated FBXO5 for ubiquitin‐mediated degradation.^[^ ^19^, ^20^ ^]^ F‐box proteins have emerged as important players in the interaction between viruses and host cells. These proteins act as substrate receptors, regulating protein degradation via the ubiquitin‐proteasome system, a mechanism often exploited by viruses to boost replication and evade immune defense.^[^ ^21^, ^22^, ^23^ ^]^ The viral inducer of RIPK3 degradation (vIRD), found in orthopoxviruses, inhibits necroptosis by promoting RIPK3 degradation via the SCF machinery, enhancing viral replication and pathogenesis.^[^ ^24^ ^]^ Human immunodeficiency virus type 1 (HIV‐1) hijacks the F‐box protein β‐TrCP1 to promote the degradation of host proteins, thereby undermining antiviral responses.^[^ ^25^, ^26^ ^]^ Similarly, FBXO2 binds to high‐mannose N‐glycans on Epstein‐Barr virus (EBV) glycoprotein B, promoting its degradation via the ubiquitin‐proteasome pathway, thereby inhibiting viral entry.^[^ ^27^ ^]^ Additionally, Hepatitis C virus (HCV) RNA requires geranylgeranylated FBXL2, which binds nonstructural protein 5A (NS5A) and forms a complex with IP3R3 to promote IP3R3 degradation, preventing apoptosis and enabling chronic infection.^[^ ^28^, ^29^ ^]^ These examples suggest that F‐box proteins can act as potential antiviral targets. In addition, we have shown previously that drugging a combinatorial F‐box protein pathway is highly effective in controlling H1N1 infection and tissue inflammation.^[^ ^30^, ^31^ ^]^

Here, we have elucidated a novel mechanism through which NSP7 inhibits antiviral immune responses and demonstrated that the host ubiquitin ligases β‐TrCP1/FBXO5 and kinase TAF1 coordinately target NSP7 for ubiquitination and degradation via the ubiquitin‐proteasome system. In addition, we identified a small molecule activator of FBXO5 that significantly enhances the degradation of NSP7 and thereby inhibits the replication of SARS‐CoV‐2.

## 2 Results

### 2.1 SARS‐CoV‐2 NSP7 is a Potential Good Therapeutic Target for Anti‐COVID‐19 Therapy

**Figure 1:**
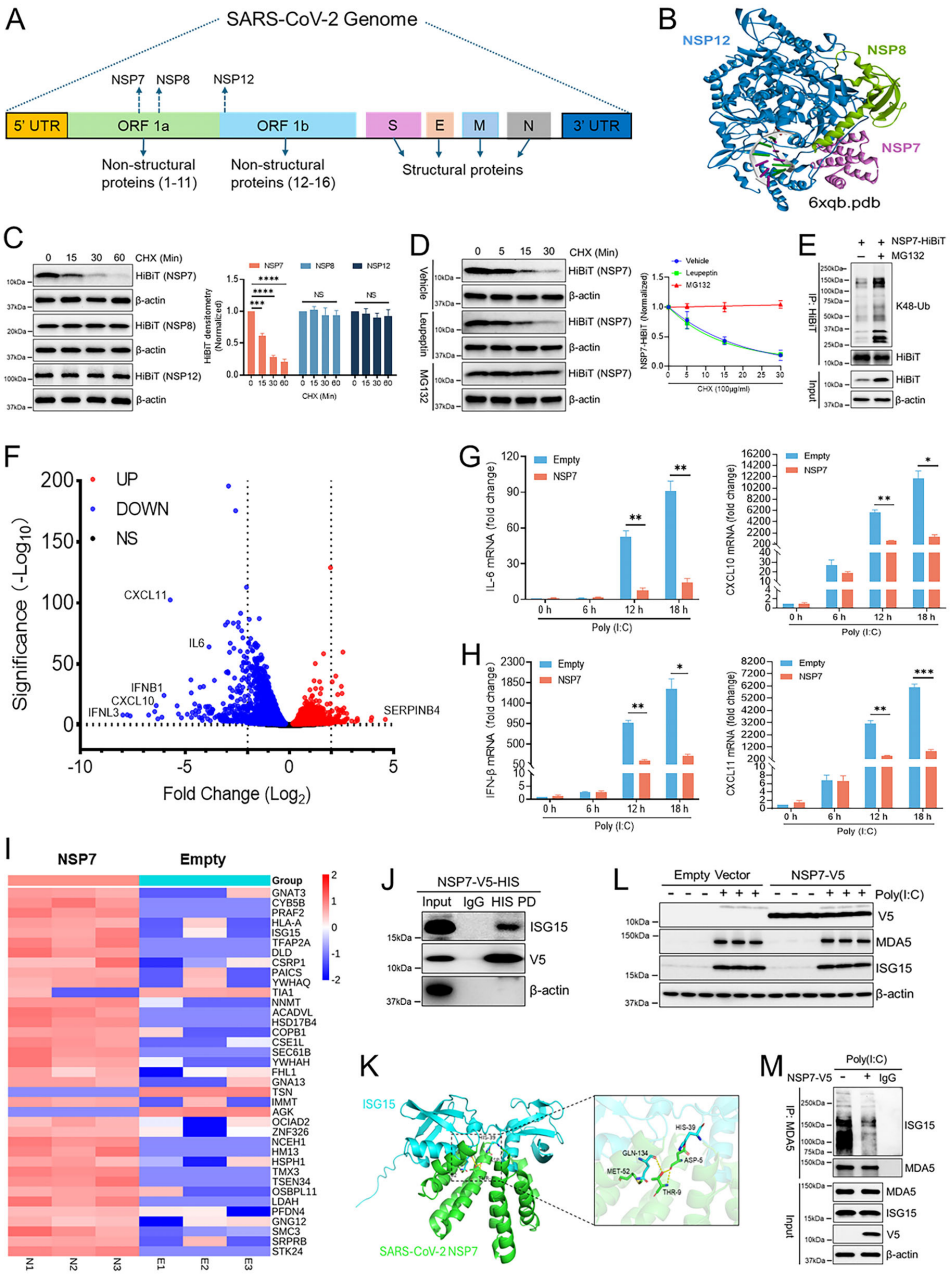
NSP7 protein undergoes proteasomal degradation and suppresses antiviral immune responses. (A) Schematic diagram of SARS‐CoV‐2 virus genome. (B) Protein structures of SARS‐CoV‐2 NSP7, NSP8 and NSP12. (C) Immunoblot analysis of lysate from BEAS‐2B cells expressing NSP7/8/12 and treated with cycloheximide (CHX, 100 µg mL^−1^), data represent mean ± SEM (*n* = 3). (D) NSP7 protein half‐life study with MG132 (10 µm) or leupeptin (10 µm) treatment, data represent mean ± SEM (*n* = 3). (E) A cell‐based ubiquitination assay was performed in BEAS‐2B cells transfected with HiBiT‐tagged NSP7 for 48 h, followed by 4 h of DMSO or MG132 treatment. NSP7 was immunoprecipitated using an anti‐HiBiT antibody, and K48‐linked ubiquitination was detected by immunoblotting with an anti‐K48 ubiquitin antibody. (F) A volcano plot from RNA‐Seq analysis illustrating differentially expressed genes (DEGs) in BEAS‐2B cells transfected with empty or NSP7 plasmid for 2 days, followed by overnight treatment with Poly (I:C) (5 ng mL^−1^, transfected using Lipofectamine 3000). (G,H) The expression of selected DEGs, including *IL‐6*, *IFN‐β*, *CXCL10*, and *CXCL11*, was assessed by qPCR in BEAS‐2B cells transfected with or without NSP7 for 2 days, followed by overnight treatment with Poly (I:C), data represent mean ± SEM (*n* = 3). (I) Heatmap of differentially expressed proteins identified by V5 IP‐mass spectrometry in BEAS‐2B cells transfected with NSP7‐V5 versus empty‐V5 vector controls. (J) A binding assay was performed to assess the interaction between ISG15 and NSP7. NSP7‐HIS was pulled down using HIS‐tag magnetic beads, and the presence of bound ISG15 was detected by immunoblotting. (K) Molecular docking analysis illustrates ISG15 and NSP7 interactions. (L) Immunoblot analysis of MDA5 and ISG15 levels in BEAS‐2B cells transfected with or without NSP7, followed by overnight treatment with Poly (I:C). (M) Endogenous MDA5 ISGylation was assessed by immunoprecipitation (IP) using anti‐MDA5 (or an IgG isotype control) in BEAS‐2B cells transfected with either NSP7 or an empty vector, prior to treatment with Poly (I:C). Following IP, the samples were probed with anti‐ISG15. *p < 0.05, **p < 0.01, ***p < 0.001, ****p < 0.0001 by one‐way ANOVA with Dunnett's multiple comparisons (C), or unpaired two‐tailed Student's *t*‐test (G,H). NS, not significant.

To aid in the development of host‐centric therapeutics for SARS‐CoV‐2 infection, we sought to analyze the cellular regulation of SARS‐CoV‐2 RNA polymerase stability, composed of a trimeric NSP12/NSP7/NSP8 complex (**Figure** **1**A,B). We first conducted a cycloheximide (CHX) chase experiment to determine the protein half‐life of NSP12/NSP7/NSP8 proteins. NSP7 exhibited a strikingly short half‐life of ≈15 min in human bronchial epithelial cells (Beas‐2B) (Figure 1C). This process is regulated by the proteasome, as the proteasome inhibitor MG132 completely rescued the decline in NSP7 levels (Figure 1D). NSP7 degradation is also mediated by K48 ubiquitination (Figure 1E).

Taking advantage of the NSP7 short half‐life, we then focused on identifying the molecular mechanism that controlled its degradation. Through RNAseq analysis, we observed that ectopic expression of NSP7 following poly(I:C) stimulation specifically down‐regulated host viral signaling pathways (Figure 1F; Figure S1A–C, Supporting Information). As shown in Figure S2 (Supporting Information), poly(I:C) treatment markedly induced antiviral gene expression in Beas‐2B cells. However, NSP7 expression suppressed poly(I:C) induced antiviral cytokines expression including IFNL3, IFNB1, CXCL10, thus significantly impacts host anti‐viral pathways. We further confirmed the RNAseq findings by repeating the study and measured cytokine gene expression through qPCR (Figure 1G,H; Figure S3A,B, Supporting Information). In addition to qPCR analysis, we also measured the cytokine secretion by ELISA and showed that NSP7 suppresses poly(I:C) induced CXCL1, IFN, and IL‐6 secretion (Figure S3C–F, Supporting Information).

To understand the mechanism of how does NSP7 suppresses host anti‐viral response, we performed unbiased proteomics study trying to identify the potential binding partners of NSP7. NSP7‐V5 Immunoprecipitation (IP) and MS analysis detected ISG15 as one of the potential NSP7 binders from Beas‐2B cells (Figure 1I). We further confirmed this finding through NSP7 HIS‐PD (Figure 1J). Molecular docking analysis^[^ ^32^ ^]^ also provided structural hints on ISG15 and NSP7 interactions (Figure 1K), leading to the identification of the T9A mutation on NSP7 through pull‐down assays, which significantly weakened its binding affinity to ISG15 (Figure S4A, Supporting Information). ISG15 plays an essential role in initiating antiviral responses by promoting the ISGylation of various cellular proteins, including essential sensors like melanoma differentiation‐associated protein 5 (MDA5). Interestingly, we observed that NSP7 expression did not alter MDA5 and ISG15 protein levels (Figure 1L). However, it strongly inhibits poly(I:C) induced MDA5 ISGylation (Figure 1M), an effect that was abolished by the T9A mutation on NSP7 (Figure S4B, Supporting Information).

### 2.2 The E3 Ligase Complex SCFFBXO5 Mediates SARS‐CoV2 NSP7 Degradation

**Figure 2:**
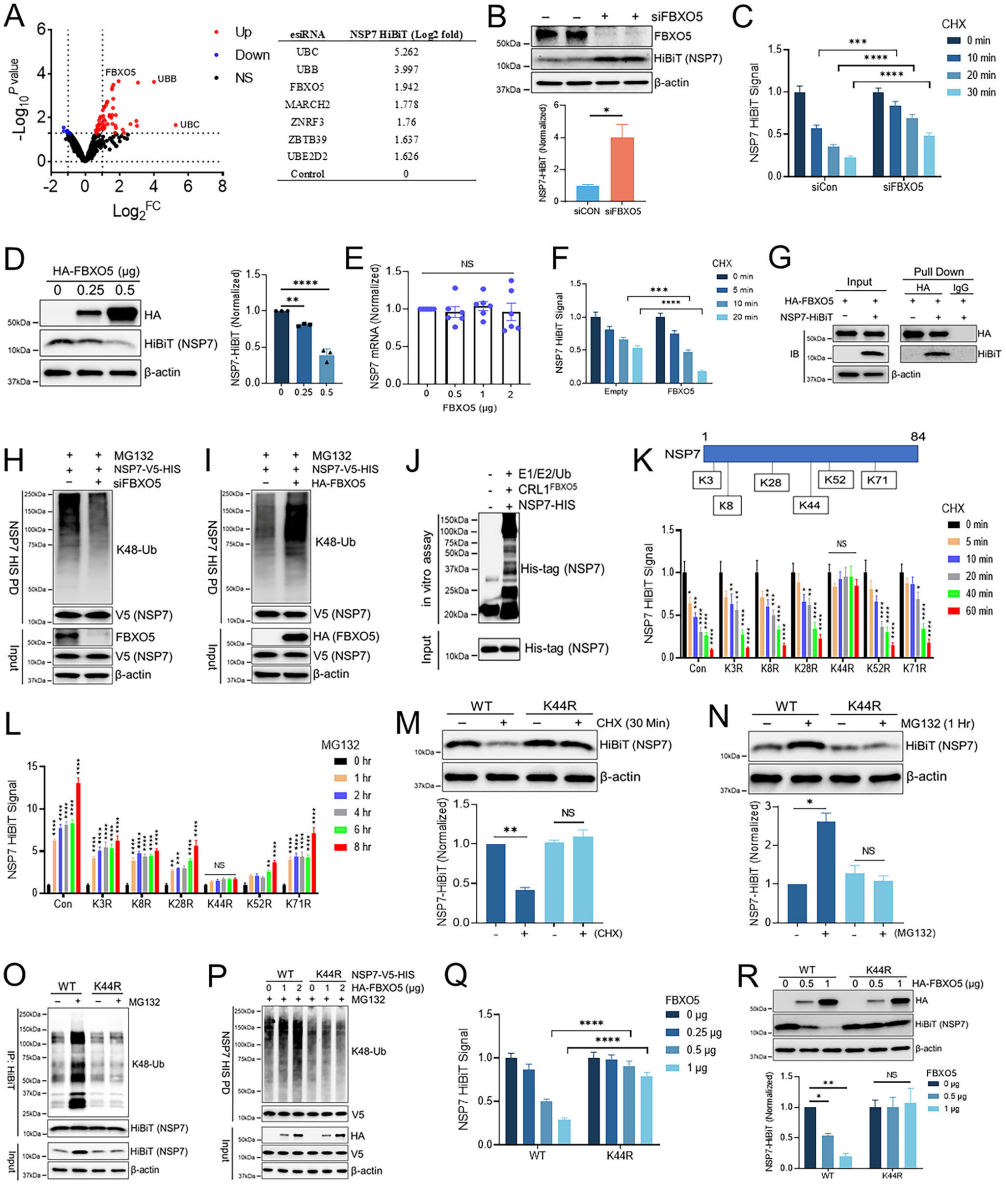
E3 ubiquitin ligase FBXO5 mediates SARS‐CoV2 NSP7 degradation. (A) Screening of CoV2‐NSP7‐HiBiT BEAS‐2B stable cell line with RNAi library targeting components of the ubiquitin‐proteasome system (≈836 targets). Top hits listed in the chart. (B) FBXO5 was knocked down for 2 days in NSP7‐HiBiT‐BEAS‐2B cells, followed by immunoblot analysis to assess FBXO5 and HiBiT protein levels, data represent mean ± SEM (*n* = 3). (C) Luminescence Nano‐Glo^®^ HiBiT assay for NSP7 protein level with FBXO5 knockdown in BEAS‐2B cells treated with CHX, data represent mean ± SEM (*n* = 16). (D) Immunoblot analysis and (E) qPCR analysis of NSP7‐HiBiT‐ BEAS‐2B cells with increasing expression of FBXO5, data represent mean ± SEM (*n* = 3). (F) Luminescence HiBiT assay of NSP7‐HiBiT‐BEAS‐2B cells with ectopic expression of FBXO5, data represent mean ± SEM (*n* = 3). (G) A binding assay was performed for NSP7‐HiBiT using HA‐tag magnetic beads to pull down HA‐FBXO5. (H) A cell‐based ubiquitination assay was performed in BEAS‐2B cells transfected with V5‐HIS‐tagged NSP7 and FBXO5 siRNA or (I) HA‐tagged FBXO5 for 48 h (h), followed by treatment with MG132 for 4 h. NSP7 was pulled down using HIS‐tag magnetic beads, and K48‐linked ubiquitination was detected by immunoblotting with an anti‐K48 ubiquitin antibody. (J) Immunoblotting of in vitro ubiquitination assay involving the full complement of ubiquitination machinery (E1, E2, ubiquitin, ATP, Mg^2+^) and SCFFBXO5 protein. (K) Schematic of NSP7 protein domains and potential lysine sites for ubiquitin conjugation. Profiling of NSP7 Lys‐Arg (K‐R) constructs and their response to CHX or (L) proteasomal inhibition, data are a ratio of CHX/MG132 treated luminescence relative to vehicle‐treated luminescence, data represent mean ± SEM (*n* = 16). (M) Immunoblotting of NSP7‐HiBiT from NSP7 wild‐type (WT)/K44R‐HiBiT BEAS‐2B cells with CHX or (N) MG132 treatment, data represent mean ± SEM (*n* = 3). (O) A cell‐based ubiquitination assay was performed in BEAS‐2B cells transfected with HiBiT‐tagged NSP7 WT or K44R mutant for 48 h, followed by treatment with MG132 for 4 h, determined by IP with anti‐HiBiT and IB with anti‐K48 ubiquitin. (P) A cell‐based ubiquitination assay was performed in BEAS‐2B cells transfected with HA‐tagged FBXO5 and V5‐HIS‐tagged NSP7 WT or K44R mutant for 48 h, followed by treatment with MG132 for 4 h. NSP7 was pulled down using HIS‐tag magnetic beads, and K48‐linked ubiquitination was detected by immunoblotting with an anti‐K48 ubiquitin antibody. (Q) Luminescence HiBiT assay and (R) Immunoblot analysis for NSP7‐HiBiT protein level in NSP7‐WT‐HiBiT and K44R‐HiBiT BEAS‐2B cells with increasing expression of FBXO5, data represent mean ± SEM (*n* = 3). *p < 0.05, **p < 0.01, ***p < 0.001, ****p < 0.0001 compared to vehicle or control or as indicated by one‐way ANOVA with Dunnett's multiple comparisons (D,K,L,R), or unpaired two‐tailed Student's *t*‐test (B,C,F,M,N,Q). NS, not significant.

To identify the E3 ligase regulating NSP7 ubiquitination, we first constructed Beas‐2B cells that stably express a fusion NSP7‐HiBiT protein. An assay for NSP7 expression was miniaturized into 384‐well plate while maintaining a robust Z‐score (Figure S5A, Supporting Information). We then used a custom siRNA library targeting proteins involved in ubiquitination (E1, E2, E3, Proteasome subunits, and DUBs, etc.) to measure the NSP7‐HiBiT signal in Beas‐2B cells. This unbiased approach uncovered FBXO5 as a key regulator for NSP7 protein abundance (**Figure** **2**A). In support of these screening results, we observed that the knockdown of FBXO5 significantly increased NSP7 total protein level and half‐life (Figure 2B,C). Conversely, the ectopic expression of HA‐FBXO5 dose‐dependently reduced NSP7 protein levels without affecting NSP7 mRNA (Figure 2D,E). Increased FBXO5 expression also reduced the half‐life of NSP7 protein (Figure 2F). Furthermore, HA‐FBXO5 pull‐down assays confirmed its interaction with NSP7 (Figure 2G). In addition, FBXO5 knockdown reduced NSP7 K48 polyubiquitination, whereas augmenting FBXO5 expression increased NSP7 K48 polyubiquitination (Figure 2H,I). Using recombinant proteins, we observed that a SCF‐FBXO5 complex could ubiquitinate NSP7 (Figure 2J). Together, this evidence supports that FBXO5 is the authentic E3 ligase subunit that targets NSP7 for ubiquitination. We further identified the lysine ubiquitination site within NSP7. Through mutagenesis studies, we observed that a K44R mutation of NSP7 exhibited a significantly extended half‐life in CHX chase experiment (Figure 2K,M). Since it lacks the ubiquitination site, NSP7 K44R also failed to accumulate upon treatment with a proteasome inhibitor (Figure 2L,N). In addition, the NSP7 K44R mutant was resistant to the accumulation of K48‐linked polyubiquitination following MG132 treatment or increased FBXO5 expression (Figure 2O,P). The NSP7 K44R mutant was also resistant to FBXO5‐stimulated NSP7 protein degradation (Figure 2Q,R). Finally, in functional studies (Figure S5B–I, Supporting Information), FBXO5 expression was able to counteract NSP7 suppression of antiviral signaling pathways (IL6, CXCL1, IFNβ, IFNλ mRNA, and protein). In addition, we suspect that NSP7 of other beta coronaviruses such as HCoV‐OC43 is also ubiquitinated and exhibits similar protein half‐life compared to NSP7 of SARS‐CoV‐2. Indeed, as shown in Figure S6A (Supporting Information), NSP7 of HCoV‐OC43 has a protein half‐life of ≈10 min, which is comparable to NSP7 of SARS‐CoV‐2. We have also performed the mutagenesis study and confirmed that K52 residue within NSP7 of HCoV‐OC43 is important in protein stability. As shown in Figure S6B (Supporting Information), HCoV‐OC43 NSP7 K52R exhibited a significantly extended half‐life in CHX chase experiment. Since it lacks the ubiquitination site, HCoV‐OC43 NSP7 K52R also failed to accumulate upon treatment with a proteasome inhibitor (Figure S6C, Supporting Information). This mutant was resistant to FBXO5‐stimulated NSP7 protein degradation (Figure S6D, Supporting Information). In addition, it was resistant to the accumulation of K48‐linked polyubiquitination following MG132 treatment or increased FBXO5 expression (Figure S6E, Supporting Information). These studies provided evidence that NSP7 proteins from both SARS‐CoV‐2 and HCoV‐OC43 shared similar properties and degradation pathway in cells. These studies also suggest that NSP7 degradation by FBXO5 could be a common mechanism shared by several if not all the beta‐coronaviral family members.

### 2.3 The TAF1 Primes SARS‐CoV‐2 NSP7 for SCFFBXO5 Dependent Degradation

**Figure 3:**
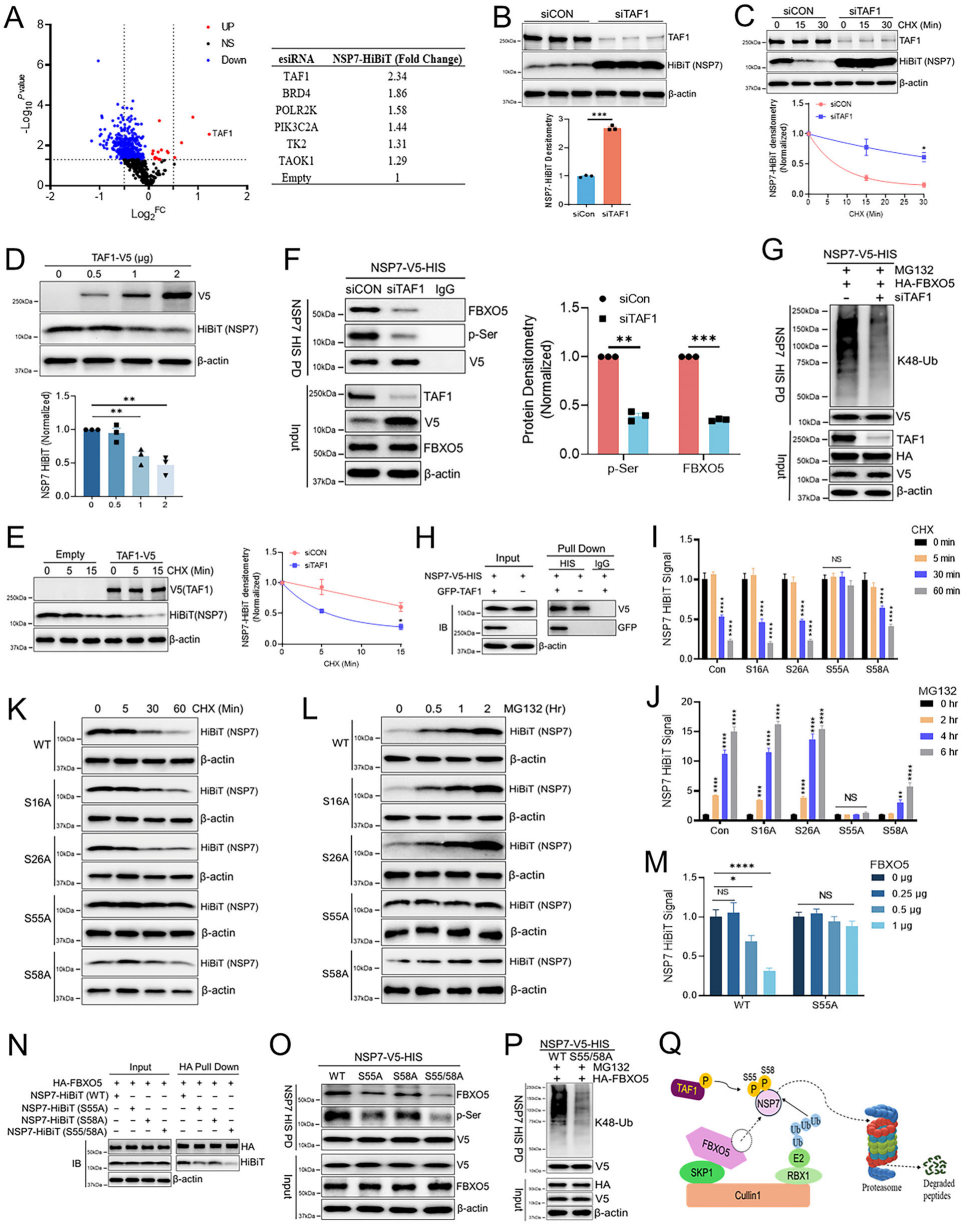
The kinase TAF1 primes SARS‐CoV2 NSP7 for FBXO5‐dependent degradation. (A) Screening of CoV2‐NSP7‐HiBiT BEAS‐2B stable cell line with RNAi library targeting components of kinases (≈638 targets). Top hits listed in the chart. (B) TAF1 siRNA was transfected in NSP7‐HiBiT‐BEAS‐2B cells for 2 days, followed by immunoblotting of FBXO5 and HiBiT protein abundance, data represent mean ± SEM (*n* = 3). (C) Immunoblot analysis for NSP7‐HiBiT protein levels with TAF1 knockdown in BEAS‐2B cells treated with CHX, data represent mean ± SEM (*n* = 3). (D) Immunoblotting of NSP7‐HiBiT protein abundance from NSP7‐HiBiT‐BEAS‐2B cells with increasing expression of TAF1, data represent mean ± SEM (*n* = 3). (E) Immunoblotting of NSP7‐HiBiT‐BEAS‐2B cells transfected with empty or TAF1 plasmid before CHX treatment, data represent mean ± SEM (*n* = 3). (F) Pull‐down assay was performed in BEAS‐2B cells transfected with V5‐HIS‐tagged NSP7 and TAF1 siRNA for 48 h. NSP7 was pulled down using HIS‐tag magnetic beads, and protein amounts were detected by immunoblotting with anti‐phospho‐serine (p‐ser), anti‐FBXO5, anti‐V5, and anti‐TAF1 antibodies. (G) A cell‐based ubiquitination assay was performed in BEAS‐2B cells transfected with V5‐HIS‐tagged NSP7, HA‐tagged FBXO5, and TAF1 siRNA for 48 h, followed by treatment with MG132 for 4 h. NSP7 was pulled down using HIS‐tag magnetic beads, and K48‐linked ubiquitination was detected by immunoblotting with an anti‐K48 ubiquitin antibody. (H) Binding assay of TAF1, with pulled‐down NSP7‐HIS. (I) Profiling of NSP7 Ser‐Ala (S‐A) constructs and their response to CHX or (J) proteasomal inhibition, data are a ratio of CHX/MG132 treated luminescence relative to vehicle‐treated luminescence, data represent mean ± SEM (*n* = 16). (K) Immunoblotting of HiBiT protein amount from NSP7‐WT‐HiBiT and NSP7 serine mutant‐HiBiT cells treated with CHX or (L) MG132. (M) Luminescence HiBiT assay for NSP7 protein level in NSP7‐WT‐HiBiT and S55A‐HiBiT BEAS‐2B cells with increasing expression of FBXO5, data represent mean ± SEM (*n* = 8). (N) A Binding assay in BEAS‐2B cells transfected with HA‐tagged FBXO5 and HiBiT‐tagged NSP7 WT or mutants for 48 h, determined by HA pull‐down (PD) and IB with anti‐HiBiT. (O) HIS PD assay for phospho‐serine (p‐ser) and FBXO5 protein levels in NSP7‐WT‐HiBiT and NSP7‐serine mutant‐HiBiT BEAS‐2B cells. A Binding assay in BEAS‐2B cells transfected with HA‐tagged FBXO5 and HiBiT‐tagged NSP7 WT or mutants for 48 h, determined by HA PD and IB with anti‐HiBiT. (P) Cell‐based ubiquitination assay of NSP7‐V5‐HIS in BEAS‐2B cells with ectopic FBXO5 expression. (Q) Schematic of TAF1‐induced priming phosphorylation required for FBXO5‐mediated NSP7 poly‐ubiquitination. *p < 0.05, **p < 0.01, ***p < 0.001, ****p < 0.0001 compared to vehicle or control or as indicated by one‐way ANOVA with Dunnett's multiple comparisons (D,I,J,M), or unpaired two‐tailed Student's *t*‐test (B,C,E,F). NS, not significant.

The Cullin‐based E3 ligase complex typically targets phosphorylated substrates,^[^ ^14^, ^30^, ^33^ ^]^ thus we examined whether a specific kinase could lead to NSP7 phosphorylation, thereby priming NSP7 for ubiquitin‐proteasomal degradation. Using the same NSP7‐HiBiT stable cell line and a kinase esiRNA library consisting of all 600 kinases (**Figure** **3**A), we uncovered transcription initiation factor TFIID subunit 1 (TAF1) as the potential kinase for NSP7 phosphorylation. TAF1 knockdown significantly increased NSP7 total protein level and extended its half‐life (Figure 3B,C). Conversely, the ectopic expression of TAF1‐V5 reduced NSP7 protein level and protein half‐life (Figure 3D,E). TAF1 knockdown also significantly reduced FBXO5/NSP7 interaction and NSP7 serine phosphorylation and polyubiquitination (Figure 3F,G). We also showed through pull‐down analysis that TAF1 interacts with NSP7 (Figure 3H). Through mutagenesis studies, we observed that an S55A mutation of NSP7 completely blocked CHX‐induced protein decay (Figure 3I,K). NSP7 S55A also did not accumulate upon proteasome inhibitor MG132 treatment (Figure 3J,L). In addition, the mutation of a nearby serine, NSP7 S58A, also exhibited partial effects in these assays. NSP7 S55A was resistant to FBXO5 expression‐induced NSP7 protein degradation (Figure 3M). Finally, through pull‐down (PD) assays (HA‐FBXO5 PD, NSP7‐HIS PD), we have shown that the S55A/S58A double mutation leads to marked reduction of NSP7/FBXO5 interaction (Figure 3N,O), NSP7 serine phosphorylation (Figure 3O) and NSP7 polyubiquitination (Figure 3P). Together, this suggests TAF1‐mediated NSP7 phosphorylation generates a critical molecular motif for substrate engagement by the FBXO5‐E3 ligase complex for subsequent ubiquitination (Figure 3Q).

To further confirm this model, we deployed a pharmacological approach taking advantage of the available TAF1 inhibitor (BAY‐299). As shown in Figure S7A,B (Supporting Information), BAY‐299 dose‐dependently and time‐dependently increased NSP7‐HiBiT signal in cells. BAY‐299 significantly extended NSP7 protein half‐life (Figure S7C, Supporting Information). Finally, through three binding assays (NSP7 HiBiT IP, HA‐FBXO5 PD, NSP7‐HIS PD), we have shown that TAF1 inhibitor leads to drastic reduction of NSP7/FBXO5 interaction (Figure S7D,E, Supporting Information), NSP7 serine phosphorylation (Figure S7D, Supporting Information) and NSP7 polyubiquitination (Figure S7F, Supporting Information). These TAF1 inhibitor effects mimic what was observed using TAF1 siRNA. Thus, through both genetic and pharmacological approaches, we have illustrated the role of TAF1 as a critical regulator for FBXO5‐mediated NSP7 ubiquitination.

### 2.4 β‐TrCP1/FBXO5 Axis Co‐Regulates SARS‐CoV‐2 NSP7 for Ubiquitin Dependent Degradation

**Figure 4:**
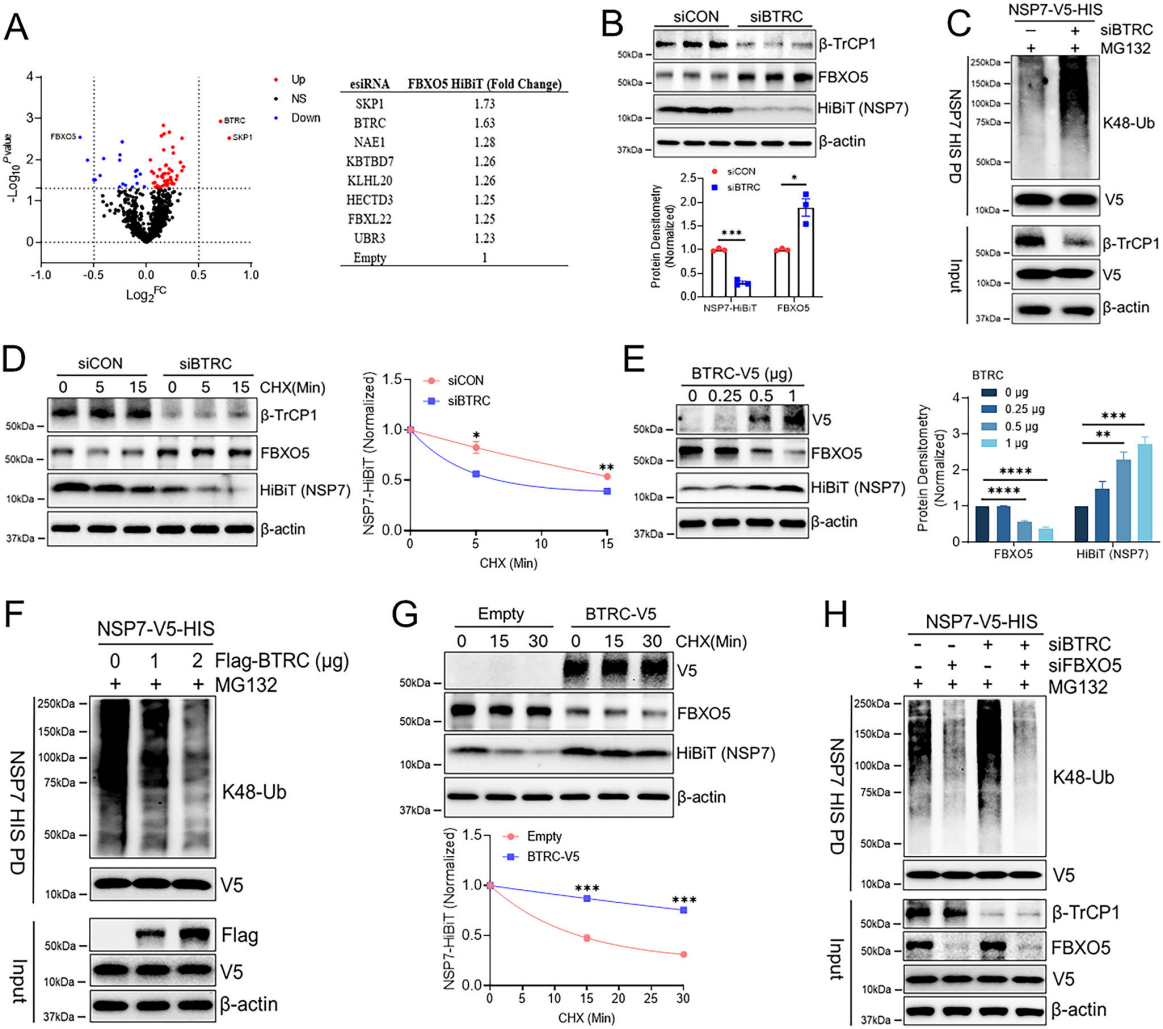
The β‐TrCP1/FBXO5 axis co‐regulates the ubiquitin‐dependent degradation of SARS‐CoV‐2 NSP7. (A) Screening of BEAS‐2B FBXO5‐HiBiT CRISPR knock‐in cell line with RNAi library targeting components of the ubiquitin‐proteasome system (≈836 targets). Top hits were listed in the chart. (B) Immunoblot analysis of FBXO5 and NSP7 protein amounts from NSP7‐HiBiT‐BEAS‐2B cells with *β‐TrCP1* gene (BTRC) knockdown, data represent mean ± SEM (*n* = 3). (C) A cell‐based ubiquitination assay was performed in BEAS‐2B cells transfected with V5‐HIS‐tagged NSP7 and β‐TrCP1 siRNA for 48 h, followed by treatment with MG132 for 4 h. NSP7 was pulled down using HIS‐tag magnetic beads, and K48‐linked ubiquitination was detected by immunoblotting with an anti‐K48 ubiquitin antibody. (D) Immunoblot analysis of FBXO5 and HiBiT protein abundance with β‐TrCP1 knockdown in NSP7‐HiBiT‐BEAS‐2B cells treated with CHX, data represent mean ± SEM (*n* = 3). (E) Immunoblotting of FBXO5 and HiBiT protein amounts from NSP7‐HiBiT‐BEAS‐2B cells with increasing expression of β‐TrCP1, data represent mean ± SEM (*n* = 3). (F) Cell‐based ubiquitination assay of NSP7‐V5‐HIS in BEAS‐2B cells with increasing expression of β‐TrCP1. (G) Immunoblotting of NSP7‐HiBiT‐BEAS‐2B cells transfected with empty or β‐TrCP1 plasmid before CHX treatment, data represent mean ± SEM (*n* = 3). (H) Cell‐based ubiquitination assay of NSP7‐V5‐HIS in BEAS‐2B cells with β‐TrCP1 and/or FBXO5 knockdown. *p < 0.05, **p < 0.01, ***p < 0.001, ****p < 0.0001 by one‐way ANOVA with Dunnett's multiple comparisons (E), or unpaired two‐tailed Student's *t*‐test (B,D,G).

FBXO5 has been shown to have a short half‐life in several previous studies.^[^ ^19^, ^20^, ^34^, ^35^ ^]^ Evidence suggests that the stability of FBXO5 is regulated by β‐TrCP1, another F‐box protein. To confirm this and explore other potential E3 ligase that target FBXO5, we first engineered Beas‐2B cells that express endogenously tagged FBXO5 protein (FBXO5‐HiBiT) using CRISPR technology. This provided a sensitive and quantitative readout of endogenous FBXO5 protein. An assay for FBXO5 expression was then miniaturized into a 384‐well plate and demonstrated to have a good Z‐score (Figure S8A, Supporting Information). Using the same esiRNA library described in Figure 2A, we identified β‐TrCP1 (BTRC) and SKP1 as the top regulators involved in FBXO5 protein stability (**Figure** **4**A). SKP1 is the adaptor molecule for the SCF complex. Knockdown of β‐TrCP1 significantly increased FBXO5 and reduced NSP7 protein level (Figure 4B). β‐TrCP1 knockdown also drastically increased NSP7 polyubiquitin and reduced its protein half‐life (Figure 4C,D). Conversely, β‐TrCP1 overexpression reduced FBXO5 and increased NSP7 protein levels (Figure 4E). β‐TrCP1 expression also drastically reduced NSP7 polyubiquitin and extended its protein half‐life (Figure 4F,G). The negative correlation between β‐TrCP1 and FBXO5 can also be observed in SARS‐CoV‐2 infected cells in a time‐dependent manner (Figure S8B, Supporting Information). Finally, we also deployed a pharmacological approach using a known β‐TrCP1 inhibitor (GS143). GS143 dose‐dependently and time‐dependently reduced NSP7 protein and its protein half‐life (Figure S8C–E, Supporting Information). GS143 also increased NSP7 K48 polyubiquitination (Figure S8F, Supporting Information). Finally, to confirm the role of β‐TrCP1/FBXO5 axis in regulating NSP7 ubiquitination, we performed a double knockdown experiment using both β‐TrCP1 and FBXO5 siRNAs. As shown in Figure 4H, FBXO5 knockdown nearly completely abrogates NSP7 K48 ubiquitination irrespective of β‐TrCP1 protein level in cells. This study highlights the importance of maintaining FBXO5 protein level to degrade NSP7.

### 2.5 Developing a Novel FBXO5 Stabilizer

**Figure 5:**
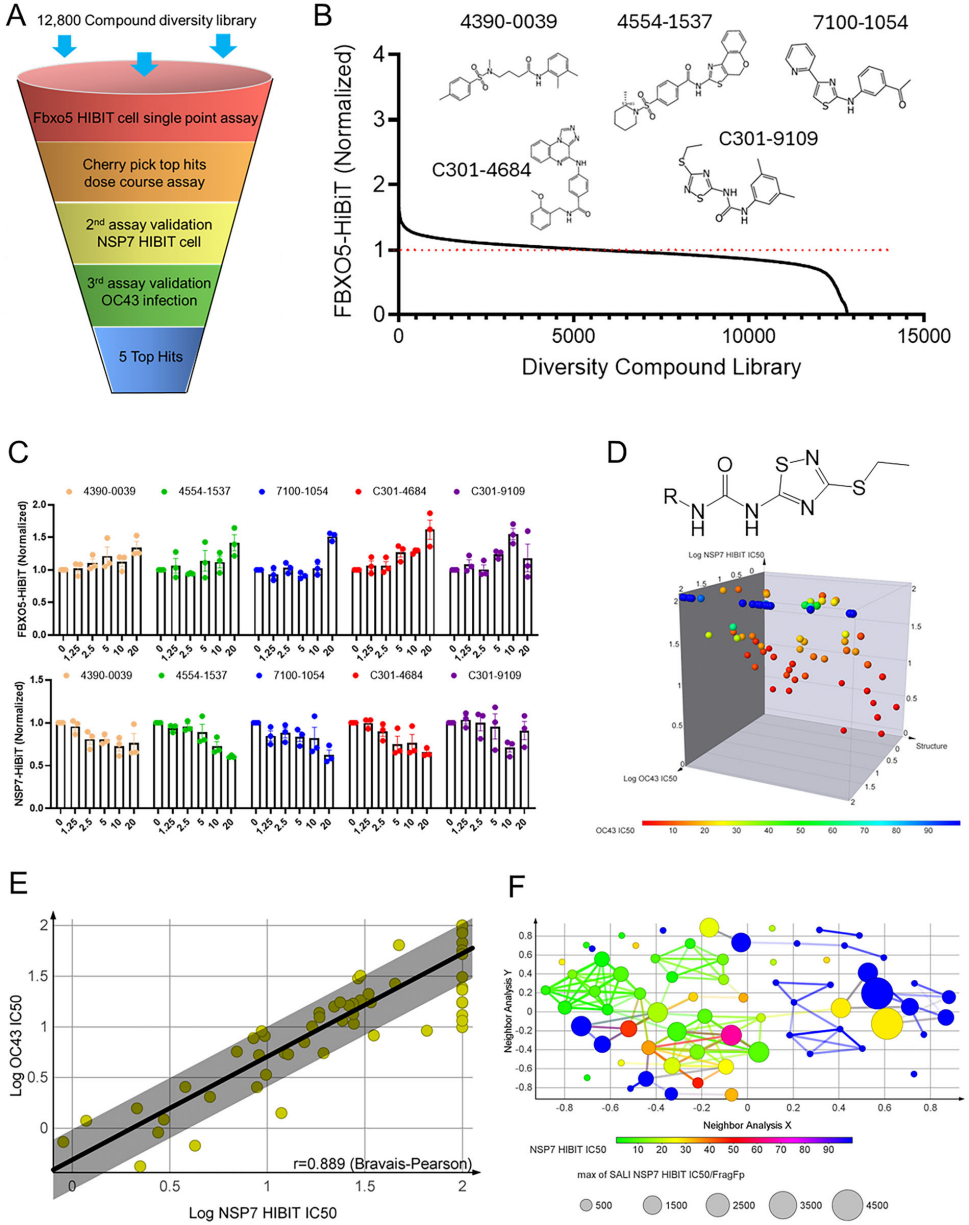
Development of novel FBXO5 stabilizer. (A) Schematic representation of the compound screening process. (B) Screening of the BEAS‐2B FBXO5‐HiBiT CRISPR knock‐in cell line with Diversity Compound Library (≈12 800 compounds) targeting FBXO5. Top hits were listed in the chart. (C) Luminescence Nano‐Glo® HiBiT assay to measure FBXO5 and NSP7 protein levels following treatment with top hit compounds, data represent mean ± SEM (*n* = 16). (D,E) Markush structure of FBXO5 activators and their structure‐activity data, plotted with log IC50 of compound activity in reducing human coronavirus (HCoV‐OC43) infection and NSP7‐HiBiT signal. (F) Structure‐Activity Landscape Index (SALI) analysis of the FBXO5 activators. SALI provides a simple means of identifying pairs of compounds where a small change in chemical structure brings about a large change in biological activity or physical properties.

Given that β‐TrCP1 inhibitor GS143 degrades SARS‐CoV‐2 NSP7 protein, it could in theory be effective as a new therapeutic for COVID‐19. However, β‐TrCP1 has diverse sets of downstream substrates, therefore a direct inhibitor of β‐TrCP1 would likely have off‐targeting effect (Figure S8G, Supporting Information). Thus, we thought to identify additional compounds that could increase FBXO5 through different mechanisms. Using the same Beas‐2B cells that express endogenous FBXO5‐HiBiT protein from Figure 4, we first screened an in‐house diversity library of 12 800 compounds to identify compounds that increase FBXO5 HiBiT protein (**Figure** **5**A,B). The top hits were then selected and tested in a dose course in Beas‐2B cells that stably express NSP7 HiBiT protein (Second assay). Finally, some of the molecules were further selected to test in HCoV‐OC43 infection assay (First assay). OC43 is a human beta coronavirus strain associated with the common cold. It has been routinely used to evaluate compounds with potential anti‐COVID‐19 efficacy.

We used the screening algorithm delineated above to identify the five best molecules (Figure 5A,B). These molecules were each able to dose‐dependently increase FBXO5 HiBiT protein but decrease NSP7 HiBiT protein (Figure 5C). Cellular ATP levels were also evaluated to determine the compound toxicity (Figure S9, Supporting Information). In considering the chemical scalability and structural diversity, we selected C301‐9109 as our hit molecule and initiated a hit‐to‐lead campaign. C301‐9109 has a 1,2,4‐thiadiazol core, a urea linker, an ethylsulfanyl side chain and a 3,5‐dimethylphenyl ring. Our focus was to modify or replace the dimethylphenyl ring (The R group shown in Figure 5D). We developed a strategy to synthesize a library of similar SAR molecules (see Supporting Information). The synthetic route has three steps involved using starting material thiourea. Once the common intermediate 3‐(ethylthio)‐1,2,4‐thiadiazol‐5‐amine was obtained, we then deployed a combinatorial approach using various corresponding isocyanates to synthesize the final SAR compounds. As shown in Figure 5D and Table S1 (Supporting Information), ≈60 novel compounds were synthesized and tested in the Structure Activity Relationship (SAR) studies that included the NSP7 HiBiT assay and OC43 infection assay. Compound IC50s were plotted with their structural information in a 3D chart. Most of the SAR compounds showed consistent activity between both assays (Figure 5E), suggesting compounds are likely on target. Finally, a Structure‐Activity Landscape Index (SALI) plot was generated based upon the compound activity data to further summarize the SAR (Figure 5F). SALI plot is a visualization technique for identifying and quantifying activity cliffs in a SAR dataset. From these studies, we identified 3‐CF3 group on the phenyl ring offers the best activity boost and its corresponding compound BC24877 was selected to test further in the various functional assays.

### 2.6 Novel NSP7 Degrader Attenuates SARS‐CoV‐2 Infection Through the β‐TrCP1/FBXO5 Axis

**Figure 6:**
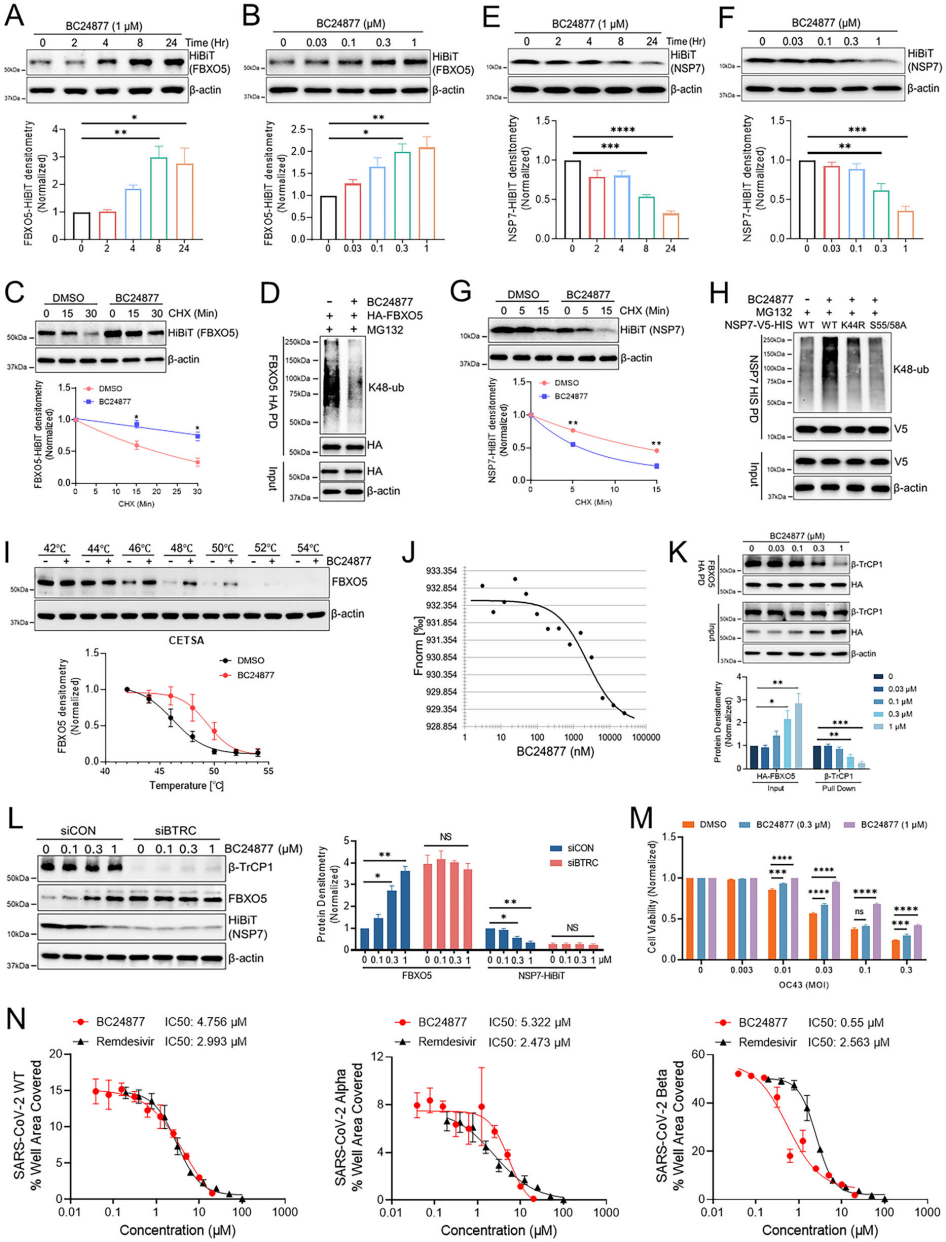
A novel NSP7 degrader attenuates SARS‐CoV infection via the β‐TrCP1/FBXO5 axis. (A) Immunoblotting of HiBiT protein abundance from FBXO5‐HiBiT‐BEAS‐2B cells treated with BC24877 (1 µm) in a time‐dependent or (B) dose‐dependent (24 h) manner, data represent mean ± SEM (*n* = 3). (C) Immunoblot analysis of HiBiT protein abundance from FBXO5‐HiBiT‐BEAS‐2B cells exposed to BC24877 (1 µm) for 24 h, followed by CHX treatment, data represent mean ± SEM (*n* = 3). (D) Cell‐based ubiquitination assay of HA‐FBXO5 in BEAS‐2B cells with vehicle or BC24877 (1 µm) treatment. FBXO5 was pulled down prior to washing and immunoblotting. (E) Immunoblotting of HiBiT protein abundance from SARS‐CoV2‐NSP7‐HiBiT‐BEAS‐2B cells treated with BC24877 (1 µm) in a time‐dependent or (F) dose‐dependent (24 h) manner, data represent mean ± SEM (*n* = 3). (G) Immunoblot analysis of HiBiT protein abundance from NSP7‐HiBiT‐BEAS‐2B cells exposed to BC24877 (1 µm) for 24 h, followed by CHX treatment, data represent mean ± SEM (*n* = 3). (H) A cell‐based ubiquitination assay was performed in BEAS‐2B cells expressing V5‐His‐tagged NSP7 wild‐type (WT) or lysine/serine mutant. Cells were treated with vehicle or BC24877 (1 µm) for 24 h, followed by MG132 treatment for 4 h. NSP7 was pulled down using HIS‐tag magnetic beads, and K48‐linked ubiquitination was detected by immunoblotting with an anti‐K48 ubiquitin antibody. (I) CETSA analysis of FBXO5 protein in BEAS‐2B cells treated with vehicle or BC24877 (1 µm), data represent mean ± SEM (*n* = 3). (J) Microscale thermophoresis (MST) of FBXO5 and BC24877. Fluorescently labeled FBXO5 protein was incubated with titration of compound concentrations, heated across a temperature gradient and thermophoretic fluorescent change measured. Normalized fluorescence of heated vs unheated (Fnorm) was determined and plotted against BC24877 concentration to calculate a binding coefficient. (K) A binding assay was performed in BEAS‐2B cells treated with BC24877 for 24 h to assess the interaction between β‐TrCP1 and HA‐FBXO5. HA‐FBXO5 was pulled down using HA‐tag magnetic beads, and the presence of β‐TrCP1 in the precipitates was detected by immunoblotting, data represent mean ± SEM (*n* = 3). (L) Immunoblotting of FBXO5 and HiBiT protein amounts from SARS‐CoV2‐NSP7‐HiBiT‐BEAS‐2B cells with BTRC knockdown prior to BC24877 treatment, data represent mean ± SEM (*n* = 3). (M) Cell viability assay in BEAS‐2B cells treated with BC24877 and simultaneously infected with HCoV‐OC43 for 3 days, data represent mean ± SEM (*n* = 16). (N) IC50 analysis of BC24877 and Remdesivir from Vero cells infected with SARS‐CoV‐2, data represent mean ± SEM (*n* = 4). *p < 0.05, **p < 0.01, ***p < 0.001, ****p < 0.0001 by one‐way ANOVA with Dunnett's multiple comparisons (A, B, E, F, K,L,M), or unpaired two‐tailed Student's *t*‐test (C,G). NS, not significant.

After the SAR campaign, we identified a lead molecule that time‐dependently and dose‐dependently increases FBXO5 protein levels in Beas‐2B cells (**Figure** **6**A,B). BC24877 extended FBXO5 half‐life following a CHX chase and drastically reduced FBXO5 polyubiquitination (Figure 6C,D). Conversely, BC24877 time‐dependently and dose‐dependently enhanced the degradation of SARS‐CoV‐2 NSP7 HiBiT and OC43 NSP7 HiBiT protein (Figure 6E,F; Figure S10A,B, Supporting Information). It also reduced NSP7 half‐life in a CHX chase experiment and drastically increased NSP7 polyubiquitination (Figure 6G,H). As controls, the ubiquitination‐resistant mutants (K44R and S55/58A) of NSP7 were included in Figure 6H, which as expected, were resistant to the drug‐induced ubiquitination of NSP7. Regarding the mechanism of action, we first confirmed the direct target engagement between our compound and FBXO5 through several complementary biophysical assays, including endogenous thermal shift assay (Figure 6I) and microscale thermophoresis (Kd = 2.325 µm) (Figure 6J). Moreover, we found that BC24877 could dose‐dependently disrupt β‐TrCP1/FBXO5 interaction without affecting the levels of β‐TrCP1 (Figure 6K). To further confirm our compound engaged the β‐TrCP1/FBXO5 axis, we tested BC24877 effects in cells with β‐TrCP1 knockdown (Figure 6L). BC24877 dose‐dependently increased FBXO5 and decreased NSP7 in control conditions. β‐TrCP1 knockdown increased baseline FBXO5 levels and decreased baseline NSP7 levels and abrogated any further drug effect. Importantly, BC24877 had no impact on FBXO5 or NSP7 mRNA levels (Figure S11A,B, Supporting Information) and did not alter the protein expression of known downstream substrates of FBXO5 (Figure S11C, Supporting Information). The compound did not interact with β‐TrCP1 through thermal shift assay, nor did it alter the levels of known downstream substrates of β‐TrCP1 (Figure S11D,E, Supporting Information). BC24877 potently reduced OC43 infection in Beas‐2B cells and protects cells from cytopathic effects of OC43 infection (Figure S12A, Supporting Information; Figure 6M). Compound‐treated cells also yielded less viruses spreading in a dose‐dependent fashion (Figure S12B–D, Supporting Information). Finally, we tested the efficacy of BC24877 in VERO cells using both HCoV‐OC43 and SARS‐CoV‐2 (multiple variants). That compound dose‐dependently reduced OC43 infection in VERO cells without affecting cell viability (Figure S13A,B, Supporting Information). Further, this novel host‐centric strategy demonstrated efficacy against multiple SARS‐CoV‐2 variants of concern (VOC), such as WT, *Alpha* and *Beta* with potency comparable to Remdesivir in an immunospot assay in both VERO and Huh7 cells (Figure 6N; Figures S13C–E and S14A–E, Supporting Information).

### 2.7 BC24877 Attenuates HCoV‐OC43 Infection in Human Lung Slices

**Figure 7:**
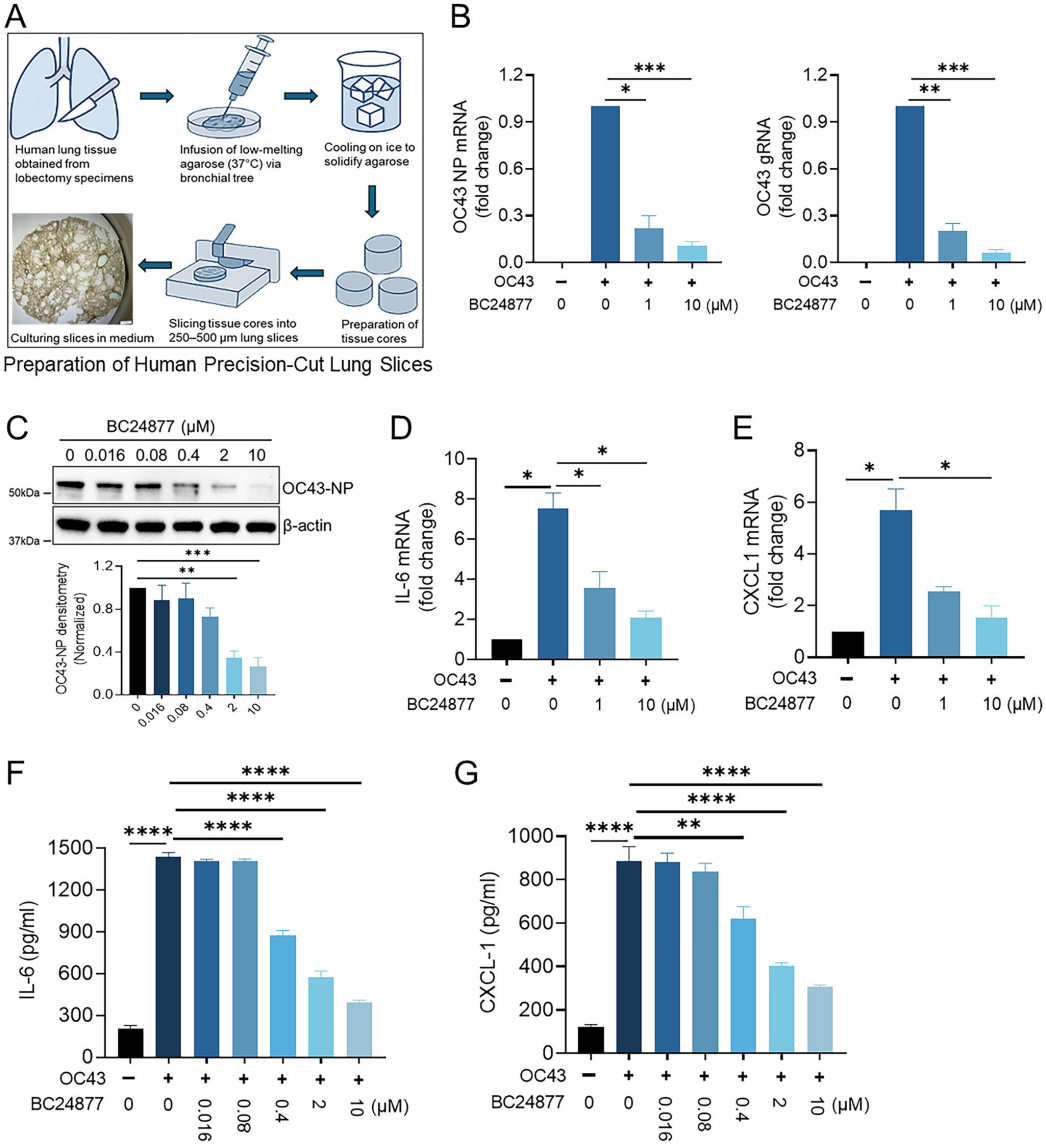
BC24877 attenuates HCoV‐OC43 infection in human lung slices. (A) Schematic Representation of Human Precision‐Cut Lung Slices (hPCLS). (B) RT–qPCR analysis of *OC43 nucleocapsid (NP)* mRNA and *OC43 genomic RNA* (gRNA) levels in hPCLS that were mock stimulated or treated with BC24877 at the indicated dose, or infected with HCoV‐OC43 for 24 h, data represent mean ± SEM (*n* = 3). (C) Immunoblotting of OC43‐NP protein amounts from hPCLS that were treated with BC24877 and OC43 for 24 h, data represent mean ± SEM (*n* = 3). (D) RT–qPCR analysis of *IL‐6* and (E) *CXCL‐1* gene transcriptions in hPCLS stimulated as in (B), data represent mean ± SEM (*n* = 3). (F) ELISA assessment of IL‐6 and (G) CXCL‐1 secretions in the supernatant from hPCLS stimulated as in (B), data represent mean ± SEM (*n* = 3). *p < 0.05, **p < 0.01, ***p < 0.001, ****p < 0.0001 by one‐way ANOVA with Dunnett's multiple comparisons.

We performed additional functional study taking advantage of human ex vivo lung model using Human Precision‐Cut Lung Slices (hPCLS). In this study, human lung tissue was obtained from fresh lobectomy specimens. It was then infused with low‐melting agarose followed by cooling on ice to generate the tissue core (**Figure** **7**A). Precision cut lung slices (250–500 µm) were then prepared from the tissue core and cultured in 24‐well plates. This ex vivo lung model provides an excellent system to test anti‐viral compounds. We incubated the lung slices with compounds along with HCoV‐OC43 for 24 h. Lung tissues were collected and assayed for OC43 viral RNA, viral protein and inflammatory gene expression. We observed potent suppression of OC43 viral RNAs and viral NP protein (Figure 7B,C). Compound also dose‐dependently reduced pro‐inflammatory gene expression (*IL‐6* and *CXCL1*) in lung tissue (Figure 7D,E). Finally, as shown in Figure 7F,G, compound also dose‐dependently reduces IL‐6 and CXCL1 levels in the culturing media measured using ELISA. This ex vivo model suggests that our compound potentially can work well in human lungs.

## 3 Discussion

Current COVID‐19 therapies exclusively focus on targeting SARS‐CoV‐2 itself. While several small molecules have been investigated for their antiviral potential, each presents certain limitations. The antiviral drug molnupiravir directly inhibits viral replication. However, since it functions as a mutagenic ribonucleoside, the long‐term risk of its incorporation into host DNA remains unclear.^[^ ^36^, ^37^ ^]^ Ritonavir‐boosted nirmatrelvir is a SARS‐CoV‐2 viral NSP5 protein inhibitor. This combination therapy contains a CYP inhibitor, which substantially increases the risk of drug‐drug interaction in the elderly (>70) and the heavily medicated population.^[^ ^38^ ^]^ The antiviral drug remdesivir has demonstrated only modest clinical efficacy, with the most significant impact observed in severely ill patients.^[^ ^39^, ^40^ ^]^ Finally, despite the effectiveness of various COVID‐19 vaccines, due to the likelihood of RNA viral mutations, it is doubtful that any current vaccine will be effective against all new strains or future coronavirus outbreaks. It is important to note that there are no available host‐based therapeutics for COVID‐19.

In this study, we selected SARS‐CoV‐2 NSP7 as a potential target to develop therapeutics. NSP7 is a highly conserved viral protein across all coronaviruses, underscoring its essential role in viral replication and transcription.^[^ ^41^, ^42^ ^]^ This evolutionary conservation suggests that NSP7 could serve as a universal therapeutic or diagnostic target for combating not only SARS‐CoV‐2 but also other existing and emerging coronaviruses. While the immediate threat of the COVID‐19 pandemic has subsided, the risk of future coronavirus outbreaks remains significant. Our findings provide foundational insights into the regulation of NSP7, with implications for developing host‐directed therapies to address these enduring challenges.

We uncovered a novel mechanism by which SARS‐CoV‐2 NSP7 modulates the host's antiviral immune response. Our RNA sequencing (RNA‐seq) analysis revealed that NSP7 significantly suppressed antiviral responses in lung epithelial cells. This finding aligns with previous studies that SARS‐CoV‐2 NSP7 can inhibit type I and III IFN production.^[^ ^43^ ^]^ Moreover, through unbiased proteomic studies, we identified ISG15, a ubiquitin‐like protein, as a critical interacting partner of NSP7. ISG15 undergoes covalent conjugation to lysine residues of target proteins through a post‐translational modification process known as ISGylation.^[^ ^44^ ^]^ This modification is rapidly upregulated in response to interferon signaling and plays a critical role in modulating antiviral signaling pathways.^[^ ^45^ ^]^ Notably, ISGylation has been implicated in regulating the stability and functional capacity of antiviral proteins, including MDA5, a critical RNA sensor in the cytosol.^[^ ^46^, ^47^ ^]^ Our subsequent experiments demonstrated that NSP7 markedly inhibited the ISGylation of MDA5. Previous studies have reported that ISGylation is essential for MDA5 activation and its subsequent interaction with the mitochondrial adaptor MAVS, a key component in initiating downstream antiviral signaling cascades through NF‐kappaB and IRF3.^[^ ^46^, ^48^ ^]^ Furthermore, Miah et al. reported that SARS‐CoV‐2 NSP7 shares a homologous epitope with a known Treg suppressor, potentially contributing to immune evasion by attenuating CD4+ and CD8+ T cell responses.^[^ ^49^ ^]^ Collectively, these findings provide new insights into how NSP7 may contribute to immune evasion and could partly explain the delayed viral clearance and disease severity observed in COVID‐19 patients.^[^ ^50^ ^]^

In addition to uncovering NSP7's role in immune evasion, our study identified a novel pathway involved in the degradation of NSP7 via the ubiquitin‐proteasome system. Specifically, we found that the E3 ubiquitin ligase FBXO5 plays a key role in targeting NSP7 for proteasomal degradation in lung epithelial cells. This process is further facilitated by the protein kinase TAF1, which phosphorylates NSP7 thereby accelerating its ubiquitination and degradation. Our findings align with previous research showing that host cells use the ubiquitin‐proteasome system as a crucial strategy to eliminate viral proteins and restrict viral replication.^[^ ^51^, ^52^, ^53^ ^]^ Moreover, we observed that inhibition of β‐TrCP1, a known regulator of the ubiquitin‐proteasome system, can significantly upregulate FBXO5 expression. This observation is consistent with earlier studies demonstrating that β‐TrCP1 can ubiquitinate and degrade FBXO5.^[^ ^20^, ^35^ ^]^ By inhibiting β‐TrCP1, we found that FBXO5 expression is stabilized, enhancing NSP7 degradation. This complex regulatory interaction between β‐TrCP1 and FBXO5 highlights a potential viral evasion strategy, whereby suppression of FBXO5 by β‐TrCP could stabilize NSP7 and prolong its immune‐suppressive functions. Finally, we identified a novel small molecule stabilizer of FBXO5, which not only promotes NSP7 degradation but also effectively reduces viral replication in vitro. Unlike approaches that target the virus directly, modulators of the β‐TrCP1/FBXO5/NSP7 axis aim to enhance intrinsic cellular defense mechanisms. This strategy is particularly appealing as it holds the potential to remain effective against emerging novel viral strains.

Despite the valuable insights our study provides regarding NSP7's role in immune evasion and its degradation via the ubiquitin‐proteasome pathway, there are some limitations to consider. Primarily, our findings are based on in vitro studies, which may not fully reflect NSP7's behavior in other cell types or in vivo. Additionally, while we identified a small molecule activator of FBXO5 that enhances NSP7 degradation and reduces viral replication, its efficacy in vivo remains to be assessed. To overcome some of these limitations, we test our small molecule FBXO5 stabilizer in human ex vivo lung model using Human Precision‐Cut Lung Slices (hPCLS). hPCLS offers a highly relevant ex vivo model of the human lung, retaining its native architecture and cell types. They are valuable for assessing drug efficacy and safety and offer an alternative for animal testing. Our FBXO5 stabilizer exhibited potent activity in this model, blocking OC43 infection and reducing tissue inflammation. Another promising direction for future research includes exploring the detailed regulatory mechanisms of the β‐TrCP1/FBXO5 axis and investigating its role across other viral proteins to gain a more comprehensive understanding of how SARS‐CoV‐2 manipulates host cell pathways for immune evasion.

In summary, our study reveals that SARS‐CoV‐2 NSP7 facilitates immune evasion by suppressing the antiviral response. We identified interactions between NSP7 and ISG15 that impair MDA5 ISGylation and showed that inhibiting β‐TrCP1 enhances FBXO5‐mediated NSP7 degradation. Additionally, a small molecule activator of FBXO5 was found to reduce coronaviral replication. These findings provide insights into the reciprocal host‐viral regulation of the highly conserved, essential viral protein NSP7 and suggest potential new host‐centric therapeutic strategies against coronaviruses.

## 4 Experimental Section

### Reagents

All reagents used in this study are detailed in Table S2 (Supporting Information).

### Cell Lines

BEAS‐2B cells were cultured in HITES medium+10% FBS and Penicillin‐Streptomycin (Gibco, 10 000 U mL^−1^, 15140163). HEK293T cells were cultured in Dulbecco's Modified Eagle's Medium (DMEM) (ATCC® 30‐2002™) medium+10% FBS and Penicillin‐Streptomycin. HCT‐8 (ATCC® CCL‐244™) cells were maintained in RPMI 1640 medium (Gibco, ATCC modification, A1049101), supplemented with 10% FBS (Gibco) and Penicillin‐Streptomycin. VERO cells (ATCC® CCL‐81™) were cultured in Eagle's Minimum Essential Medium (EMEM) (ATCC® 30‐2003™) supplemented with 16% FBS and Penicillin‐Streptomycin. NSP7‐HiBiT stable cell line was prepared by transfecting with the NSP7‐HiBiT plasmid in BEAS‐2B cells using a transfection reagent following the manufacturer's protocol. After 48 h, Geneticin™ Selective Antibiotic (500–800 µg mL^−1^, Gibco) was added to the culture medium to begin selection of stably transfected cells. The selection continued for 1–2 weeks until resistant colonies formed. Single‐cell clones were isolated by limiting dilution to ensure clonal populations. These clones were expanded, and stable expression of NSP7‐HiBiT was confirmed through Western blot or luminescence‐based assays. To generate an FBXO5‐HiBiT CRISPR knock‐in cell line, a CRISPR/Cas9 system was used to introduce the HiBiT tag at the N‐terminus of the endogenous FBXO5 gene. BEAS‐2B cells were co‐transfected with the Cas9 enzyme, guide RNA targeting FBXO5, and a donor template containing the HiBiT sequence flanked by homology arms. After transfection, cells were allowed to recover, and single‐cell clones were isolated through limiting dilution. Clones were expanded, and successful knock‐in was verified by sequencing, as well as through Western blot or luminescence assays to confirm proper FBXO5‐HiBiT expression. The sequences of sgRNA and donor template used in the experiments are provided in Table S3 (Supporting Information).

### Synthesis of BC24877 and Analogs

Details can be found in Supporting Information.

### Viral Propagation and Inoculation

Human coronavirus OC43 (Betacoronavirus 1, ATCC® VR‐1558™, LOT: 70034234) was prepared following a previously established protocol.^[^ ^52^ ^]^ In brief, HCT‐8 cells were cultured in T75 flasks until reaching 90% confluence, after which the cells were washed twice with serum‐free medium. A 300 µL aliquot of stock OC43 virus was diluted in 5 mL of serum‐free medium and applied to the cells for adsorption over 1 h at 34 °C in 5% CO_2_. Following adsorption, 10 mL of serum‐free medium was added to the cells, and the virus was propagated for 5 days under the same conditions. The viral supernatant was harvested by centrifugation at 1000 g for 10 min. Larger virus batches were prepared in the same manner.

### Viral Spreading Assay

The spreading assay was performed as previously described,^[^ ^52^ ^]^ with slight modifications. BEAS‐2B cells, treated with DMSO or the compound of interest, were inoculated with OC43 at a multiplicity of infection (MOI) of 0.03 for 18 h at 34 °C. After the initial infection period, the cells were gently but thoroughly washed three times with fresh media and then incubated for an additional 48 h at 34 °C. After this secondary incubation, equal volumes of the media supernatant were collected and titrated onto naïve WT‐BEAS2B cells. After a further 48‐h incubation, the cells were fixed and immunostained for OC43. Images were captured using the GE InCell2000 imaging system, and the proportion of infected cells was quantified using CellProfiler software.

### Immunospotting Assay

VERO cells were seeded into 96‐well plates at a density of 1 × 10^4^ cells per well and cultured in DMEM supplemented with 10% fetal bovine serum (FBS) and 1% penicillin‐streptomycin at 37 °C with 5% CO₂ overnight. Cells were then infected with SARS‐CoV‐2 (MOI = 1) for 1 h at 37 °C to allow viral adsorption. Following infection, cells were washed three times with phosphate‐buffered saline (PBS) to remove unbound virus and cultured in fresh DMEM containing 2% FBS. At 24 h post‐infection, cells were fixed with 4% paraformaldehyde (PFA) in PBS for 30 min at room temperature, followed by three washes with PBS. Cells were permeabilized by incubating with 0.1% Triton X‐100 in PBS for 10 min and subsequently blocked with 5% bovine serum albumin (BSA) in PBS for 1 h at room temperature to reduce nonspecific binding. Following blocking, cells were incubated with a primary antibody (1:1000 dilution) specific for the SARS‐CoV‐2 nucleocapsid (N) protein overnight at 4 °C. After incubation, cells were washed three times with PBS. Cells were incubated with a horseradish peroxidase (HRP)‐conjugated secondary antibody at a 1:2000 dilution for 1 h at room temperature. Following secondary antibody incubation, cells were washed three times with PBS. For colorimetric detection, cells were developed with a HRP substrate (3,3′,5,5′‐Tetramethylbenzidine, TMB) according to the manufacturer's instructions (Sigma–Aldrich, Cat# T0440), and positive viral spots were visualized under a light microscope. Images were captured, and the number of viral spots per well was quantified to assess infection levels. The number of viral spots per well was counted, and data were normalized relative to control conditions to evaluate infection levels across different experimental treatments.

### Plasmid Preparation and Cloning

SARS‐CoV‐2 NSP7 coding sequences were subcloned into pFC37K‐HiBiT vector or pcDNA3.1D‐V5‐HIS through TOPO cloning. Point mutants (Lysine→Arginine; Serine→Arginine) were generated through QuikChange II XL Site‐Directed Mutagenesis Kit. All plasmid constructs were verified by DNA sequencing (Genewiz).

### Plasmid or siRNA Transfection

Plasmid transfection into BEAS‐2B cells was conducted over a period of two days using either the Nucleofector II system (Amaxa) or the Lipofectamine 3000 transfection reagent. siRNA transfections were carried out using the Lipofectamine RNAiMAX Transfection Reagent according to the manufacturer's protocol. The sequences of all siRNAs used in the experiments are provided in Table S3 (Supporting Information).

### Immunoblotting

Cells were lysed in RIPA buffer (50 mm Tris‐HCl, pH 7.6, 150 mm NaCl, 1% Triton‐X‐100, 0.1% SDS, 0.5% sodium deoxycholate) with protease inhibitors to collect protein samples. The lysates were sonicated (12 s at 20% amplitude) and then centrifuged at 16 000 × g for 15 min at 4 °C. Protein concentrations were quantified, and equalized samples were prepared using reducing Laemmli buffer (final composition: 50 mm Tris‐HCl, pH 6.8, 2% SDS, 10% glycerol, and 100 mm DTT). Primary antibodies were applied according to the manufacturer's instructions, while secondary antibodies were used at a 1:5000 dilution. Blot development was performed with West Femto Maximum Sensitivity Substrate and imaged using ChemiDoc Imaging System from Bio‐Rad. Uncropped blots are provided in Supporting Information.

### RT‐qPCR

Total RNA was isolated using either the RNA Extraction Miniprep Kit (Bioland Scientific) or the RNeasy Plus Mini Kit (Qiagen) according to the manufacturer's instructions. cDNA synthesis was performed with the High‐Capacity RNA‐to‐cDNA Kit (Applied Biosystems). Quantitative PCR (qPCR) was carried out using SYBR Green Real‐Time PCR Master Mixes (Applied Biosystems). Relative mRNA expression levels were normalized to β‐actin and calculated using the ΔΔC_t_ method, with control cells serving as the reference. Primer sequences can be found in Table S3 (Supporting Information).

### RNA Sequencing

BEAS‐2B cells were divided into two groups: the Empty‐V5 plasmid transfection group and the NSP7‐V5 plasmid transfection group. Both groups were stimulated overnight with poly(I:C), after which total RNA was extracted using an RNA Extraction Miniprep Kit. RNA quality and concentration were assessed using a Bioanalyzer (Agilent Technologies) to ensure RNA integrity. RNA samples were then sent to GeneWiz for RNA sequencing. Library preparation was performed using poly‐A selection to enrich for mRNA, followed by cDNA synthesis and adapter ligation. The libraries were sequenced on an Illumina platform, generating paired‐end reads. Subsequent data analysis included quality control, reading alignment to the reference genome, and differential gene expression analysis between the two conditions.

### Enzyme‐Linked Immunosorbent Assay (ELISA)

Human IL‐6, CXCL1, IFN‐β, and IFN‐λ levels in the culture supernatants of BEAS‐2B cells were measured using the Human Quantikine ELISA Kit (R&D Systems), following the manufacturer's instructions.

### Immunoprecipitation

For HiBiT immunoprecipitation, cells were lysed in IP lysis buffer (50 mm Tris, 150 mm NaCl, 0.25% v/v Triton‐X‐100, and 0.1% NP40, pH 7.5), and an equal concentration of total protein (300–500 µg) was incubated with 5 µg anti‐HiBiT antibody overnight at 4 °C with gentle rotation. The following day, 50 µL magnetic beads were pre‐washed and added to the antibody‐lysate mixture and rotated for an additional 2–4 h at 4 °C. For HIS or HA PD, cells were transfected with NSP7‐V5‐HIS, or HA‐FBXO5. After 48 h, cells were lysed in IP lysis buffer and cleared by centrifugation at 16 000 × g for 15 min at 4 °C. Cell lysates were then subjected to HIS or HA PD using magnetic agarose beads (Pierce) at 4 °C for 16 h rotation. After rotation, the beads were washed 3–5 times with lysis buffer to remove non‐specifically bound proteins. The immunoprecipitated proteins were eluted in 1x Laemmli SDS Sample Buffer at 95 °C for 10 min and resolved through SDS‐PAGE immunoblotting.

### Cell Viability Assessment

Cell viability was tested using CellTiter‐Glo 2.0 Cell Viability Assay (Promega). 20 µL of reagent was dispensed directly into each well of the 384‐well tissue culture plates prior to luminescence signal acquisition by Cytation 5 plate reader.

### RNAi Library Screening

RNAi screening of SARS‐CoV‐2 NSP7‐HiBiT expressing BEAS‐2B cells with library targeting components of the ubiquitination pathway or kinases was conducted similar to the previous description.^[^ ^54^ ^]^ Briefly, 0.2 µg of esiRNA (Sigma) was diluted with Opti‐MEM and mixed with Lullaby reagent for 20 min before mixture with BEAS‐2B cells stably expressing NSP7‐HiBiT to a final density of 1500 cells per well in 384‐well glass bottom plates. After 72 h of silence, cells were analyzed with Nano‐Glo HiBiT Lytic Detection System and CellTiter‐Glo 2.0 Cell Viability Assay. Luminescent signals were normalized to control siRNA signal.

### Compound Library Screening

A custom diversity library of 12 800 compounds was stamped to white Nunc 384 well plate (custom Agilent pin tool system, 50 nL per well). FBXO5‐HiBiT BEAS‐2B cells were then seeded to a final density of 4000 cells per well (50 µL total volume). After 18 h of treatment, cells were analyzed with Nano‐Glo HiBiT lytic detection system (Promega) to measure luminescent signal.

### Immunocytochemistry

BEAS‐2B or VERO cells were seeded into 384‐well glass‐bottom plates (Cellvis) at a density of 5000 cells per well and subjected to the indicated treatments. Viral inoculations were conducted for 24–48 h prior to sample collection. Following treatment, cells were fixed with 4% paraformaldehyde for 30 min and permeabilized with 0.3% Triton X‐100 for 1 h. After permeabilization, the buffer was discarded, and OC43 antibody (1:2000 dilution) was added to each well, followed by overnight incubation at 4 °C. The following day, the plates were washed four times with 0.5% TBST, then incubated with Hoechst 33342 and a secondary antibody (1:2000 dilution) for 1 h at room temperature. Afterward, the plates were washed again four times with 0.5% TBST. Fluorescent signals were acquired using the GE InCell2000 imaging system, and the proportion of infected cells was quantified using CellProfiler.^[^ ^55^ ^]^

### Cellular Thermal Shift Assay

Our procedure was based on previous literature.^[^ ^56^ ^]^ BEAS‐2B cells were collected and resuspended in 1 mL of Opti‐MEM™ I Reduced‐Serum Medium, supplemented with either vehicle or BC24877 (1 µm). The cell suspensions were then evenly distributed into 12 PCR microtubes and incubated at 37 °C for 30 min, with gentle vortexing every 5 min. Following incubation, each aliquot was subjected to a temperature gradient using a PCR thermocycler, with temperatures ranging from 40 to 60 °C at specified intervals for 3 min, followed by a 3‐min incubation at room temperature. Immediately afterward, the samples were snap‐frozen in liquid nitrogen, followed by two freeze‐thaw cycles. After a brief vortex, the samples were centrifuged at 20 000 g at 4 °C for 20 min. The supernatants were carefully collected for subsequent immunoblotting analysis.

### Microscale Thermophoresis

For microscale thermophoresis (MST) experiments, purified FBXO5 was fluorescently labeled with the amine‐reactive dye RED‐MALEIMIDE 2nd Generation (NanoTemper Technologies) according to the manufacturer's instructions. After labeling, FBXO5 was diluted in PBS as the assay buffer for MST experiments. The diluted FBXO5 was titrated with a two‐fold dilution series of compound starting at 25 µm prior to the samples being transferred to Monolith NT. Automated premium coated capillary chips (NanoTemper Technologies). MST traces were recorded at room temperature in a Monolith NT. Automated using the MO.Control software (LED excitation power setting 20%, medium MST power). The initial fluorescence intensity data were analyzed using MO.Affinity Analysis software.

### In Vitro Ubiquitin Assay

FBXO5‐mediated in vitro ubiquitination assay of SARS‐CoV‐2 NSP7 was conducted as previously described.^[^ ^57^ ^]^ HA‐tagged FBXO5 (pcDNA3.1D) was synthesized using TnT kit (Promega). This FBXO5 was directly incubated with recombinantly‐derived HIS‐tagged NSP7 (R&D Systems, 10632‐CV) along with the full complement of ubiquitination consitutents: E1 (100 nm), mixture of E2 (≈1 µm), ubiquitin (2.5 µm), magnesium/ATP (5 mm) (from Ubiquitinylation kit, BML‐UW9920) and recombinant human CUL1/RBX1 (≈10 nm) (R&D Systems, E3‐411). This assay mixture was incubated for 90 min at 30 °C. Assay was quenched with 3xProtein Sample Buffer and analyzed by SDS‐PAGE and immunoblotting for NPS7‐HIS signal.

### NSP7 Mass Spectrometry

NSP7‐V5 BEAS‐2B cells or control BEAS‐2B cells were lysed in IP lysis buffer (50 mm Tris, 150 mm NaCl, 0.25% v/v Triton‐X‐100, and 0.1% NP40, pH 7.5), and an equal concentration of total protein (500 µg) was incubated with 5 µg anti‐V5 antibody overnight at 4 °C with gentle rotation. On the following day, 50 µL of pre‐washed magnetic beads were added to the antibody‐lysate mixture and incubated with rotation for an additional 4 h at 4 °C. The supernatant was subsequently removed, and the beads were retained. The beads were then washed four times with IP lysis buffer, discarding the wash solution after each step, to obtain the final bead sample. Protein samples were prepared using Preomics iST 96x kit and analyzed with a nanoElute2 HPLC with TimsTOF HT mass spectrometer. Protein identification and quantification analysis were done with PaSER (2023, v 3.0, Bruker Scientific LLC, Billerica, MA) using TIMS DIA‐NN. A spectral library consisting of 80,179 precursors derived from 9095 proteins, including peptide modifications such as phosphorylation, ubiquitinization/sumoylation, and oxidized methionine's was reannotated against Uniprot human protein database plus sequences of known contaminants. 20 ppm precursor tolerance and 15 ppm fragment ion tolerance were used along with Top 3 precursors for quantitation. Multiple samples were assembled and match‐between‐runs performed to fill‐in missing values with an outlier frequency of 0.2 following which global normalization was performed.

### Preparation of Human Precision‐Cut Lung Slices

Precision lung cut slices (PCLS) were generated as previously described.^[^ ^58^ ^]^ Briefly, deidentified lungs that were deemed unsuitable for transplantation and screened for acute and chronic diseases were collected by The Human Lung Tissue Biobank of the Division of Pulmonary, Allergy, Critical Care, and Sleep Medicine of the University of Pittsburgh. They were maintained in ice‐cold Dulbecco's Modified Eagle Medium (DMEM) and processed within 6 h after resection. Typically, the left medial or lower lobes were used to create the PCLS. The tissue was first examined for macroscopic characteristics and pleural integrity. The selected lobe was then flushed with sterile saline supplemented with 1% Penicillin‐Streptomycin (PenStrep) and 2.5 µg mL^−1^ of Amphotericin B via the main bronchus to remove excess blood. Following this, the lobe was inflated at a constant pressure and speed of ≈1 mL sec^−1^ with a solution of 2.5% low‐melting point agarose in DMEM/F12 cell culture media, which was also supplemented with 1% PenStrep, 2.5 µg mL^−1^ of Amphotericin B, and 1% heat‐inactivated fetal bovine serum (Supplemented DMEM/F12) kept at a temperature of 37–40 °C. After inflation, the tissue was placed in a bag and kept on ice for 40–45 min to allow the agarose to solidify. Once the agarose had jellified, the tissue was placed on a cutting board and sliced sagittally into 2.5 cm thick sections using a trimming blade, then examined for abnormalities and consistency of filling. Cylindrical tissue cores, measuring 10 mm in diameter, were excised from the lung parenchyma using a tissue coring tool (Alabama Research and Development, Munford, AL). The cores were collected and stored in supplemented DMEM/F12 at 4 °C until sliced, usually within the next 24 h. PCLS measuring 1 cm in diameter and 300 µm in thickness were produced from the tissue cores using a Compresstome vibroslicer (Precisionary Instruments LLC). The PCLS were collected in ice‐cold supplemented DMEM/F12 and distributed onto 24‐well tissue culture plates, where they were maintained under standard tissue culture conditions (37 °C, 95% O2, high humidity) in supplemented DMEM/F12 for the first 24 h. The following day, the culture medium was replaced with DMEM/F12 media supplemented with 1% PenStrep, 2.5 µg mL^−1^ of Amphotericin B, and 0.1% heat‐inactivated fetal bovine serum, with media changes occurring every other day throughout the culture period. Sterility was maintained during the entire process, and controls were included to monitor potential sources of microbial contamination, such as reagents used for processing (e.g., low‐melt agarose, saline, and culture media), which were sampled over time.

### Statistical Analysis

Statistical analyses were conducted using GraphPad Prism 9. Detailed information regarding the statistical methods employed in each experiment is provided in the figure legends. An unpaired two‐tailed Student's *t*‐test was used for comparisons between two groups, while one‐way ANOVA with Dunnett's multiple comparisons tests was applied for comparisons involving more than two groups. Statistical significance was defined as *p* < 0.05, with specific *p*‐values indicated in the figure legends.

## Conflict of Interest

The authors declare no conflict of interest.

## Author Contributions

B.B.C. and Y.T. designed and directed the study. Y.T., Y.L., T.F., and B.B.C. analyzed the data, prepared the figures and wrote the manuscript. Y.C., T.B.L., F.T., J.J.V., A.N.B., B.L., I.A., J.R.K., M.B.L., Y.H., Y.C., M.E.C., S.M.B., J.W.E., and R.P., performed all the experiments. D.P.C. assisted with high‐throughput screening. Y.L., T.F., and B.B.C. provided funding for the studies.

## Supporting information



Supporting Information

Supporting Information

## Data Availability

The data that support the findings of this study are openly available in PubMed at https://www.ncbi.nlm.nih.gov/bioproject/?term=PRJNA1206177, reference number 1206177.
