# Supplementary material for: NSP7 Molecular Degrader Attenuates Coronaviral Infection Through the β‐TrCP1/FBXO5 Axis
Source: Adv Sci (Weinh). 2025 Jun 27;12(36):e00798. doi: 10.1002/advs.202500798 (PMC12463105; doi:10.1002/advs.202500798)
Supplement: Supplementary file 1 — Supporting Information [file ADVS-12-e00798-s001.docx]

NSP7 Molecular Degrader Attenuates Coronaviral Infection Through the β-TrCP1/FBXO5 Axis

Yao Tong^1^, Travis B. Lear^1^, Ferhan Tuncer^1^, John J. Villandre^1^, Áine N. Boudreau^1^, Bo Lin^1^, Irene Alfaras^1^, Jason R. Kennerdell^1^, Daniel P. Camarco^1^, Mads B. Larsen^1^, Yun Hua^1^, Yanwen Chen^1^, Meigin E. Chandler^1^, Ricardo Pineda^2^, Simon M. Barratt-Boyes^3^, John W. Evankovich^1,2^, Toren Finkel^1,4^, Yuan Liu^1,2^, Bill B. Chen^1,2*^

^1^Aging Institute, University of Pittsburgh/UPMC, Pittsburgh, PA 15219, USA

^2^Department of Medicine, Division of Pulmonary, Allergy and Critical Care Medicine, Acute Lung Injury Center of Excellence, University of Pittsburgh, Pittsburgh, PA 15213, USA

^3^Department of Infectious Diseases and Microbiology, School of Public Health, University of Pittsburgh; Pittsburgh PA 15213, USA

^4^Department of Medicine, Division of Cardiology, University of Pittsburgh; Pittsburgh, PA 15213, USA

*Corresponding Author:

Bill B. Chen, PhD

E-mail: chenb@upmc.edu

**This supplementary information file includes:**

**1)** Supplementary Figures 1 to 14 (Figures S1 to S14) and their legends

**2)** Description for Synthesis of BC24877 and Analogues

**3)** Supplementary Table 1. Synthesis and SAR Evaluation of Novel Compounds

**4)** Supplementary Table 2. Antibody and Resources

**5)** Supplementary Table 3. Oligonucleotides

**Supporting Information**

**
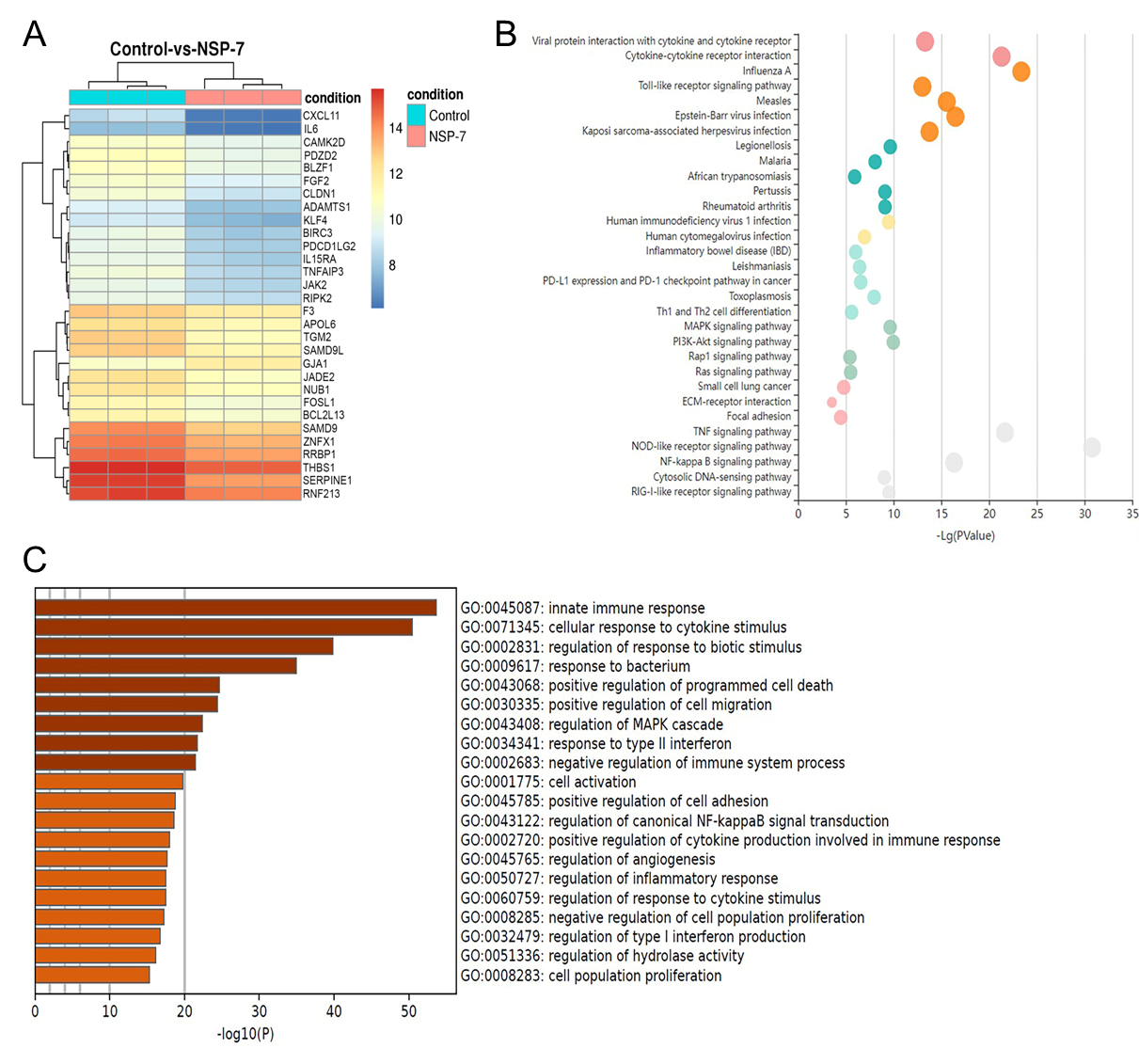
Supplementary Figures and Legends**

**Supplementary Figure S1 related to Figure 1.** **SARS-CoV-2 NSP7 affects host viral signaling pathways.** (A) A heatmap was generated to visualize the top 30 most significantly differentially expressed genes (DEGs) in BEAS-2B cells transfected with empty or NSP7 plasmid for 2 days, followed by overnight treatment with Poly (I:C). (B) KEGG pathway analysis was performed on significant DEGs. (C) GO (biological processes) analysis of significant DEGs.


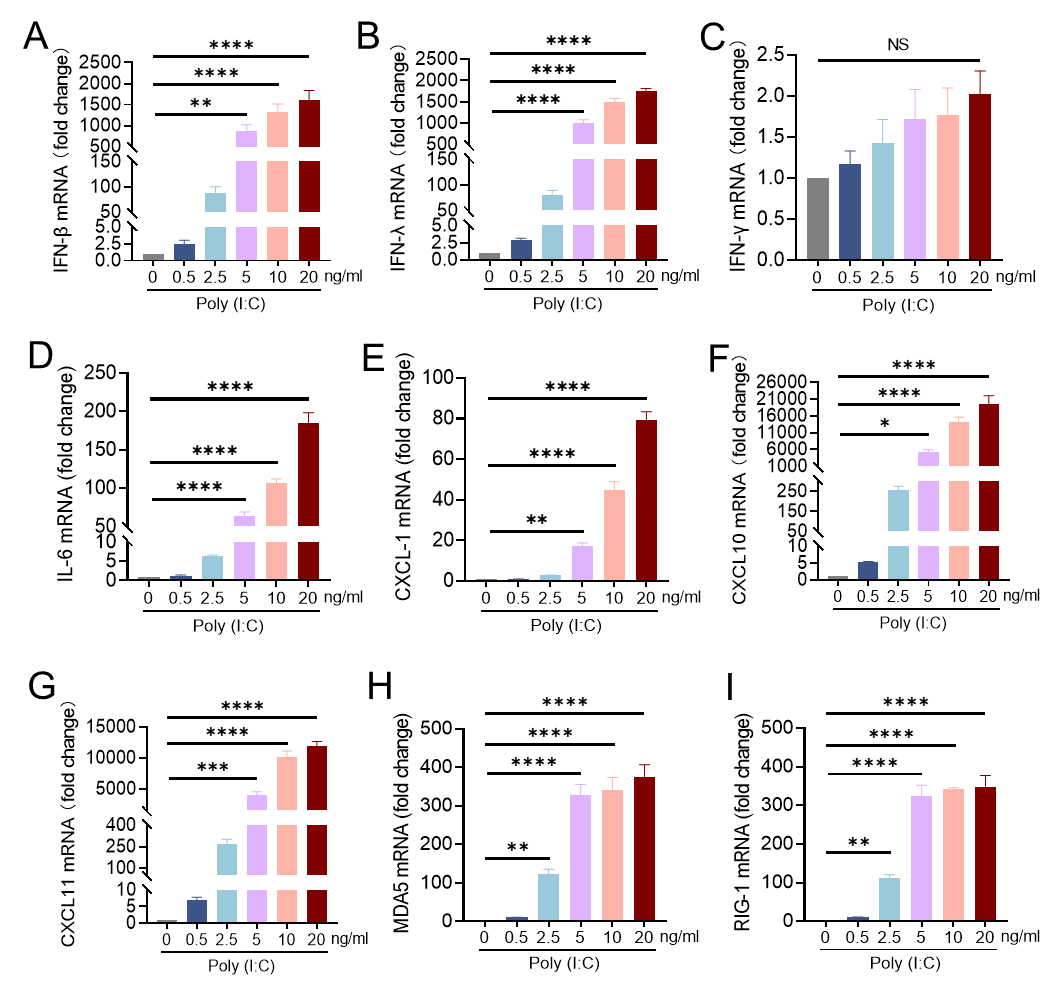
**Supplementary Figure S2 related to Figure 1.** **Poly (I:C) promotes antiviral immune responses in BEAS-2B cells.** (A) *IFN-β*, (B) *IFN-λ*, (C) *IFN-γ*, (D) *IL-6*, (E) *CXCL-1*, (F) *CXCL10*, (G) *CXCL11*, (H) *MDA5*, and (I) *RIG-1* mRNA levels were determined by qPCR in BEAS-2B cells treated overnight with poly (I:C) at the indicated dose, data represent mean ±SEM (n=3). **p* < 0.05, ***p* <0.01, ****p* <0.001, *****p* <0.0001 by one-way ANOVA with Dunnett’s multiple comparisons (A-I). NS, not significant.


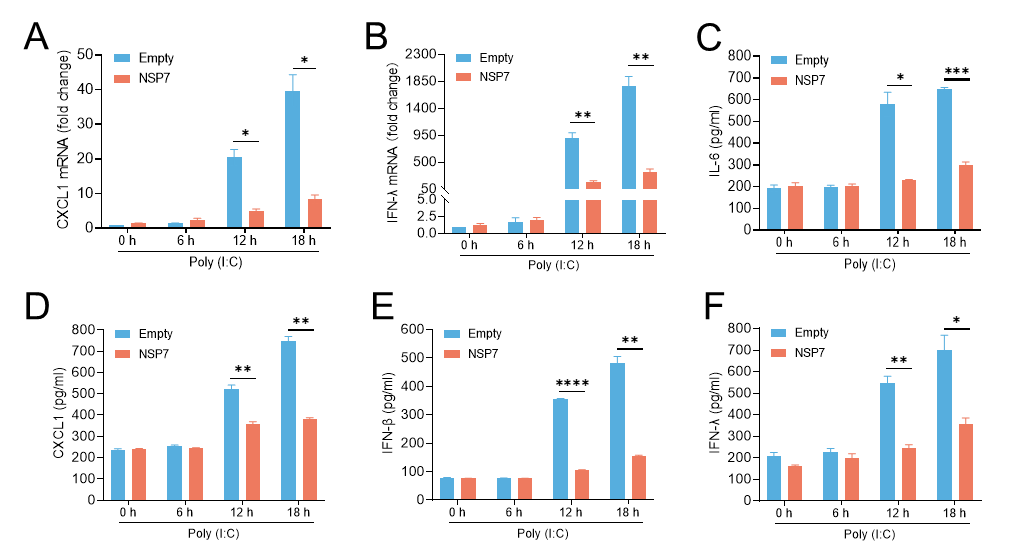
**Supplementary Figure S3 related to Figure 1.** **SARS-CoV2** **NSP7 suppresses antiviral immune response in BEAS-2B cells treated with poly (I:C).** (A) *CXCL-1*, and (B) *IFN-λ* mRNA levels were measured by qPCR in BEAS-2B cells treated with poly (I:C) at the indicated time points, data represent mean ±SEM (n=3). (C) IL-6, (D) CXCL-1, (E) IFN-λ, and (F) IL-6 secretion levels were detected by ELISA in BEAS-2B cells treated with poly (I:C) at the indicated time points, data represent mean ±SEM (n=3). **p* < 0.05, ***p* <0.01, ****p* <0.001, *****p* <0.0001 by unpaired two-tailed Student’s *t*-test (A-F).


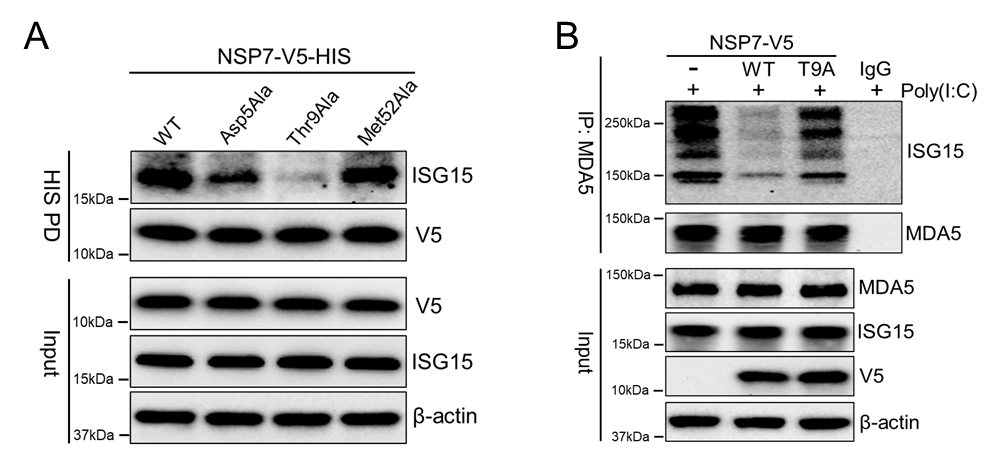


**Supplementary Figure S4 related to Figure 1. SARS-CoV2 NSP7 binds to ISG15 and de-ISGylates MDA5.** (A) Binding assay of NSP7-V5-HIS in BEAS-2B cells that were transfected with V5-HIS-tagged NSP7 WT or mutants, assessed by HIS PD and IB with anti-ISG15 at 48 h post-transfection. (B) Endogenous MDA5 ISGylation in BEAS-2B cells that were transfected with V5-tagged NSP7 WT or T9A mutant for 30 h, followed by 18 h of Poly (I:C) (5 ng/ml) transfection, determined by IP with anti-MDA5 and IB with anti-ISG15.


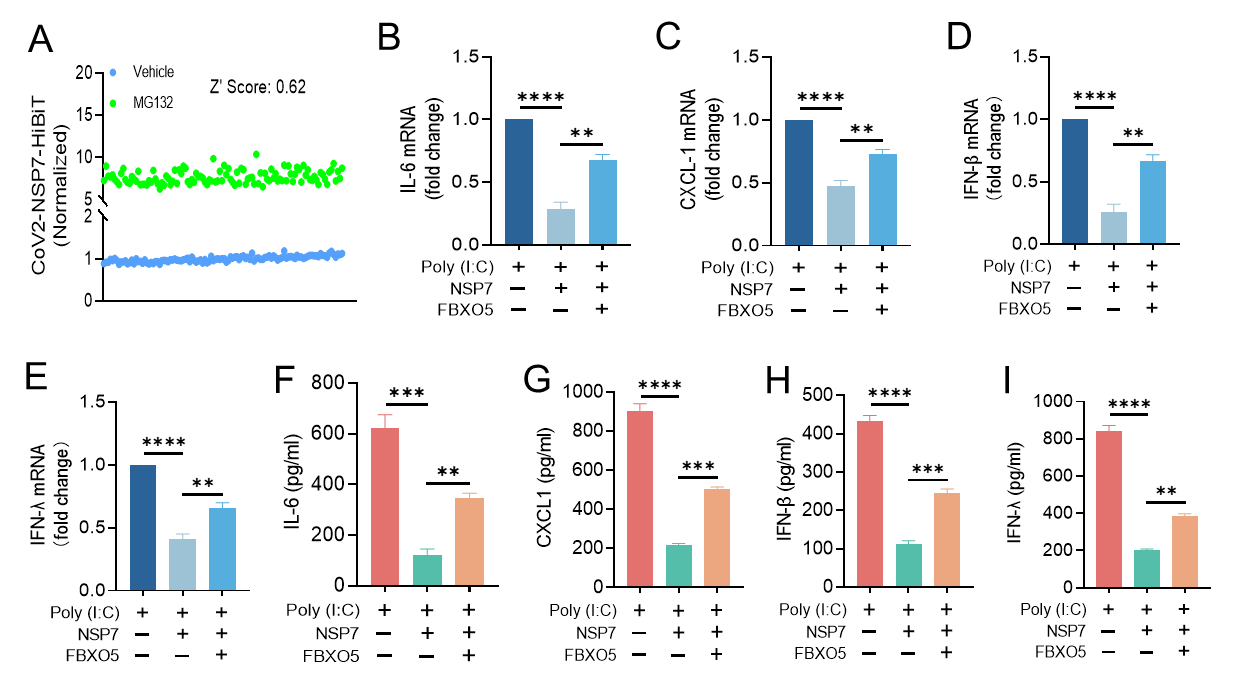
**Supplementary Figure S5 related to Figure 2.** **FBXO5 negates the antagonistic effects of NSP7 on antiviral immune responses in BEAS-2B cells stimulated with poly (I:C).** (A) Z-score for the BEAS-2B cell line stably expressing NSP7-HiBiT. (B) *IL-6*, (C) *CXCL-1*, (D) *IFN-β*, and (E) *IFN-λ* mRNA levels were determined by qPCR in BEAS-2B cells with ectopic expression of NSP7 and/or FBXO5, followed by overnight exposure to poly(I:C), data represent mean ±SEM (n=3). (F) IL-6, (G) CXCL-1, (H) IFN-β, and (I) IFN-λ secretion levels were measured by ELISA in BEAS-2B cells, data represent mean ±SEM (n=3). ***p* <0.01, ****p* <0.001, *****p* <0.0001 by one-way ANOVA with Dunnett’s multiple comparisons (B-I).


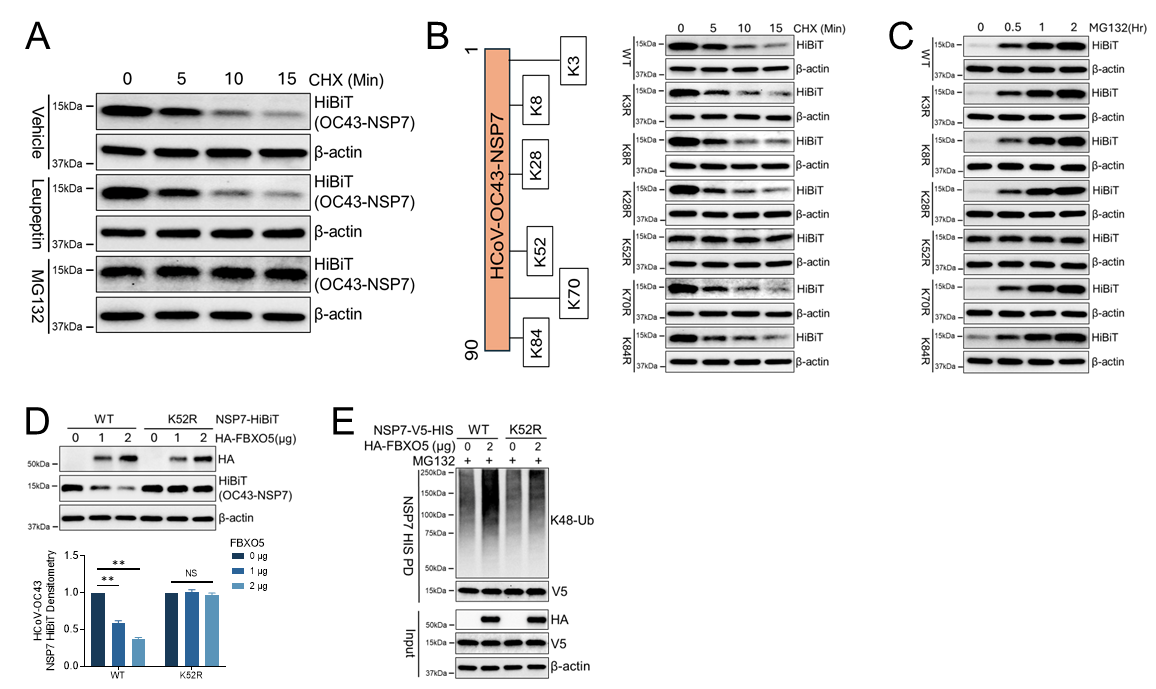


**Supplementary Figure S6 related to Figure 2. The E3 ligase complex SCFFBXO5 mediates HCoV-OC43 NSP7 degradation.** (A) Immunoblotting of HiBiT protein abundance in BEAS-2B cells transfected with the OC43 NSP7-HiBiT plasmid for 48 hours (h), followed by treatment with CHX, Leupeptin, or MG132. (B) Immunoblotting of HiBiT protein amount from NSP7-WT-HiBiT and NSP7 lysin mutant-HiBiT cells treated with CHX or (C) MG132. (D) Immunoblot analysis for NSP7-HiBiT protein level in NSP7-WT-HiBiT and K52R-HiBiT BEAS-2B cells with increasing expression of FBXO5, data represent mean ±SEM (n=3). (E) A cell-based ubiquitination assay in BEAS-2B cells transfected with HA-tagged FBXO5 and V5-HIS-tagged NSP7 WT or K52R mutant for 48 h, followed by 4 h of MG132 treatment. NSP7 was pulled down using HIS-tag magnetic beads, and K48-linked ubiquitination was detected by immunoblotting with an anti-K48 ubiquitin antibody. ***p* <0.01 compared to vehicle or control or as indicated by unpaired two-tailed Student’s *t*-test (D). NS, not significant.


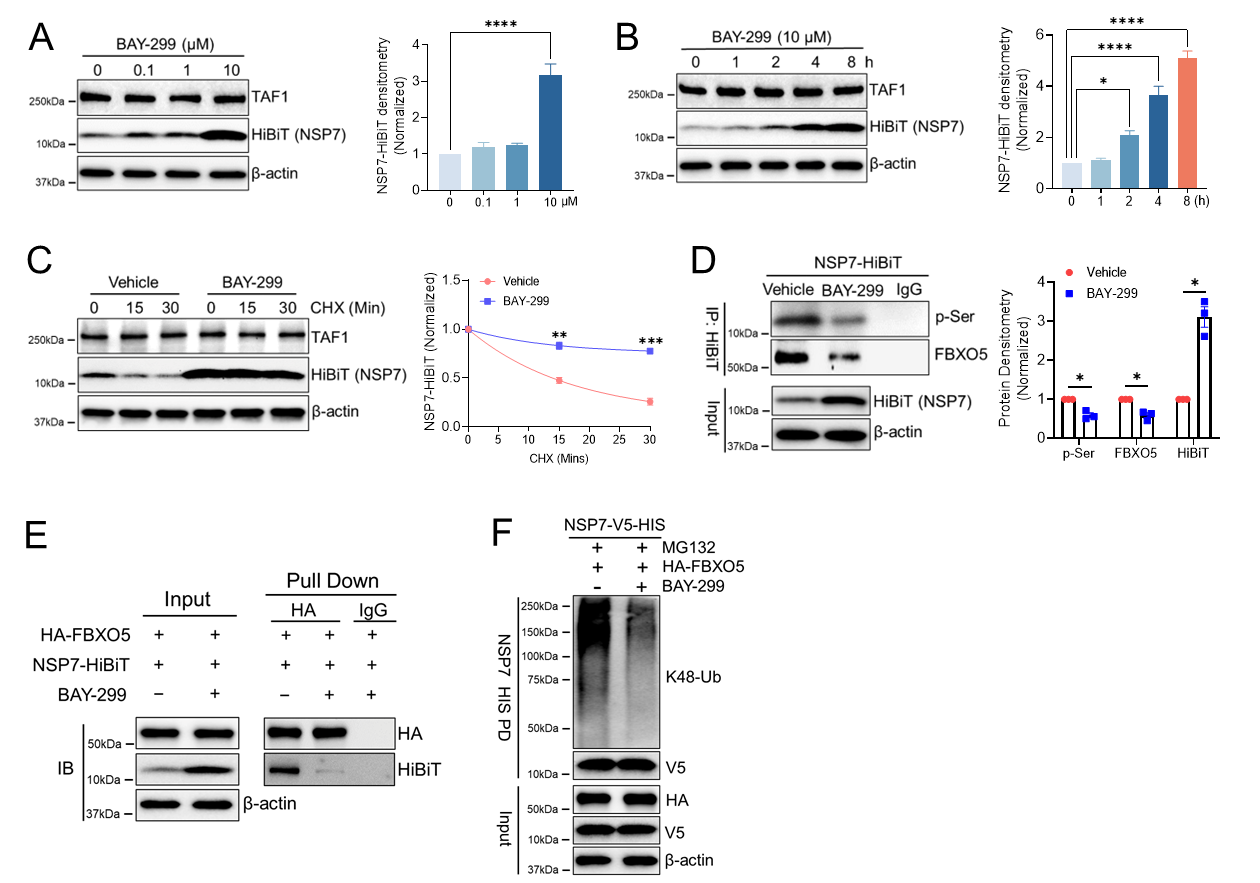
**Supplementary Figure S7 related to Figure 3.** **TAF1 inhibitor BAY-299 prevents the degradation and ubiquitination of SARS-CoV2 NSP7.** (A) Immunoblotting of NSP7-HiBiT protein abundance from NSP7-HiBiT-BEAS-2B cells treated with BAY-299 in a dose-dependent (8 h) or (B) time-dependent manner, data represent mean ±SEM (n=3). (C) Immunoblot analysis of HiBiT protein abundance from NSP7-HiBiT-BEAS-2B cells exposed to BAY-299 (10 μM) for 8 h, followed by CHX treatment, data represent mean ±SEM (n=3). (D) Immunoprecipitation (IP) assay for phospho-serine (p-ser) and FBXO5 protein levels in NSP7-HiBiT-BEAS-2B cells treated with BAY-299. NSP7-HiBiT was immunoprecipitated prior to washing and immunoblotting, data represent mean ±SEM (n=3). (E) A binding assay was performed for NSP7-HiBiT using HA-tag magnetic beads to pull down HA-FBXO5. (F) A cell-based ubiquitination assay was performed in BEAS-2B cells expressing V5-HIS-tagged NSP7 and HA-tagged FNXO5. Cells were treated with vehicle or BAY-299 (10 μM) for 8 h, followed by MG132 treatment for 4 hours. NSP7 was pulled down using HIS-tag magnetic beads, and K48-linked ubiquitination was detected by immunoblotting with an anti-K48 ubiquitin antibody. **p* < 0.05, ***p* <0.01, ****p* <0.001, *****p* <0.0001 by one-way ANOVA with Dunnett’s multiple comparisons (A and B), or unpaired two-tailed Student’s *t*-test (C and D).


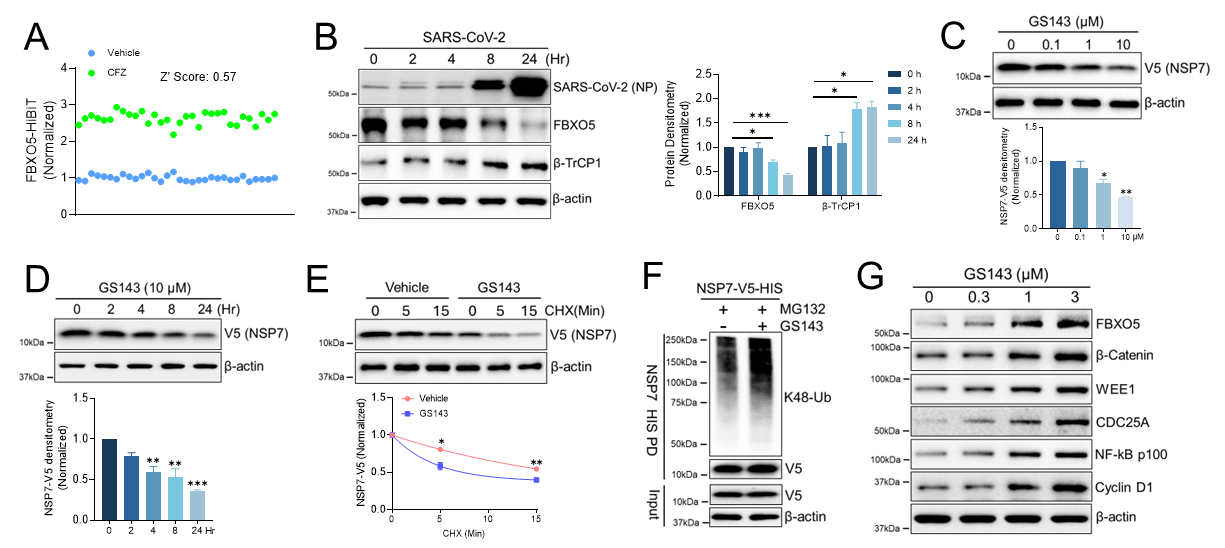
**Supplementary Figure S8 related to Figure 4.** **β-TrCP1 inhibitor GS143 promotes the degradation and ubiquitination of SARS-CoV2 NSP7.** (A) The Z-score for the BEAS-2B cell line with a CRISPR knock-in of the HiBiT tag within the FBXO5 gene. CFZ refers to Carfilzomib. (B) Immunoblotting of FBXO5 and β-TrCP1 proteins abundance from 293T cells infected with SARS-CoV-2 in a time-dependent manner, data represent mean ±SEM (n=3). (C) Immunoblotting of NSP7-V5 protein abundance from BEAS-2B cells treated with GS143 in a dose-dependent (24 h) or (D) time-dependent manner, data represent mean ±SEM (n=3). (E) Immunoblot analysis of NSP7-V5 protein abundance in BEAS-2B cells transfected with the NSP7-V5 plasmid. Cells were treated with GS143 for 24 hours, followed by CHX treatment, data represent mean ±SEM (n=3). (F) A cell-based ubiquitination assay was performed in BEAS-2B cells expressing V5-HIS-tagged NSP7. Cells were treated with vehicle or GS143 (10 μM) for 24 h, followed by MG132 treatment for 4 hours. NSP7 was pulled down using HIS-tag magnetic beads, and K48-linked ubiquitination was detected by immunoblotting with an anti-K48 ubiquitin antibody. (G) Immunoblotting of β-TrCP1 substrates in BEAS-2B cells treated with GS143 for 24 h. **p* < 0.05, ***p* <0.01, ****p* <0.001 compared to vehicle or control or as indicated by one-way ANOVA with Dunnett’s multiple comparisons (B, C, and D), or unpaired two-tailed Student’s *t*-test (E).

**Supplementary Figure S9 related to Figure 5. Cell Viability Assessment of Top-Ranked Compounds.** Cell viability was measured using the Promega® CellTiter-Glo® 2.0 assay in BEAS-2B cells treated with compounds at the indicated dose for 24 h, data represent mean ±SEM (n=3).


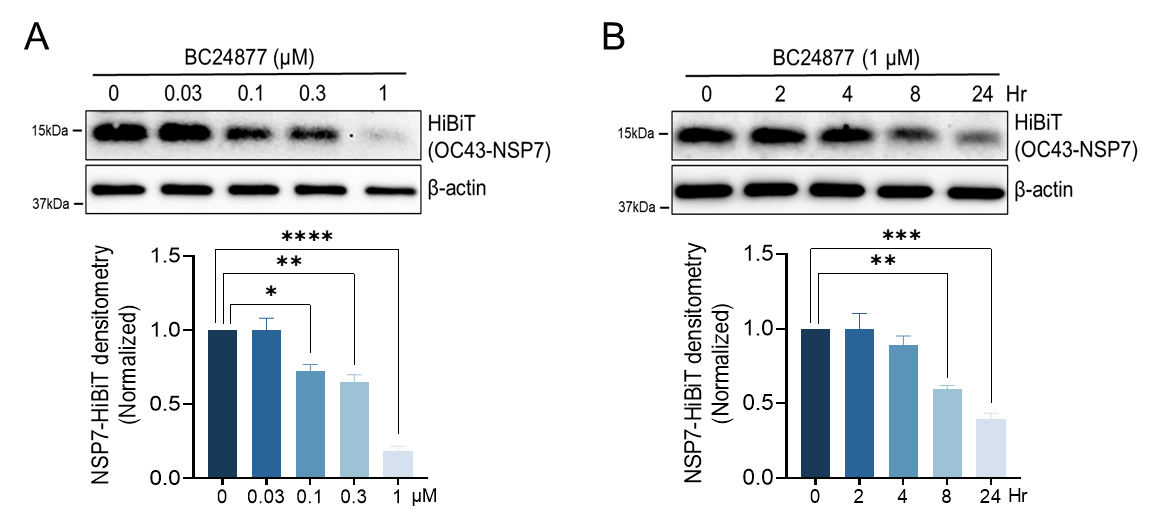


**Supplementary Figure S10 related to Figure 6.** **FBXO5 stabilizer BC24877 inhibits HCoV-OC43 NSP7 expression.** (A) Immunoblotting of OC43-NSP7 protein abundance in BEAS-2B cells transfected with the OC43 NSP7-HiBiT plasmid. Cells were treated with BC24877 in a dose-dependent (24 h) or (B) time-dependent manner, data represent mean ±SEM (n=3). **p* < 0.05, ***p* <0.01, ****p* <0.001, *****p* <0.0001 compared to vehicle or control or as indicated by one-way ANOVA with Dunnett’s multiple comparisons.


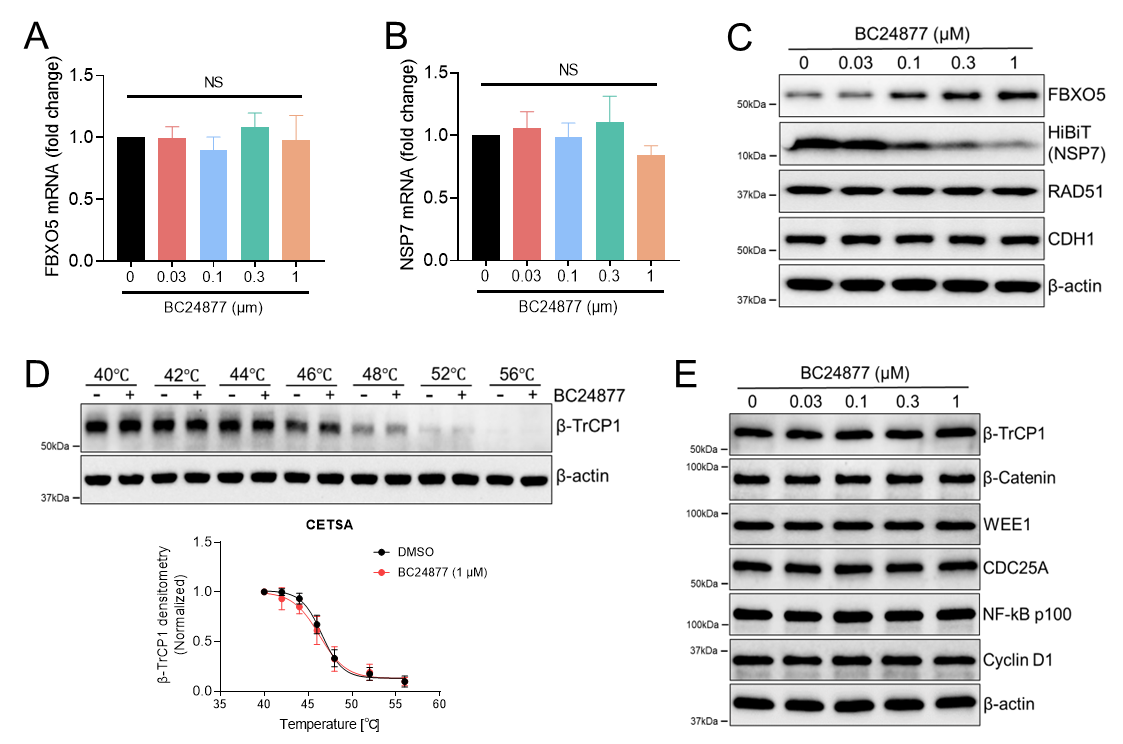
**Supplementary Figure S11 related to Figure 6.** **BC24877 has no effect on the expression of FBXO5 and β-TrCP1 substrates.** (A) *FBXO5*, and (B) *SARS-CoV2-NSP7* mRNA levels were determined by qPCR in BEAS-2B cells treated with BC24877 at the indicated dose for 24 h, data represent mean ±SEM (n=3). (C) Immunoblotting of FBXO5 substrates in BEAS-2B cells treated with BC24877 at the indicated dose for 24 h. (D) CETSA analysis of β-TrCP1 protein in BEAS-2B cell treated with vehicle or BC24877 (1 μM), data represent mean ±SEM (n=3). (E) Immunoblotting of β-TrCP1 substrates in BEAS-2B cells treated with BC24877 for 24 h. NS, not significant.


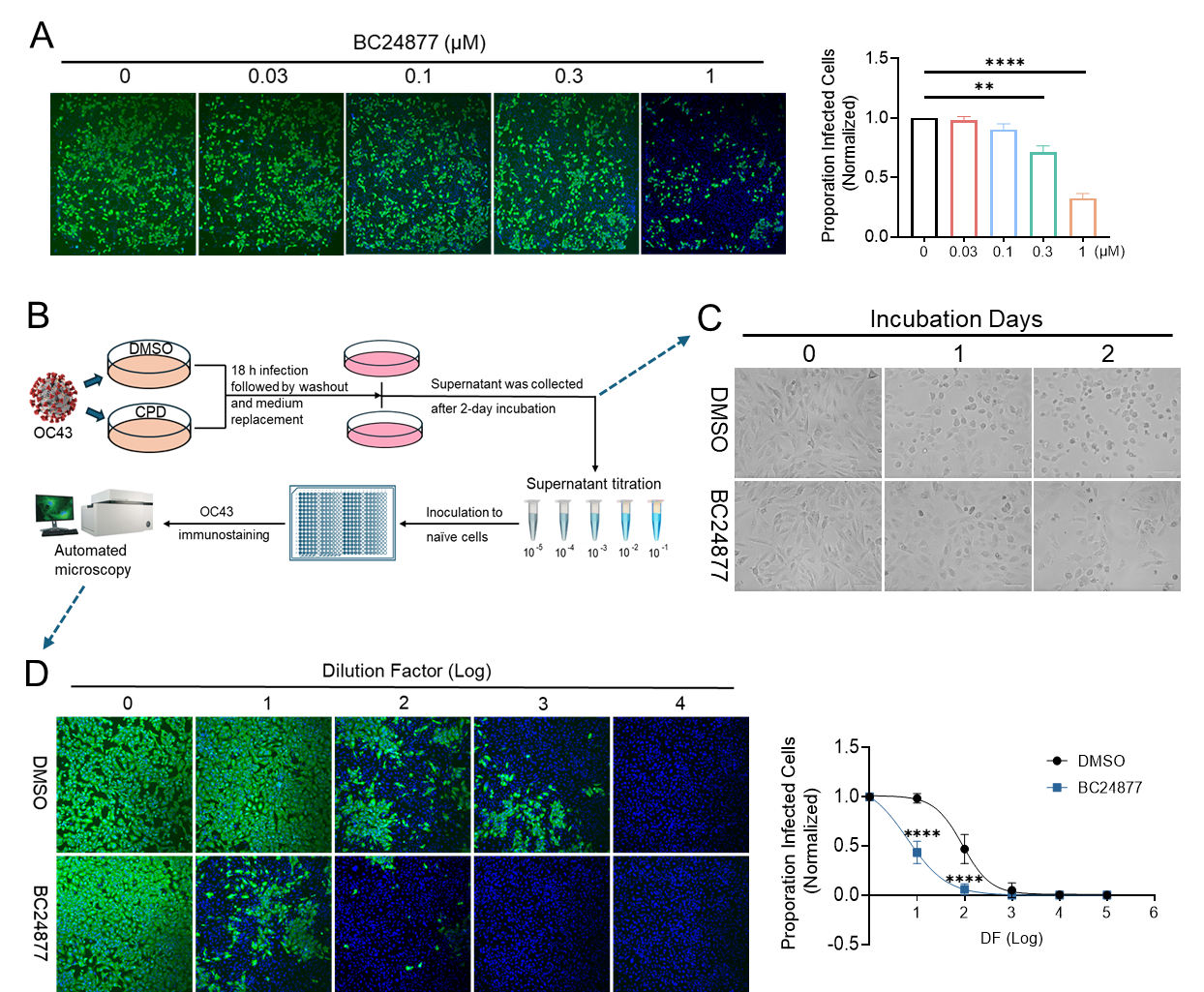


**Supplementary Figure S12 related to Figure 6.** **FBXO5 stabilizer inhibits HCoV-OC43 infection in BEAS-2B cells.** (A) Immunofluorescence staining of OC43 in BEAS-2B cells treated overnight with BC24877 at the indicated dose, data represent mean ±SEM (n=3). (B) Treatment schematic for OC43 spreading assay. Vehicle-treated or CPD (BC24877, 1 μM) -treated BEAS-2B cells were inoculated with equal amounts of OC43 before washout and continued incubation. Supernatant was titrated onto naïve BEAS-2B cells, incubated, and processed for immunofluorescent staining of OC43. (C) Cell morphology of BEAS-2B cells treated with vehicle or BC24877. (D) Immunofluorescence staining of OC43 in naïve BEAS-2B cells, data represent mean ±SEM (n=14). ***p* <0.01, *****p* <0.0001 by one-way ANOVA with Dunnett’s multiple comparisons (A), or unpaired two-tailed Student’s *t*-test (D). CPD, compound.


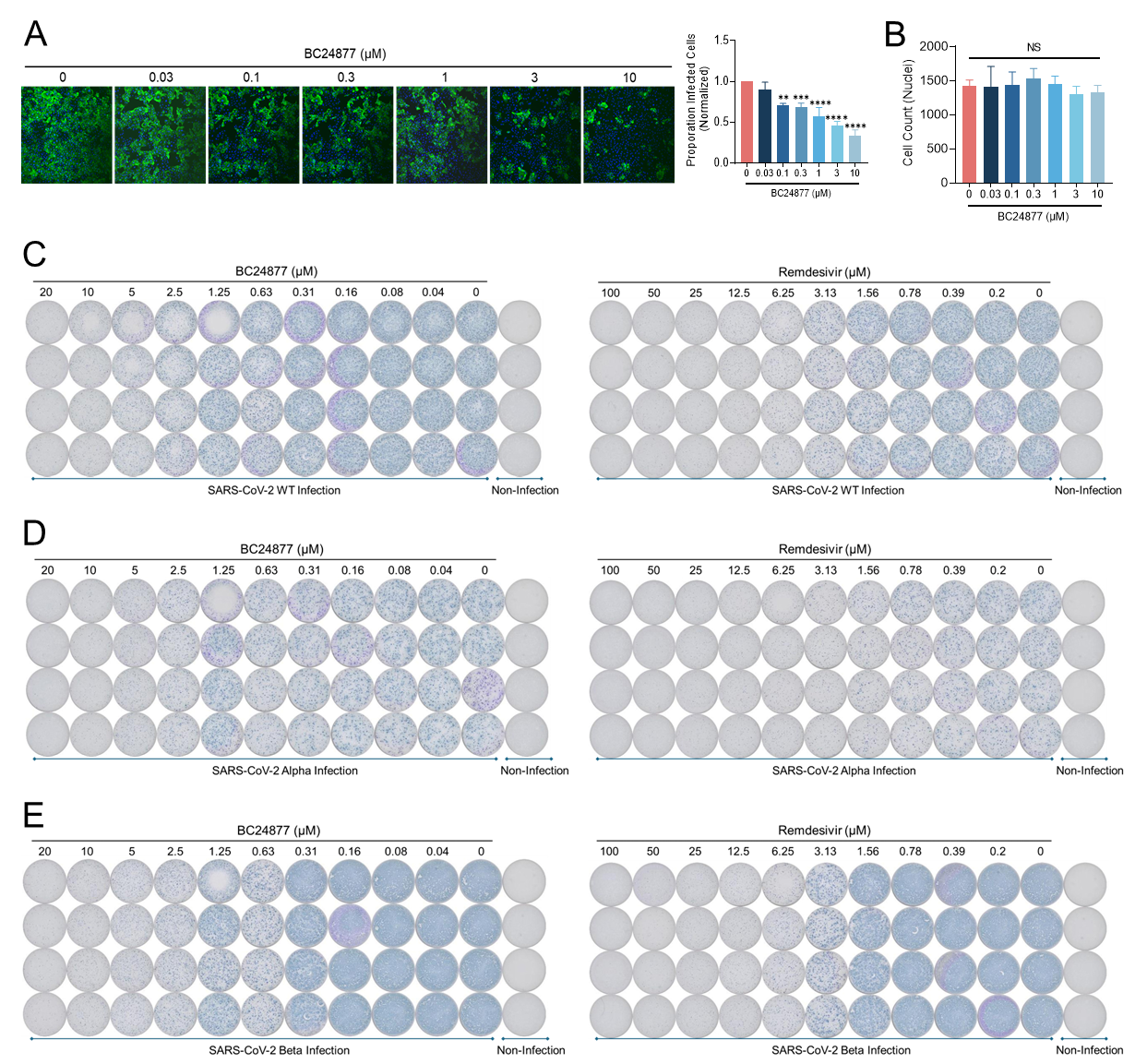
**Supplementary Figure S13 related to Figure 6.** **FBXO5 stabilizer suppresses coronavirus infection in VERO cells.** (A) Immunofluorescence staining of OC43 in VERO cells treated with BC24877 at the indicated dose for 24 h, data represent mean ±SEM (n=3). (B) Cell viability of VERO cells treated with BC24877 at the indicated dose, data represent mean ±SEM (n=3). (C) Immunospot staining of SARS-CoV2 WT, (D) Alpha, and (E) Beta in VERO cells treated with BC24877 or Remdesivir at indicated dose for 24 h. ***p* <0.01, ****p* <0.001, *****p* <0.0001 by one-way ANOVA with Dunnett’s multiple comparisons (A and B). NS, not significant.


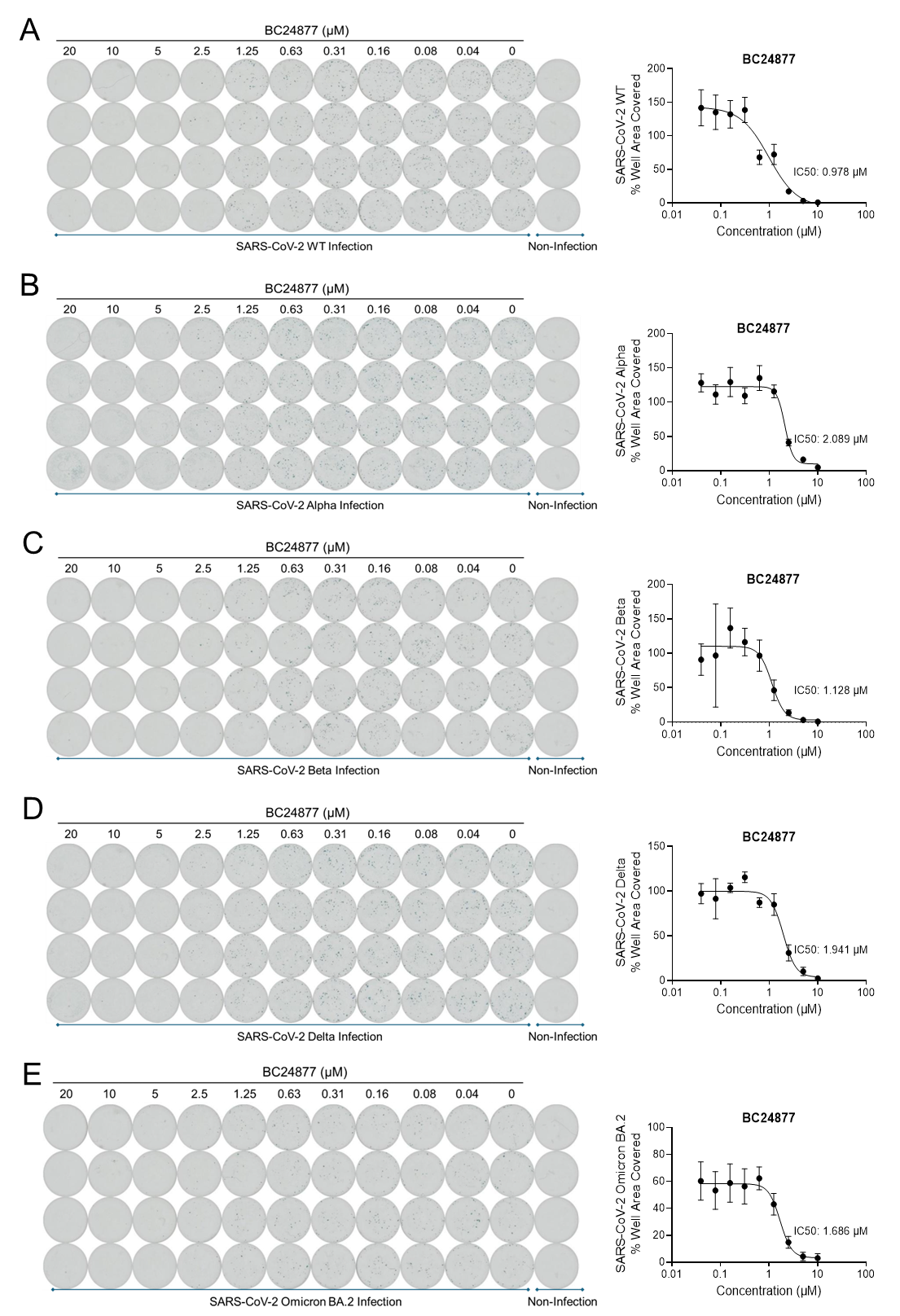


**Supplementary Figure S14 related to Figure 6. FBXO5 stabilizer suppresses coronavirus infection in Huh-7 cells.** (A) Immunospot staining of SARS-CoV2 WT, (B) Alpha, (C) Beta, (D) Delta, and (E) Omicron BA.2 in Huh-7 cells treated with or without BC24877 at the indicated dose for 24 h, data represent mean ±SEM (n=4).

**Description for Synthesis of BC24877 and Analogues**

*Reagents and conditions:* (*i*) *i*-PrOH, C_2_H_5_Br, reflux, overnight; (*ii*) MeOH, Et_3_N, Br_2_, 0°C-RT, 2h; (*iii*) Py, 50°C, overnight.

**Step 1. Synthesis of compound 2**

To a solution of thiourea **1** (5.0g, 65.7 mmol) in 2-propanol (100 mL) was added bromoethane (14.3g, 131mmol), and the reaction mixture was reflux overnight. The mixture was cooled and the formed precipitate was filtered off and dried giving the product **2** (carboxamidinoethyl sulfide hydrobromide) that was used in next step without purification. Yield 12.0g (95%).

^1^H NMR (400 MHz, DMSO-*d*_6_) δ 9.05 (br s, 2H), 8.95 (br s, 1H), 3.16 (dd, *J* = 7.2 Hz, *J* = 21.6Hz, 2H), 1.29 (t, *J* = 7.2 Hz, 3H).

MS (ESI)^+^ *m/z*: 105 [M + H]^+^.

**Step 2. Synthesis of compound 3**

Sodium thiocyanate (6.82 g, 70.2 mmol) was dissolved in methanol (50 mL), then carboxamidinoethyl sulfide **2** (5.0g, 27 mmol) and triethylamine (4.5 mL, 3.24g, 32 mmol) were added with stirring at 0°C. To the mixture, triethylamine was added dropwise (4.5mL, 3.24g, 32 mmol) simultaneously with addition of neat bromine (8.63 g, 2.69 mL, 54 mmol) upon stirring at 0°C. After 2h of stirring at room temperature, the reaction mixture was concentrated, and the crude product was purified by silica gel column chromatography (ethyl acetate/petroleum ether (v/v)=0-30%) to obtain compound 3-(ethylthio)-1,2,4-thiadiazol-5-amine **3** as a yellow solid (3.5 g, 80%).

^1^H NMR (400 MHz, DMSO-*d*_6_) δ 7.98 (br s, 2H), 3.05 (dd, *J* = 7.2 Hz, *J* = 22Hz, 2H), 1.29 (t, *J* = 7.2 Hz, 3H).

MS (ESI)^+^ *m/z*: 162 [M + H]^+^.

**Step 3. Synthesis of compounds 4.**

**General procedure**

5 mmol of amine **3** and 5 mmol of corresponding isocyanate were dissolved in 2 mL pyridine and stirred under N_2_ at 50°C for 24h. The reaction was cooled down to ambient temperature, the solvent was removed, and the residue was purified using column chromatography or HPLC to obtain the final products **4**, average yield 40-60%.

**BC24840**

^1^H NMR (400 MHz, DMSO-*d*_6_) δ 11.72 (s, 1H), 8.83 (s, 1H), 8.13 – 8.01 (m, 1H), 7.17 – 7.01 (m, 2H), 7.01 – 6.84 (m, 1H), 3.89 (s, 3H), 3.15 (q, *J* = 7.3 Hz, 2H), 1.34 (t, *J* = 7.3 Hz, 3H).

MS (ESI)^+^ *m/z*: 311 [M + H]^+^.

**BC24841**

^1^H NMR (400 MHz, DMSO-*d*_6_) δ 11.37 (s, 1H), 9.22 (s, 1H), 7.24 (t, *J* = 8.2 Hz, 1H), 7.16 (t, *J* = 2.2 Hz, 1H), 7.07 – 6.96 (m, 1H), 6.72 – 6.61 (m, 1H), 3.75 (s, 3H), 3.14 (q, *J* = 7.3 Hz, 2H), 1.34 (t, *J* = 7.3 Hz, 3H).

MS (ESI)^+^ *m/z*: 311 [M + H]^+^.

**BC24842**

^1^H NMR (400 MHz, DMSO) δ 11.55 (s, 1H), 9.43 (s, 1H), 7.69 (s, 1H), 7.39 – 7.35 (m, 2H), 7.17 – 7.12 (m, 1H), 3.15 (q, *J* = 7.3 Hz, 2H), 1.34 (t, *J* = 7.3 Hz, 3H).

MS (ESI)^+^ *m/z*: 316 [M + H]^+^.

**BC24843**

^1^H NMR (400 MHz, DMSO-*d*_6_) δ 11.91 (s, 1H), 8.86 (s, 1H), 8.09 (dd, *J* = 8.3, 1.5 Hz, 1H), 7.52 (dd, *J* = 8.1, 1.5 Hz, 1H), 7.42 – 7.29 (m, 1H), 7.16 (td, *J* = 7.7, 1.6 Hz, 1H), 3.15 (q, *J* = 7.3 Hz, 2H), 1.34 (t, *J* = 7.3 Hz, 3H).

MS (ESI)^+^ *m/z*: 316 [M + H]^+^.

**BC24844**

^1^H NMR (400 MHz, DMSO-*d*_6_) δ 11.38 (s, 1H), 9.22 (s, 1H), 7.49 (d, *J* = 7.2 Hz, 2H), 7.41 – 7.27 (m, 2H), 7.15 – 7.02 (m, 1H), 3.15 (q, *J* = 7.3 Hz, 2H), 1.34 (t, *J* = 7.3 Hz, 3H).

MS (ESI)^+^ *m/z*: 281 [M + H]^+^.

**BC24846**

^1^H NMR (400 MHz, DMSO-*d*_6_) δ 12.61 (s, 1H), 10.01 (s, 1H), 8.26 (dd, *J* = 8.5, 1.3 Hz, 1H), 8.15 (dd, *J* = 8.4, 1.5 Hz, 1H), 7.77 (ddd, *J* = 8.6, 7.2, 1.6 Hz, 1H), 7.34 (ddd, *J* = 8.5, 7.2, 1.3 Hz, 1H), 3.16 (q, *J* = 7.3 Hz, 2H), 1.35 (t, *J* = 7.3 Hz, 3H).

MS (ESI)^+^ *m/z*: 326 [M + H]^+^.

**BC24847**

^1^H NMR (400 MHz, DMSO-*d*_6_) δ 11.54 (s, 1H), 8.55 (s, 1H), 7.55 – 7.40 (m, 1H), 7.15 – 7.05 (m, 1H), 7.00 (d, *J* = 7.5 Hz, 1H), 3.14 (q, *J* = 7.3 Hz, 2H), 2.27 (s, 3H), 2.12 (s, 3H), 1.34 (t, *J* = 7.3 Hz, 3H).

MS (ESI)^+^ *m/z*: 309 [M + H]^+^.

**BC24848**

^1^H NMR (400 MHz, DMSO-*d*_6_) δ 11.31 (s, 1H), 9.02 (s, 1H), 7.27 (d, *J* = 2.3 Hz, 1H), 7.19 (dd, *J* = 8.1, 2.3 Hz, 1H), 7.08 (d, *J* = 8.2 Hz, 1H), 3.14 (q, *J* = 7.3 Hz, 2H), 2.21 (s, 3H), 2.17 (s, 3H), 1.34 (t, *J* = 7.3 Hz, 3H).

MS (ESI)^+^ *m/z*: 309 [M + H]^+^.

**BC24849**

^1^H NMR (400 MHz, DMSO-*d*_6_) δ 11.58 (s, 1H), 8.43 (s, 1H), 7.60 (d, *J* = 6.5 Hz, 1H), 7.10 (d, *J* = 7.6 Hz, 1H), 6.88 (d, *J* = 7.6 Hz, 1H), 3.15 (q, *J* = 7.3 Hz, 2H), 2.27 (s, 3H), 2.19 (s, 3H), 1.34 (t, *J* = 7.3 Hz, 3H).

MS (ESI)^+^ *m/z*: 309 [M + H]^+^.

**BC24850**

^1^H NMR (400 MHz, DMSO-*d*_6_) δ 11.71 (s, 1H), 9.64 (s, 1H), 7.25 (d, *J* = 7.2 Hz, 2H), 7.01 – 6.87 (m, 1H), 3.15 (q, *J* = 7.3 Hz, 2H), 1.34 (t, *J* = 7.3 Hz, 3H).

MS (ESI)^+^ *m/z*: 317 [M + H]^+^.

**BC24851**

^1^H NMR (400 MHz, DMSO-*d*_6_) δ 11.84 (s, 1H), 8.79 (s, 1H), 7.99 – 7.86 (m, 1H), 7.35 (d, *J* = 2.0 Hz, 1H), 7.17 (dd, *J* = 8.6, 2.0 Hz, 1H), 3.15 (q, *J* = 7.3 Hz, 2H), 2.28 (s, 3H), 1.34 (t, *J* = 7.3 Hz, 3H).

MS (ESI)^+^ *m/z*: 329 [M + H]^+^.

**BC24852**

^1^H NMR (400 MHz, DMSO-*d*_6_) δ 11.82 (s, 1H), 9.74 (s, 1H), 8.09 (d, *J* = 2.6 Hz, 1H), 7.77 (dd, *J* = 8.8, 2.5 Hz, 1H), 7.68 (d, *J* = 8.8 Hz, 1H), 3.15 (q, *J* = 7.3 Hz, 2H), 1.34 (t, *J* = 7.3 Hz, 3H).

MS (ESI)^+^ *m/z*: 384 [M + H]^+^.

**BC24853**

^1^H NMR (400 MHz, DMSO-*d*_6_) δ 11.75 (s, 1H), 8.85 (s, 1H), 7.75 (d, *J* = 3.0 Hz, 1H), 6.99 (d, *J* = 8.9 Hz, 1H), 6.61 (dd, *J* = 8.9, 3.0 Hz, 1H), 3.83 (s, 3H), 3.71 (s, 3H), 3.15 (q, *J* = 7.3 Hz, 2H), 1.34 (t, *J* = 7.3 Hz, 3H).

MS (ESI)^+^ *m/z*: 311 [M + H]^+^.

**BC24854**

^1^H NMR (400 MHz, DMSO-*d*_6_) δ 11.34 (s, 1H), 9.12 (s, 1H), 7.37 (d, *J* = 8.3 Hz, 2H), 7.14 (d, *J* = 8.3 Hz, 2H), 3.14 (q, *J* = 7.3 Hz, 2H), 2.26 (s, 3H), 1.34 (t, *J* = 7.3 Hz, 3H).

MS (ESI)^+^ *m/z*: 341 [M + H]^+^.

**BC24855**

^1^H NMR (400 MHz, DMSO-*d*_6_) δ 11.61 (s, 1H), 8.50 (s, 1H), 7.81 – 7.69 (m, 1H), 7.29 – 7.13 (m, 2H), 7.06 (td, *J* = 7.4, 1.3 Hz, 1H), 3.14 (q, *J* = 7.3 Hz, 2H), 2.24 (s, 3H), 1.34 (t, *J* = 7.3 Hz, 3H).

MS (ESI)^+^ *m/z*: 295 [M + H]^+^.

**BC24856**

^1^H NMR (400 MHz, DMSO-*d*_6_) δ 11.65 (s, 1H), 9.44 (s, 1H), 7.79 (dd, *J* = 6.7, 2.4 Hz, 1H), 7.48 – 7.33 (m, 2H), 3.15 (q, *J* = 7.3 Hz, 2H), 1.34 (t, *J* = 7.3 Hz, 3H).

MS (ESI)^+^ *m/z*: 334 [M + H]^+^.

**BC24857**

^1^H NMR (400 MHz, DMSO-*d*_6_) δ 11.35 (s, 1H), 9.05 (s, 1H), 7.39 (d, *J* = 8.9 Hz, 2H), 6.91 (d, *J* = 8.9 Hz, 2H), 3.73 (s, 3H), 3.14 (q, *J* = 7.3 Hz, 2H), 1.34 (t, *J* = 7.3 Hz, 3H).

MS (ESI)^+^ *m/z*: 311 [M + H]^+^.

**BC24858**

^1^H NMR (400 MHz, DMSO) δ 12.00 (s, 1H), 8.65 (s, 1H), 8.07 (d, *J* = 8.1 Hz, 1H), 7.11 – 6.98 (m, 2H), 6.92 (dd, *J* = 16.2, 9.6 Hz, 1H), 4.13 (q, *J* = 6.9 Hz, 2H), 3.15 (q, *J* = 7.3 Hz, 2H), 1.41 (t, *J* = 7.0 Hz, 3H), 1.34 (t, *J* = 7.3 Hz, 3H).

MS (ESI)^+^ *m/z*: 325 [M + H]^+^.

**BC24859**

^1^H NMR (400 MHz, DMSO-*d*_6_) δ 11.34 (s, 1H), 9.03 (s, 1H), 7.37 (d, *J* = 9.0 Hz, 2H), 6.89 (d, *J* = 9.0 Hz, 2H), 3.99 (q, *J* = 7.0 Hz, 2H), 3.14 (q, *J* = 7.3 Hz, 2H), 1.41 – 1.23 (m, 6H).

MS (ESI)^+^ *m/z*: 325 [M + H]^+^.

**BC24860**

^1^H NMR (400 MHz, DMSO) δ 11.56 (s, 1H), 9.42 (s, 1H), 7.83 (s, 1H), 7.42 (d, *J* = 7.3 Hz, 1H), 7.35 – 7.21 (m, 2H), 3.15 (q, *J* = 7.2 Hz, 2H), 1.34 (t, *J* = 7.3 Hz, 3H).

MS (ESI)^+^ *m/z*: 360 [M + H]^+^.

**BC24862**

^1^H NMR (400 MHz, DMSO-*d*_6_) δ 11.68 (s, 1H), 8.75 (s, 1H), 7.66 (dd, *J* = 7.8, 1.5 Hz, 1H), 7.33 – 7.16 (m, 2H), 3.23 – 3.06 (m, 2H), 2.28 (s, 3H), 1.34 (t, *J* = 7.3 Hz, 3H).

MS (ESI)^+^ *m/z*: 329 [M + H]^+^.

**BC24863**

^1^H NMR (400 MHz, DMSO-*d*_6_) δ 11.35 (s, 1H), 9.12 (s, 1H), 7.34 (s, 1H), 7.31 – 7.11 (m, 2H), 6.90 (d, *J* = 7.2 Hz, 1H), 3.15 (q, *J* = 7.3 Hz, 2H), 2.30 (s, 3H), 1.34 (t, *J* = 7.3 Hz, 3H).

MS (ESI)^+^ *m/z*: 295 [M + H]^+^.

**BC24864**

^1^H NMR (400 MHz, DMSO-*d*_6_) δ 11.61 (s, 1H), 8.49 (s, 1H), 7.72 (d, *J* = 6.6 Hz, 1H), 7.31 – 7.17 (m, 2H), 7.13 (dd, *J* = 7.5, 1.4 Hz, 1H), 3.15 (q, *J* = 7.3 Hz, 2H), 2.60 (q, *J* = 7.5 Hz, 2H), 1.34 (t, *J* = 7.3 Hz, 3H), 1.16 (t, *J* = 7.5 Hz, 3H).

MS (ESI)^+^ *m/z*: 309 [M + H]^+^.

**BC24865**

^1^H NMR (400 MHz, DMSO-*d*_6_) δ 11.38 (s, 1H), 9.21 (s, 1H), 7.43 (d, *J* = 8.5 Hz, 2H), 7.23 (d, *J* = 8.6 Hz, 2H), 4.07 (q, *J* = 7.1 Hz, 2H), 3.62 (s, 2H), 3.14 (q, *J* = 7.3 Hz, 2H), 1.34 (t, *J* = 7.3 Hz, 3H), 1.18 (t, *J* = 7.1 Hz, 3H).

MS (ESI)^+^ *m/z*: 367 [M + H]^+^.

**BC24866**

^1^H NMR (400 MHz, DMSO-*d*_6_) δ 11.92 (s, 1H), 8.68 (s, 1H), 7.89 (d, *J* = 8.0 Hz, 1H), 7.84 – 7.58 (m, 2H), 7.42 (t, *J* = 7.7 Hz, 1H), 3.15 (q, *J* = 7.3 Hz, 2H), 1.34 (t, *J* = 7.3 Hz, 3H).

MS (ESI)^+^ *m/z*: 349 [M + H]^+^.

**BC24867**

^1^H NMR (400 MHz, DMSO-*d*_6_) δ 11.55 (s, 1H), 8.43 (s, 1H), 7.57 (d, *J* = 7.8 Hz, 1H), 7.09 – 6.95 (m, 2H), 3.14 (q, *J* = 7.3 Hz, 2H), 2.25 (s, 3H), 2.20 (s, 3H), 1.34 (t, *J* = 7.3 Hz, 3H).

MS (ESI)^+^ *m/z*: 309 [M + H]^+^.

**BC24868**

^1^H NMR (400 MHz, DMSO-*d*_6_) δ 11.69 (s, 1H), 7.16 (s, 1H), 4.12 (q, *J* = 7.1 Hz, 2H), 3.94 (d, *J* = 5.9 Hz, 2H), 3.12 (q, *J* = 7.3 Hz, 2H), 1.33 (t, *J* = 7.3 Hz, 3H), 1.20 (t, *J* = 7.1 Hz, 3H).

MS (ESI)^+^ *m/z*: 291 [M + H]^+^.

**BC24869**

^1^H NMR (400 MHz, DMSO) δ 11.34 (s, 1H), 6.83 (s, 1H), 3.34 (dd, *J* = 11.3, 5.2 Hz, 2H), 3.26 – 3.17 (m, 5H), 3.11 (q, *J* = 7.3 Hz, 2H), 1.69 (p, *J* = 6.6 Hz, 2H), 1.32 (t, *J* = 7.3 Hz, 3H).

MS (ESI)^+^ *m/z*: 277 [M + H]^+^.

**BC24870**

^1^H NMR (400 MHz, DMSO-*d*_6_) δ 11.46 (s, 1H), 9.27 (s, 1H), 7.51 (dd, *J* = 9.0, 4.9 Hz, 2H), 7.18 (t, *J* = 8.8 Hz, 2H), 3.14 (q, *J* = 7.3 Hz, 2H), 1.34 (t, *J* = 7.3 Hz, 3H).

MS (ESI)^+^ *m/z*: 299 [M + H]^+^.

**BC24871**

^1^H NMR (400 MHz, DMSO-*d*_6_) δ 11.32 (s, 1H), 7.56 (d, *J* = 8.4 Hz, 1H), 7.44 – 7.21 (m, 5H), 5.17 (d, *J* = 7.5 Hz, 1H), 4.09 – 3.89 (m, 2H), 3.11 (q, *J* = 7.3 Hz, 2H), 3.02 – 2.81 (m, 2H), 1.31 (t, *J* = 7.3 Hz, 3H), 1.09 (t, *J* = 7.1 Hz, 3H).

MS (ESI)^+^ *m/z*: 381 [M + H]^+^.

**BC24872**

^1^H NMR (400 MHz, DMSO) δ 10.94 (s, 1H), 6.83 (s, 1H), 3.96 (dd, *J* = 13.4, 6.7 Hz, 1H), 3.11 (q, *J* = 7.3 Hz, 2H), 1.92 – 1.81 (m, 2H), 1.71 – 1.62 (m, 2H), 1.61 – 1.51 (m, 2H), 1.47 – 1.36 (m, 2H), 1.32 (t, *J* = 7.3 Hz, 3H).

MS (ESI)^+^ *m/z*: 273 [M + H]^+^.

**BC24873**

^1^H NMR (400 MHz, DMSO) δ 11.46 (s, 1H), 9.33 (s, 1H), 8.77 (s, 1H), 7.23 (d, *J* = 8.6 Hz, 1H), 7.01 (d, *J* = 7.5 Hz, 1H), 3.13 (q, *J* = 7.3 Hz, 2H), 2.18 (s, 3H), 1.34 (t, *J* = 7.3 Hz, 3H).

MS (ESI)^+^ *m/z*: 313 [M + H]^+^.

**BC24874**

^1^H NMR (400 MHz, DMSO-*d*_6_) δ 11.37 (s, 1H), 6.98 (s, 1H), 3.61 (s, 3H), 3.49 – 3.34 (m, 2H), 3.11 (q, *J* = 7.3 Hz, 2H), 2.55 (t, *J* = 6.6 Hz, 2H), 1.32 (t, *J* = 7.3 Hz, 3H).

MS (ESI)^+^ *m/z*: 291 [M + H]^+^.

**BC24875**

^1^H NMR (400 MHz, DMSO) δ 11.34 (s, 1H), 6.81 (s, 1H), 3.45 – 3.34 (m, 4H), 3.21 (dd, *J* = 12.6, 6.3 Hz, 2H), 3.11 (q, *J* = 7.3 Hz, 2H), 1.75 – 1.59 (m, 2H), 1.32 (t, *J* = 7.3 Hz, 3H), 1.10 (t, *J* = 7.0 Hz, 3H).

MS (ESI)^+^ *m/z*: 291 [M + H]^+^.

**BC24876**

^1^H NMR (400 MHz, DMSO-*d*_6_) δ 11.51 (s, 1H), 9.27 (s, 1H), 8.03 – 7.85 (m, 1H), 7.44 – 7.26 (m, 1H), 7.04 – 6.91 (m, 1H), 3.15 (q, *J* = 7.3 Hz, 2H), 1.34 (t, *J* = 7.3 Hz, 3H).

MS (ESI)^+^ *m/z*: 317 [M + H]^+^.

**BC24877**

^1^H NMR (400 MHz, DMSO-*d*_6_) δ 11.67 (s, 1H), 9.60 (s, 1H), 7.99 (s, 1H), 7.76 – 7.64 (m, 1H), 7.58 (t, *J* = 8.0 Hz, 1H), 7.44 (d, *J* = 7.7 Hz, 1H), 3.15 (q, *J* = 7.3 Hz, 2H), 1.35 (t, *J* = 7.3 Hz, 3H).

MS (ESI)^+^ *m/z*: 349 [M + H]^+^.

**BC24878**

^1^H NMR (400 MHz, DMSO-*d*_6_) δ 11.48 (s, 1H), 9.28 (s, 1H), 7.57 – 7.45 (m, 2H), 7.28 (dd, *J* = 8.7, 2.7 Hz, 1H), 3.14 (q, *J* = 7.3 Hz, 2H), 2.33 (s, 3H), 1.34 (t, *J* = 7.3 Hz, 3H).

MS (ESI)^+^ *m/z*: 374 [M + H]^+^.

**BC24879**

^1^H NMR (400 MHz, DMSO-*d*_6_) δ 11.46 (s, 1H), 7.35 (s, 1H), 7.23 – 7.09 (m, 4H), 4.30 (d, *J* = 4.9 Hz, 2H), 3.11 (q, *J* = 7.3 Hz, 2H), 2.27 (s, 3H), 1.32 (t, *J* = 7.3 Hz, 3H).

MS (ESI)^+^ *m/z*: 309 [M + H]^+^.

**BC24880**

^1^H NMR (400 MHz, DMSO-*d*_6_) δ 11.32 (s, 1H), 9.04 (s, 1H), 7.06 (d, *J* = 2.3 Hz, 1H), 6.87 (dd, *J* = 8.7, 2.5 Hz, 1H), 6.81 (d, *J* = 8.7 Hz, 1H), 4.22 (d, *J* = 4.2 Hz, 4H), 3.14 (q, *J* = 7.3 Hz, 2H), 1.34 (t, *J* = 7.3 Hz, 3H).

MS (ESI)^+^ *m/z*: 339 [M + H]^+^.

**BC24881**

^1^H NMR (400 MHz, DMSO-*d*_6_) δ 11.29 (s, 1H), 7.37 – 7.15 (m, 5H), 6.84 (s, 1H), 3.46 – 3.35 (m, 2H), 3.11 (q, *J* = 7.3 Hz, 2H), 2.78 (t, *J* = 7.3 Hz, 2H), 1.32 (t, *J* = 7.3 Hz, 3H).

MS (ESI)^+^ *m/z*: 309 [M + H]^+^.

**BC24883**

^1^H NMR (400 MHz, DMSO-*d*_6_) δ 11.49 (s, 1H), 7.39 (s, 1H), 7.25 (t, *J* = 8.1 Hz, 1H), 6.92 – 6.72 (m, 3H), 4.32 (d, *J* = 5.6 Hz, 2H), 3.73 (s, 3H), 3.12 (q, *J* = 7.3 Hz, 2H), 1.32 (t, *J* = 7.3 Hz, 3H).

MS (ESI)^+^ *m/z*: 325 [M + H]^+^.

**BC24885**

^1^H NMR (400 MHz, DMSO-*d*_6_) δ 11.39 (s, 1H), 7.59 (d, *J* = 1.9 Hz, 1H), 7.31 (s, 1H), 6.40 (dd, *J* = 3.2, 1.9 Hz, 1H), 6.29 (d, *J* = 3.2 Hz, 1H), 4.35 (d, *J* = 5.8 Hz, 2H), 3.12 (q, *J* = 7.3 Hz, 2H), 1.32 (t, *J* = 7.3 Hz, 3H).

MS (ESI)^+^ *m/z*: 285 [M + H]^+^.

**BC24886**

^1^H NMR (400 MHz, DMSO-*d*_6_) δ 11.51 (s, 1H), 7.47 – 7.25 (m, 3H), 7.16 (t, *J* = 8.9 Hz, 2H), 4.33 (d, *J* = 5.7 Hz, 2H), 3.12 (q, *J* = 7.3 Hz, 2H), 1.32 (t, *J* = 7.3 Hz, 3H).

MS (ESI)^+^ *m/z*: 313 [M + H]^+^.

**BC24887**

^1^H NMR (400 MHz, DMSO-*d*_6_) δ 11.19 (s, 1H), 6.88 (s, 1H), 3.96 – 3.84 (m, 1H), 3.77 (dt, *J* = 8.1, 6.5 Hz, 1H), 3.70 – 3.57 (m, 1H), 3.40 – 3.23 (m, 1H), 3.23 – 3.04 (m, 3H), 1.98 – 1.73 (m, 3H), 1.58 – 1.42 (m, 1H), 1.32 (t, *J* = 7.3 Hz, 3H).

MS (ESI)^+^ *m/z*: 289 [M + H]^+^.

**BC24888**

^1^H NMR (400 MHz, DMSO-*d*_6_) δ 11.44 (s, 1H), 9.27 (s, 1H), 7.45 (s, 1H), 7.33 – 7.16 (m, 2H), 6.98 (dt, *J* = 7.7, 1.5 Hz, 1H), 3.15 (q, *J* = 7.3 Hz, 2H), 2.47 (s, 3H), 1.34 (t, *J* = 7.3 Hz, 3H).

MS (ESI)^+^ *m/z*: 327 [M + H]^+^.

**BC24889**

^1^H NMR (400 MHz, DMSO-*d*_6_) δ 11.37 (s, 1H), 7.32 (d, *J* = 7.3 Hz, 1H), 4.41 – 4.21 (m, 1H), 3.67 (s, 3H), 3.12 (q, *J* = 7.3 Hz, 2H), 1.42 – 1.26 (m, 6H).

MS (ESI)^+^ *m/z*: 291 [M + H]^+^.

**BC24891**

^1^H NMR (400 MHz, DMSO) δ 11.03 (s, 1H), 6.70 (s, 1H), 3.79 (dt, *J* = 13.5, 6.8 Hz, 1H), 3.11 (q, *J* = 7.3 Hz, 2H), 1.32 (t, *J* = 7.3 Hz, 3H), 1.13 (d, *J* = 6.5 Hz, 6H).

MS (ESI)^+^ *m/z*: 247 [M + H]^+^.

**BC24892**

^1^H NMR (400 MHz, DMSO) δ 11.00 (s, 1H), 6.65 (s, 1H), 3.63 (dt, *J* = 13.5, 6.6 Hz, 1H), 3.11 (q, *J* = 7.3 Hz, 2H), 1.45 (p, *J* = 7.3 Hz, 2H), 1.32 (t, *J* = 7.3 Hz, 3H), 1.10 (d, *J* = 6.6 Hz, 3H), 0.85 (t, *J* = 7.4 Hz, 3H).

MS (ESI)^+^ *m/z*: 261 [M + H]^+^.

**BC24893**

^1^H NMR (400 MHz, DMSO-*d*_6_) δ 11.26 (s, 1H), 6.82 (s, 1H), 3.22 – 3.03 (m, 4H), 1.56 (dq, *J* = 13.3, 6.7 Hz, 1H), 1.42 – 1.24 (m, 5H), 0.88 (d, *J* = 6.6 Hz, 6H).

MS (ESI)^+^ *m/z*: 275 [M + H]^+^.

**BC24894**

^1^H NMR (400 MHz, DMSO) δ 10.97 (s, 1H), 6.73 (s, 1H), 3.49 (dd, *J* = 12.4, 6.7 Hz, 1H), 3.11 (q, *J* = 7.3 Hz, 2H), 1.83 – 1.74 (m, 2H), 1.68 – 1.59 (m, 2H), 1.36 – 1.16 (m, 9H).

MS (ESI)^+^ *m/z*: 287 [M + H]^+^.

**BC24895**

^1^H NMR (400 MHz, DMSO) δ 11.55 (s, 1H), 9.43 (s, 1H), 7.71 (s, 1H), 7.42 – 7.31 (m, 3H), 3.13 (q, *J* = 7.3 Hz, 2H), 1.33 (t, *J* = 7.3 Hz, 3H).

MS (ESI)^+^ *m/z*: 299 [M + H]^+^.

**BC24896**

^1^H NMR (400 MHz, DMSO-*d*_6_) δ 11.18 (s, 1H), 6.86 (s, 1H), 3.11 (q, *J* = 7.3 Hz, 2H), 3.04 – 2.90 (m, 2H), 1.73 (dt, *J* = 13.4, 6.7 Hz, 1H), 1.32 (t, *J* = 7.3 Hz, 3H), 0.86 (d, *J* = 6.7 Hz, 6H).

MS (ESI)^+^ *m/z*: 261 [M + H]^+^.

**BC24897**

^1^H NMR (400 MHz, DMSO-*d*_6_) δ 11.38 (s, 1H), 6.96 (s, 1H), 4.07 (q, *J* = 7.1 Hz, 2H), 3.45 – 3.35 (m, 2H), 3.11 (q, *J* = 7.3 Hz, 2H), 2.57 – 2.52 (m, 2H), 1.32 (t, *J* = 7.3 Hz, 3H), 1.18 (t, *J* = 7.1 Hz, 3H).

MS (ESI)^+^ *m/z*: 305 [M + H]^+^.

**BC24899**

^1^H NMR (400 MHz, DMSO) δ 11.23 (s, 1H), 6.88 (s, 1H), 3.43 – 3.38 (m, 2H), 3.28 – 3.21 (m, 5H), 3.12 (d, *J* = 7.0 Hz, 2H), 1.32 (t, *J* = 6.8 Hz, 3H).

MS (ESI)^+^ *m/z*: 263 [M + H]^+^.

**BC24900**

^1^H NMR (400 MHz, DMSO) δ 10.78 (s, 1H), 6.62 (s, 1H), 3.11 (q, *J* = 7.3 Hz, 2H), 1.34 – 1.27 (m, 12H).

MS (ESI)^+^ *m/z*: 261 [M + H]^+^.

**BC24902**

^1^H NMR (400 MHz, DMSO-*d*_6_) δ 11.36 (s, 1H), 7.29 (d, *J* = 7.3 Hz, 1H), 4.37 – 4.21 (m, 1H), 4.21 – 4.01 (m, 2H), 3.12 (q, *J* = 7.3 Hz, 2H), 1.44 – 1.25 (m, 6H), 1.20 (t, *J* = 7.1 Hz, 3H).

MS (ESI)^+^ *m/z*: 305 [M + H]^+^.

**BC24904**

^1^H NMR (400 MHz, DMSO-*d*_6_) δ 11.58 (s, 1H), 7.47 (s, 1H), 7.42 – 7.30 (m, 1H), 7.22 – 7.01 (m, 3H), 4.36 (d, *J* = 2.3 Hz, 2H), 3.12 (q, *J* = 7.3 Hz, 2H), 1.32 (t, *J* = 7.3 Hz, 3H).

MS (ESI)^+^ *m/z*: 313 [M + H]^+^.

**BC24905**

^1^H NMR (400 MHz, DMSO-*d*_6_) δ 11.64 (s, 1H), 8.40 (s, 1H), 7.20 – 7.05 (m, 3H), 3.13 (q, *J* = 7.3 Hz, 2H), 2.17 (s, 6H), 1.33 (t, *J* = 7.3 Hz, 3H).

MS (ESI)^+^ *m/z*: 309 [M + H]^+^.

**BC24906**

^1^H NMR (400 MHz, DMSO) δ 11.39 (s, 1H), 7.27 (s, 1H), 7.22 (d, *J* = 8.6 Hz, 2H), 6.89 (d, *J* = 8.7 Hz, 2H), 4.27 (d, *J* = 5.8 Hz, 2H), 3.73 (s, 3H), 3.12 (q, *J* = 7.3 Hz, 2H), 1.32 (t, *J* = 7.3 Hz, 3H).

MS (ESI)^+^ *m/z*: 325 [M + H]^+^.

**BC24907**

^1^H NMR (400 MHz, DMSO-*d*_6_) δ 11.47 (s, 1H), 9.33 (s, 1H), 7.40 (dd, *J* = 12.1, 2.1 Hz, 1H), 7.23 (t, *J* = 8.5 Hz, 1H), 7.14 (dd, *J* = 8.2, 2.2 Hz, 1H), 3.14 (q, *J* = 7.3 Hz, 2H), 2.18 (s, 3H), 1.34 (t, *J* = 7.3 Hz, 3H).

MS (ESI)^+^ *m/z*: 313 [M + H]^+^.

**BC24908**

^1^H NMR (400 MHz, DMSO-*d*_6_) δ 11.33 (s, 1H), 9.12 (s, 1H), 7.38 (d, *J* = 8.5 Hz, 2H), 7.15 (d, *J* = 8.5 Hz, 2H), 3.14 (q, *J* = 7.3 Hz, 2H), 2.60 – 2.38 (m, 2H), 1.61 – 1.42 (m, 2H), 1.41 – 1.17 (m, 5H), 0.89 (t, *J* = 7.3 Hz, 3H).

MS (ESI)^+^ *m/z*: 337 [M + H]^+^.

**BC24910**

^1^H NMR (400 MHz, DMSO-*d*_6_) δ 11.79 (s, 1H), 9.60 (s, 1H), 7.46 (dd, *J* = 10.3, 6.5 Hz, 2H), 3.15 (q, *J* = 7.3 Hz, 2H), 1.34 (t, *J* = 7.3 Hz, 3H).

MS (ESI)^+^ *m/z*: 335 [M + H]^+^.

**BC24911**

^1^H NMR (400 MHz, DMSO-*d*_6_) δ 11.59 (s, 1H), 9.25 (s, 1H), 8.05 – 7.92 (m, 1H), 7.39 – 7.29 (m, 1H), 7.29 – 7.17 (m, 1H), 3.15 (q, *J* = 7.3 Hz, 2H), 1.34 (t, *J* = 7.3 Hz, 3H).

MS (ESI)^+^ *m/z*: 333 [M + H]^+^.

**BC24912**

^1^H NMR (400 MHz, DMSO-*d*_6_) δ 11.59 (s, 1H), 9.65 (s, 1H), 7.79 – 7.64 (m, 4H), 3.15 (q, *J* = 7.3 Hz, 2H), 1.34 (t, *J* = 7.3 Hz, 3H).

MS (ESI)^+^ *m/z*: 349 [M + H]^+^.

**BC24913**

^1^H NMR (400 MHz, DMSO-*d*_6_) δ 11.22 (s, 1H), 6.90 (s, 1H), 4.11 – 3.96 (m, 2H), 3.18 – 3.06 (m, 2H), 3.06 – 2.89 (m, 2H), 2.28 – 2.12 (m, 1H), 1.89 (d, *J* = 12.9 Hz, 2H), 1.72 (d, *J* = 13.1 Hz, 2H), 1.53 – 1.22 (m, 6H), 1.16 (t, *J* = 7.2 Hz, 3H), 1.04 – 0.85 (m, 2H).

MS (ESI)^+^ *m/z*: 373[M + H]^+^.

**BC24915**

^1^H NMR (400 MHz, DMSO-*d*_6_) δ 11.31 (s, 1H), 9.08 (s, 1H), 7.40 (s, 1H), 7.25 – 7.08 (m, 2H), 3.14 (q, *J* = 7.3 Hz, 2H), 2.92 – 2.73 (m, 4H), 2.01 (p, *J* = 7.4 Hz, 2H), 1.34 (t, *J* = 7.3 Hz, 3H).

MS (ESI)^+^ *m/z*: 321[M + H]^+^.

**BC24916**

^1^H NMR (400 MHz, DMSO-*d*_6_) δ 11.52 (s, 1H), 8.40 (s, 1H), 7.47 (d, *J* = 8.7 Hz, 1H), 6.83 (d, *J* = 2.9 Hz, 1H), 6.81 – 6.71 (m, 1H), 3.73 (s, 3H), 3.14 (q, *J* = 7.3 Hz, 2H), 2.20 (s, 3H), 1.34 (t, *J* = 7.3 Hz, 3H).

MS (ESI)^+^ *m/z*: 325 [M + H]^+^.

**Supplementary Table 1.** **Synthesis and SAR Evaluation of Novel Compounds**

| **Compounds** | **R group** | **SMILES** | **IUPAC** | **NSP7- HiBiT** | **OC43** |
| --- | --- | --- | --- | --- | --- |
| **BC24840** | **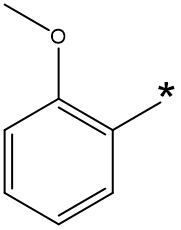** | S1N=C(SCC)N=C1NC(=O)NC1=C(OC)C=CC=C1 | 3-(ethylthio)-5-[3-(o-methoxyphenyl)ureido]-1,2,4-thiadiazole | **>100** | **11.47** |
| **BC24841** | **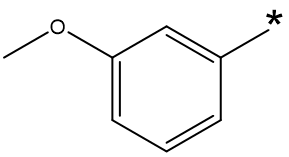** | S1N=C(SCC)N=C1NC(=O)NC1=CC(OC)=CC=C1 | 3-(ethylthio)-5-[3-(m-methoxyphenyl)ureido]-1,2,4-thiadiazole | **12.3** | **5.496** |
| **BC24842** | **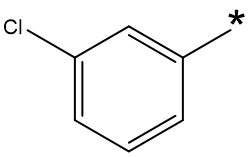** | ClC1=CC(NC(=O)NC2=NC(SCC)=NS2)=CC=C1 | 5-[3-(m-chlorophenyl)ureido]-3-(ethylthio)-1,2,4-thiadiazole | **2.153** | **1.565** |
| **BC24843** | **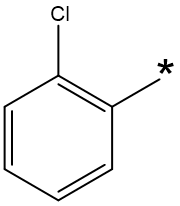** | ClC1=C(NC(=O)NC2=NC(SCC)=NS2)C=CC=C1 | 5-[3-(o-chlorophenyl)ureido]-3-(ethylthio)-1,2,4-thiadiazole | **26.23** | **16.2** |
| **BC24844** | **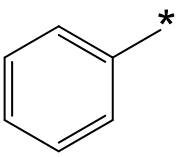** | S1N=C(SCC)N=C1NC(=O)NC1=CC=CC=C1 | 3-(ethylthio)-5-(3-phenylureido)-1,2,4-thiadiazole | **9.371** | **9.063** |
| **BC24846** | **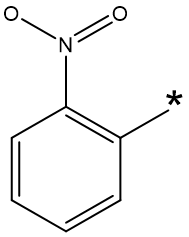** | S1N=C(SCC)N=C1NC(=O)NC1=C([N+](=O)[O-])C=CC=C1 | 3-(ethylthio)-5-[3-(o-nitrophenyl)ureido]-1,2,4-thiadiazole | **24.3** | **11.44** |
| **BC24847** | **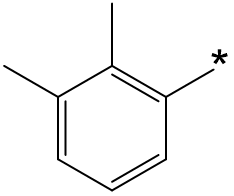** | S1N=C(SCC)N=C1NC(=O)NC1=C(C)C(C)=CC=C1 | 3-(ethylthio)-5-[3-(2,3-xylyl)ureido]-1,2,4-thiadiazole | **65.79** | **9.14** |
| **BC24848** | **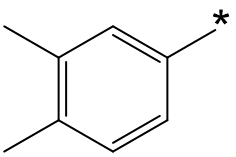** | S1N=C(SCC)N=C1NC(=O)NC1=CC(C)=C(C)C=C1 | 3-(ethylthio)-5-[3-(3,4-xylyl)ureido]-1,2,4-thiadiazole | **15.49** | **7.048** |
| **BC24849** | **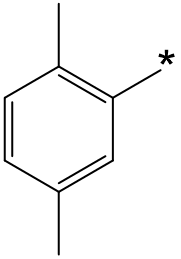** | S1N=C(SCC)N=C1NC(=O)NC1=C(C)C=CC(C)=C1 | 3-(ethylthio)-5-[3-(2,5-xylyl)ureido]-1,2,4-thiadiazole | **12.8** | **5.346** |
| **BC24850** | **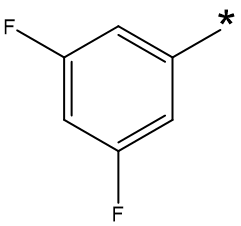** | S1N=C(SCC)N=C1NC(=O)NC1=CC(F)=CC(F)=C1 | 5-[3-(3,5-difluorophenyl)ureido]-3-(ethylthio)-1,2,4-thiadiazole | **2.955** | **1.218** |
| **BC24851** | **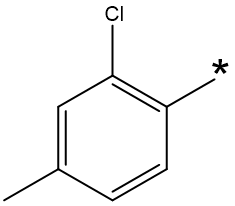** | ClC1=C(NC(=O)NC2=NC(SCC)=NS2)C=CC(C)=C1 | 5-[3-(2-chloro-4-tolyl)ureido]-3-(ethylthio)-1,2,4-thiadiazole | **>100** | **17.44** |
| **BC24852** | **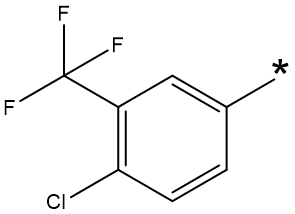** | ClC1=C(C(F)(F)F)C=C(NC(=O)NC2=NC(SCC)=NS2)C=C1 | 5-{3-[4-chloro-3-(trifluoromethyl)phenyl]ureido}-3-(ethylthio)-1,2,4-thiadiazole | **2.759** | **0.907** |
| **BC24853** | **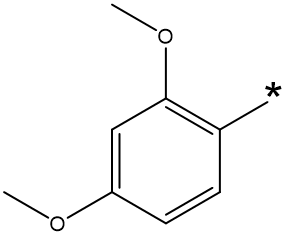** | S1N=C(SCC)N=C1NC(=O)NC1=C(OC)C=CC(OC)=C1 | 5-[3-(2,5-dimethoxyphenyl)ureido]-3-(ethylthio)-1,2,4-thiadiazole | **>100** | **9.873** |
| **BC24854** | **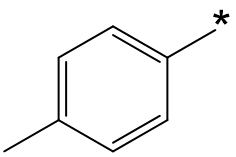** | S1N=C(SCC)N=C1NC(=O)NC1=CC=C(C)C=C1 | 3-(ethylthio)-5-[3-(p-tolyl)ureido]-1,2,4-thiadiazole | **45.15** | **26.63** |
| **BC24855** | **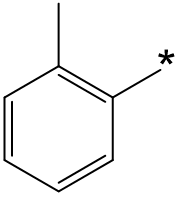** | S1N=C(SCC)N=C1NC(=O)NC1=C(C)C=CC=C1 | 3-(ethylthio)-5-[3-(o-tolyl)ureido]-1,2,4-thiadiazole | **35.21** | **8.227** |
| **BC24856** | **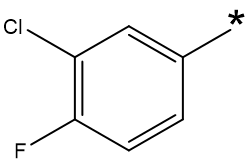** | ClC1=C(F)C=CC(NC(=O)NC2=NC(SCC)=NS2)=C1 | 5-[3-(3-chloro-4-fluorophenyl)ureido]-3-(ethylthio)-1,2,4-thiadiazole | **11.84** | **1.413** |
| **BC24857** | **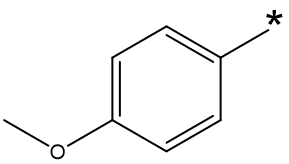** | S1N=C(SCC)N=C1NC(=O)NC1=CC=C(OC)C=C1 | 3-(ethylthio)-5-[3-(p-methoxyphenyl)ureido]-1,2,4-thiadiazole | **>100** | **88.78** |
| **BC24858** | **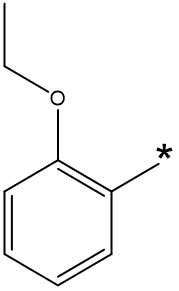** | S1N=C(SCC)N=C1NC(=O)NC1=C(OCC)C=CC=C1 | 5-[3-(o-ethoxyphenyl)ureido]-3-(ethylthio)-1,2,4-thiadiazole | **>100** | **24.33** |
| **BC24859** | **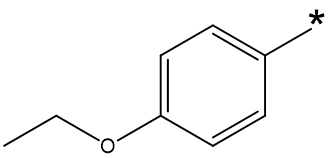** | S1N=C(SCC)N=C1NC(=O)NC1=CC=C(OCC)C=C1 | 5-[3-(p-ethoxyphenyl)ureido]-3-(ethylthio)-1,2,4-thiadiazole | **>100** | **52.12** |
| **BC24860** | **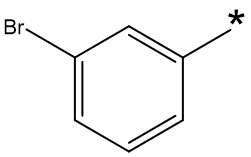** | BrC1=CC(NC(=O)NC2=NC(SCC)=NS2)=CC=C1 | 5-[3-(m-bromophenyl)ureido]-3-(ethylthio)-1,2,4-thiadiazole | **1.181** | **1.184** |
| **BC24862** | **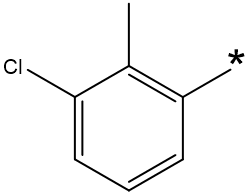** | ClC1=C(C)C(NC(=O)NC2=NC(SCC)=NS2)=CC=C1 | 5-[3-(3-chloro-2-tolyl)ureido]-3-(ethylthio)-1,2,4-thiadiazole | **6.997** | **5.676** |
| **BC24863** | **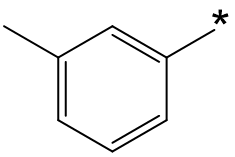** | S1N=C(SCC)N=C1NC(=O)NC1=CC(C)=CC=C1 | 3-(ethylthio)-5-[3-(m-tolyl)ureido]-1,2,4-thiadiazole | **5.089** | **2.033** |
| **BC24864** | **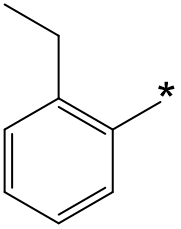** | S1N=C(SCC)N=C1NC(=O)NC1=C(CC)C=CC=C1 | 5-[3-(o-ethylphenyl)ureido]-3-(ethylthio)-1,2,4-thiadiazole | **47.35** | **63.78** |
| **BC24865** | **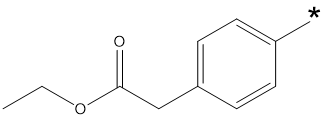** | S1N=C(SCC)N=C1NC(=O)NC1=CC=C(CC(=O)OCC)C=C1 | ethyl (p-{3-[3-(ethylthio)-1,2,4-thiadiazol-5-yl]ureido}phenyl)acetate | **27.82** | **10.72** |
| **BC24866** | **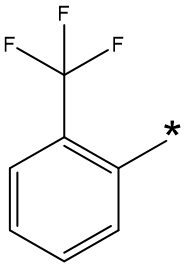** | S1N=C(SCC)N=C1NC(=O)NC1=C(C(F)(F)F)C=CC=C1 | 3-(ethylthio)-5-{3-[o-(trifluoromethyl)phenyl]ureido}-1,2,4-thiadiazole | **18.52** | **12.42** |
| **BC24867** | **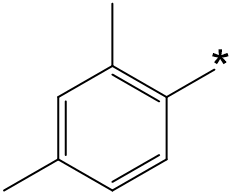** | S1N=C(SCC)N=C1NC(=O)NC1=C(C)C=C(C)C=C1 | 3-(ethylthio)-5-[3-(2,4-xylyl)ureido]-1,2,4-thiadiazole | **>100** | **13.03** |
| **BC24868** | **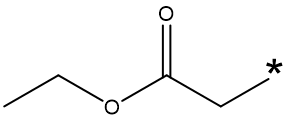** | S1N=C(SCC)N=C1NC(=O)NCC(=O)OCC | ethyl {3-[3-(ethylthio)-1,2,4-thiadiazol-5-yl]ureido}acetate | **>100** | **>100** |
| **BC24869** | **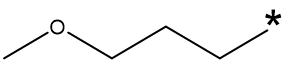** | S1N=C(SCC)N=C1NC(=O)NCCCOC | 3-(ethylthio)-5-[3-(3-methoxypropyl)ureido]-1,2,4-thiadiazole | **>100** | **56.69** |
| **BC24870** | **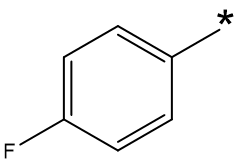** | S1N=C(SCC)N=C1NC(=O)NC1=CC=C(F)C=C1 | 3-(ethylthio)-5-[3-(p-fluorophenyl)ureido]-1,2,4-thiadiazole | **16.98** | **10.08** |
| **BC24871** | **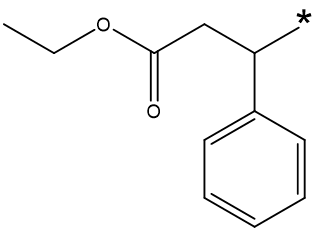** | S1N=C(SCC)N=C1NC(=O)N[C@H](C1=CC=CC=C1)CC(=O)OCC | ethyl (S)-3-{3-[3-(ethylthio)-1,2,4-thiadiazol-5-yl]ureido}-3-phenylpropionate | **>100** | **23.11** |
| **BC24872** | **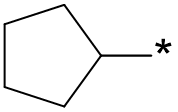** | S1N=C(SCC)N=C1NC(=O)NC1CCCC1 | 5-(3-cyclopentylureido)-3-(ethylthio)-1,2,4-thiadiazole | **>100** | **30.92** |
| **BC24873** | **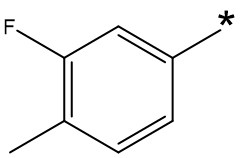** | S1N=C(SCC)N=C1NC(=O)NC1=CC(F)=C(C)C=C1 | 3-(ethylthio)-5-[3-(3-fluoro-4-tolyl)ureido]-1,2,4-thiadiazole | **8.508** | **7.74** |
| **BC24874** | **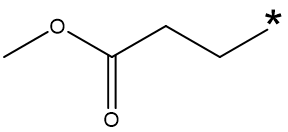** | S1N=C(SCC)N=C1NC(=O)NCCC(=O)OC | methyl 3-{3-[3-(ethylthio)-1,2,4-thiadiazol-5-yl]ureido}propionate | **>100** | **84.21** |
| **BC24875** | **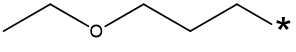** | S1N=C(SCC)N=C1NC(=O)NCCCOCC | 5-[3-(3-ethoxypropyl)ureido]-3-(ethylthio)-1,2,4-thiadiazole | **>100** | **>100** |
| **BC24876** | **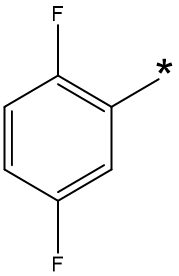** | S1N=C(SCC)N=C1NC(=O)NC1=C(F)C=CC(F)=C1 | 5-[3-(2,5-difluorophenyl)ureido]-3-(ethylthio)-1,2,4-thiadiazole | **19.45** | **5.496** |
| **BC24877** | **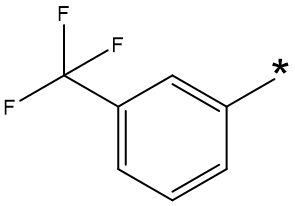** | S1N=C(SCC)N=C1NC(=O)NC1=CC(C(F)(F)F)=CC=C1 | 3-(ethylthio)-5-{3-[m-(trifluoromethyl)phenyl]ureido}-1,2,4-thiadiazole | **0.8996** | **0.733** |
| **BC24878** | **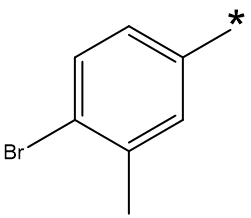** | BrC1=C(C)C=C(NC(=O)NC2=NC(SCC)=NS2)C=C1 | 5-[3-(4-bromo-3-tolyl)ureido]-3-(ethylthio)-1,2,4-thiadiazole | **3.804** | **2.548** |
| **BC24879** | **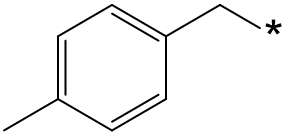** | S1N=C(SCC)N=C1NC(=O)NCC1=CC=C(C)C=C1 | 3-(ethylthio)-5-{3-[(p-tolyl)methyl]ureido}-1,2,4-thiadiazole | **27.59** | **13.29** |
| **BC24880** | **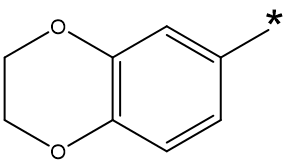** | S1N=C(SCC)N=C1NC(=O)NC1=CC2=C(C=C1)OCCO2 | 5-[3-(2,3-dihydro-1,4-benzodioxin-6-yl)ureido]-3-(ethylthio)-1,2,4-thiadiazole | **33.1** | **21.15** |
| **BC24881** | **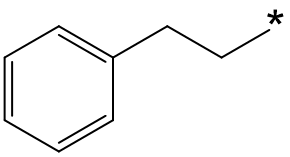** | S1N=C(SCC)N=C1NC(=O)NCCC1=CC=CC=C1 | 3-(ethylthio)-5-(3-phenethylureido)-1,2,4-thiadiazole | **29.99** | **31.88** |
| **BC24883** | **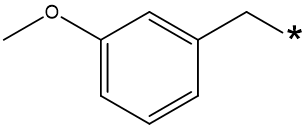** | S1N=C(SCC)N=C1NC(=O)NCC1=CC(OC)=CC=C1 | 3-(ethylthio)-5-{3-[(m-methoxyphenyl)methyl]ureido}-1,2,4-thiadiazole | **>100** | **67.76** |
| **BC24884** | **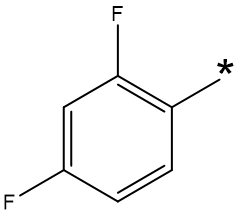** | S1N=C(SCC)N=C1NC(=O)NC1=C(F)C=C(F)C=C1 | 5-[3-(2,4-difluorophenyl)ureido]-3-(ethylthio)-1,2,4-thiadiazole | **27.55** | **16.9** |
| **BC24885** | **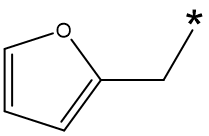** | S1N=C(SCC)N=C1NC(=O)NCC1=CC=CO1 | 3-(ethylthio)-5-(3-furfurylureido)-1,2,4-thiadiazole | **>100** | **>100** |
| **BC24886** | **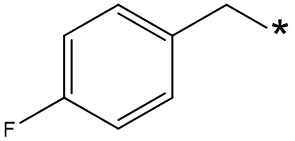** | S1N=C(SCC)N=C1NC(=O)NCC1=CC=C(F)C=C1 | 3-(ethylthio)-5-{3-[(p-fluorophenyl)methyl]ureido}-1,2,4-thiadiazole | **>100** | **>100** |
| **BC24887** | **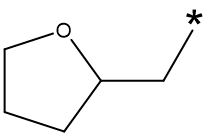** | S1N=C(SCC)N=C1NC(=O)NC[C@@H]1OCCC1 | 3-(ethylthio)-5-(3-{[(R)-perhydro-2-furyl]methyl}ureido)-1,2,4-thiadiazole | **>100** | **>100** |
| **BC24888** | **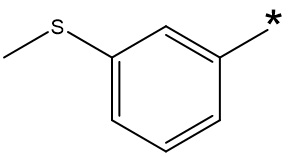** | S1N=C(SCC)N=C1NC(=O)NC1=CC(SC)=CC=C1 | 3-(ethylthio)-5-{3-[m-(methylthio)phenyl]ureido}-1,2,4-thiadiazole | **9.088** | **5.267** |
| **BC24889** | **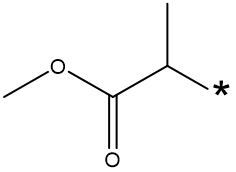** | S1N=C(SCC)N=C1NC(=O)N[C@H](C(=O)OC)C | methyl (S)-2-{3-[3-(ethylthio)-1,2,4-thiadiazol-5-yl]ureido}propionate | **>100** | **>100** |
| **BC24891** | **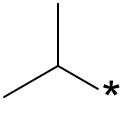** | S1N=C(SCC)N=C1NC(=O)NC(C)C | 3-(ethylthio)-5-(3-isopropylureido)-1,2,4-thiadiazole | **>100** | **>100** |
| **BC24892** | **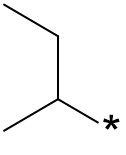** | S1N=C(SCC)N=C1NC(=O)N[C@H](CC)C | 5-{3-[(S)-1-methylpropyl]ureido}-3-(ethylthio)-1,2,4-thiadiazole | **>100** | **>100** |
| **BC24893** | **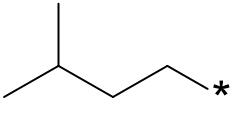** | S1N=C(SCC)N=C1NC(=O)NCCC(C)C | 3-(ethylthio)-5-(3-isopentylureido)-1,2,4-thiadiazole | **18.5** | **13.71** |
| **BC24894** | **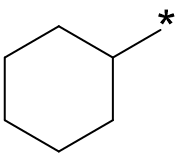** | S1N=C(SCC)N=C1NC(=O)NC1CCCCC1 | 5-(3-cyclohexylureido)-3-(ethylthio)-1,2,4-thiadiazole | **23.69** | **17.46** |
| **BC24895** | **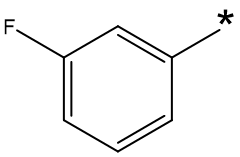** | S1N=C(SCC)N=C1NC(=O)NC1=CC(F)=CC=C1 | 3-(ethylthio)-5-[3-(m-fluorophenyl)ureido]-1,2,4-thiadiazole | **8.867** | **2.529** |
| **BC24896** | **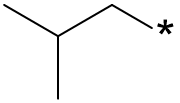** | S1N=C(SCC)N=C1NC(=O)NCC(C)C | 3-(ethylthio)-5-(3-isobutylureido)-1,2,4-thiadiazole | **29.26** | **29.92** |
| **BC24897** | **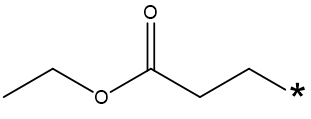** | S1N=C(SCC)N=C1NC(=O)NCCC(=O)OCC | ethyl 3-{3-[3-(ethylthio)-1,2,4-thiadiazol-5-yl]ureido}propionate | **>100** | **>100** |
| **BC24899** | **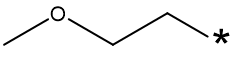** | S1N=C(SCC)N=C1NC(=O)NCCOC | 3-(ethylthio)-5-[3-(2-methoxyethyl)ureido]-1,2,4-thiadiazole | **>100** | **>100** |
| **BC24900** | **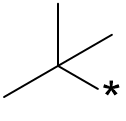** | S1N=C(SCC)N=C1NC(=O)NC(C)(C)C | 5-(3-tert-butylureido)-3-(ethylthio)-1,2,4-thiadiazole | **>100** | **>100** |
| **BC24902** | **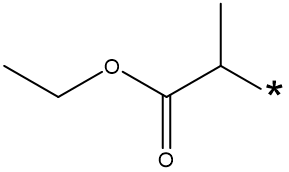** | S1N=C(SCC)N=C1NC(=O)N[C@H](C(=O)OCC)C | ethyl (S)-2-{3-[3-(ethylthio)-1,2,4-thiadiazol-5-yl]ureido}propionate | **>100** | **>100** |
| **BC24904** | **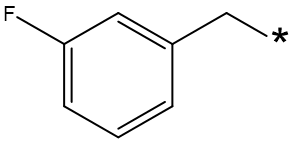** | S1N=C(SCC)N=C1NC(=O)NCC1=CC(F)=CC=C1 | 3-(ethylthio)-5-{3-[(m-fluorophenyl)methyl]ureido}-1,2,4-thiadiazole | **>100** | **>100** |
| **BC24905** | **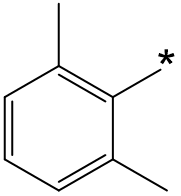** | S1N=C(SCC)N=C1NC(=O)NC1=C(C)C=CC=C1C | 3-(ethylthio)-5-[3-(2,6-xylyl)ureido]-1,2,4-thiadiazole | **22.07** | **15.79** |
| **BC24906** | **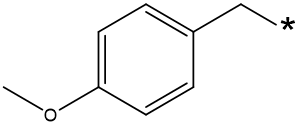** | S1N=C(SCC)N=C1NC(=O)NCC1=CC=C(OC)C=C1 | 3-(ethylthio)-5-{3-[(p-methoxyphenyl)methyl]ureido}-1,2,4-thiadiazole | **>100** | **>100** |
| **BC24907** | **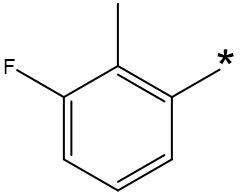** | S1N=C(SCC)N=C1NC(=O)NC1=C(C)C(F)=CC=C1 | 3-(ethylthio)-5-[3-(3-fluoro-2-tolyl)ureido]-1,2,4-thiadiazole | **9.854** | **3.358** |
| **BC24908** | **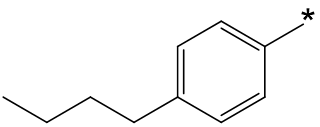** | S1N=C(SCC)N=C1NC(=O)NC1=CC=C(CCCC)C=C1 | 5-[3-(p-butylphenyl)ureido]-3-(ethylthio)-1,2,4-thiadiazole | **30** | **18.26** |
| **BC24910** | **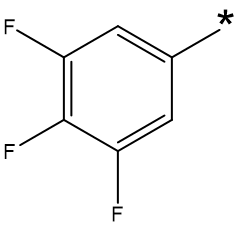** | S1N=C(SCC)N=C1NC(=O)NC1=CC(F)=C(F)C(F)=C1 | 3-(ethylthio)-5-[3-(3,4,5-trifluorophenyl)ureido]-1,2,4-thiadiazole | **2.231** | **0.420** |
| **BC24911** | **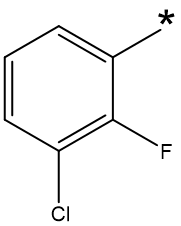** | ClC1=C(F)C(NC(=O)NC2=NC(SCC)=NS2)=CC=C1 | 5-[3-(3-chloro-2-fluorophenyl)ureido]-3-(ethylthio)-1,2,4-thiadiazole | **9.716** | **8.13** |
| **BC24912** | **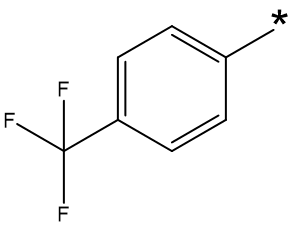** | S1N=C(SCC)N=C1NC(=O)NC1=CC=C(C(F)(F)F)C=C1 | 3-(ethylthio)-5-{3-[p-(trifluoromethyl)phenyl]ureido}-1,2,4-thiadiazole | **1.761** | **0.672** |
| **BC24913** | **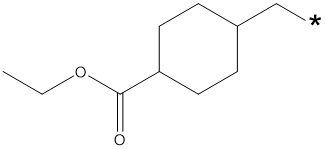** | S1N=C(SCC)N=C1NC(=O)NCC1CCC(C(=O)OCC)CC1 | ethyl 4-({3-[3-(ethylthio)-1,2,4-thiadiazol-5-yl]ureido}methyl)cyclohexanecarboxylate | **>100** | **17.37** |
| **BC24915** | **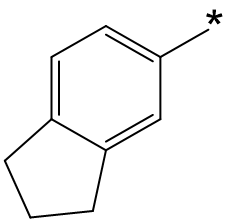** | S1N=C(SCC)N=C1NC(=O)NC1=CC2=C(C=C1)CCC2 | 3-(ethylthio)-5-[3-(5-indanyl)ureido]-1,2,4-thiadiazole | **34** | **17.43** |
| **BC24916** | **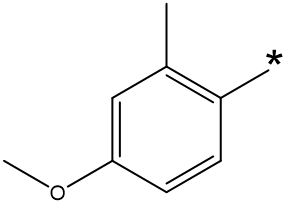** | S1N=C(SCC)N=C1NC(=O)NC1=C(C)C=C(OC)C=C1 | 3-(ethylthio)-5-[3-(4-methoxy-2-tolyl)ureido]-1,2,4-thiadiazole | **>100** | **17.22** |

**Supplemental Table 2. Antibody and Resources**

| **REAGENT** | **SOURCE** | **IDENTIFIER** |
| --- | --- | --- |
| **Antibodies** | | |
| β-actin | Santa Cruz Biotechnology | Cat# sc-47778 |
| V5 Tag | Invitrogen | Cat# R960-25 |
| HA Tag | Cell Signaling Technologies | Cat# 3724 |
| Flag (M2) Tag | Sigma-Aldrich | Cat# F3165 |
| GFP Tag | Cell Signaling Technologies | Cat# 2956 |
| OC43 (541-8F) | Sigma-Aldrich | Cat# MAB9012 |
| HiBiT | Promega | Cat# N7210 |
| FBXO5 | Proteintech | Cat# 10872-1-AP |
| β-TrCP | Cell Signaling Technologies | Cat# 4394 |
| β-Catenin | Cell Signaling Technologies | Cat# 9562 |
| WEE1 | Proteintech | Cat# 29474-1-AP |
| CDC25A | Proteintech | Cat# 68457-1-Ig |
| NF-κB p100 | Cell Signaling Technologies | Cat# 4882 |
| Cyclin D1 | Proteintech | Cat# 60186-1-Ig |
| K48-linkage Specific Polyubiquitin | Cell Signaling Technologies | Cat# 4289 |
| TAF1 | Cell Signaling Technologies | Cat# 12781 |
| Phospho-Serine | Sigma-Aldrich | Cat# AB1603 |
| SARS-CoV-2 Nucleoprotein | Invitrogen | Cat# MA1-7403 |
| ISG15 | Santa Cruz Biotechnology | Cat# sc-166755 |
| MDA5 | Cell Signaling Technologies | Cat# 5321 |
| RAD51 | Cell Signaling Technologies | Cat# 8875 |
| CDH1 | Sigma-Aldrich | Cat# MABT1323 |
| Donkey anti-Mouse IgG (H+L) Cross-Adsorbed Secondary Antibody, CF®594 | Biotium | Cat# 20115 |
| Goat anti-Rabbit IgG (H+L) HRP | Invitrogen | Cat# 31460 |
| Goat anti-Mouse IgG (H+L) HRP | Invitrogen | Cat# 31430 |
| **Chemicals, Peptides, Recombinant Proteins and Commercial Assays** | | |
| FDA-Approved Compound Library | Selleck | L1300 |
| Cycloheximide | Enzo Life Sciences | ALX-380-269-G005 |
| MG132 | MedChemExpress | HY-13259 |
| Leupeptin | MedChemExpress | HY-18234A |
| Poly (I:C) | MedChemExpress | HY-107202 |
| BAY-299 | MedChemExpress | HY-107424 |
| GS143 | MedChemExpress | HY-110261 |
| Hoechst33342 | Invitrogen | H3570 |
| Nano-Glo HiBiT Lytic | Promega | N3030 |
| CellTiter-Glo® 2.0 | Promega | G9241 |
| Opti-MEM I Reduced Serum Medium | Gibco | 31985062 |
| Lullaby Transfection Reagent | OZ Biosciences | LL73000 |
| Lipofectamine 3000 Transfection Reagent | Thermo Scientific | L3000015 |
| pcDNA3.1 Directional TOPO Expression Kit | Invitrogen | K490001 |
| QuikChange II XL Site-Directed Mutagenesis Kit | Aglient | 200521 |
| pFC37K-HiBiT vector | Promega | N2391 |
| GFP-TAF1 plasmid | Addgene | 65395 |
| HA-FBXO5 plasmid | Addgene | 52509 |
| Flag-betaTrCP plasmid | Addgene | 10865 |
| TnT Transcription/Translation kit | Promega | L1170 |
| Dynabeads His-Tag Isolation and Pulldown | Invitrogen | 10103D |
| Pierce Protein A/G Magnetic Beads | Thermo Scientific | 88802 |
| Pierce Anti-HA Magnetic Beads | Thermo Scientific | 88837 |
| EnGen® Spy Cas9 NLS | New England Biolads | M0646M |
| Lipofectamine RNAiMAX | Thermo Scientific | 13778150 |
| High-Capacity cDNA Reverse Transcription Kit | Applied Biosystems | 4368814 |
| SYBR Green PCR Master Mix | Applied Biosystems | 4364344 |
| RNeasy Plus Mini Kit | Qiagen | 74134 |
| In-Fusion Cloning | Takara | 638910 |
| Gateway Cloning | ThermoFisher | 11791019 |
| Phusion polymerase | New England Biolabs | M0530 |
| Quick CIP | New England Biolabs | M0525 |
| Quick Ligation Kit | New England Biolabs | M2200 |

**Supplemental Table 3. Oligonucleotides**

| **Oligonucleotides** | **Sequence** | **Source** |
| --- | --- | --- |
| Negative Control siRNA | Control DsiRNA Negative Control (DS NC1) rCrGrUrUrArArUrCrGrCrGrUrArUrArArUrArCrGrCrGrUAT rArUrArCrGrCrGrUrArUrUrArUrArCrGrCrGrArUrUrArArCrGrArC | IDT |
| FBXO5 siRNA | hs.Ri.FBXO5.13.1 rCrUrCrUrUrArCrUrCrArArUrUrGrArUrArCrCrArArCrAGA rUrCrUrGrUrUrGrGrUrArUrCrArArUrUrGrArGrUrArArGrArGrGrA | IDT |
| TAF1 siRNA | hs.Ri.TAF1.13.1 rCrCrUrGrGrArArArGrGrUrGrUrUrArCrGrUrUrUrUrCrUAC rGrUrArGrArArArArCrGrUrArArCrArCrCrUrUrUrCrCrArGrGrUrC | IDT |
| BTRC siRNA | hs.Ri.BTRC.13.1 rGrUrGrGrArUrUrCrUrCrArGrArCrArUrGrArUrArCrUrCTC rGrArGrArGrUrArUrCrArUrGrUrCrUrGrArGrArArUrCrCrArCrUrG | IDT |
| FBXO5-HiBiT CRISPR Knock in | gRAN sequence: CCACGTCGGCTGGCATGAGC | IDT |
|  | HDR donor sequence (+): AGCGGGGTGGCCGTAGGGCGCAGCTGCAGGGGCGCCGGCTA CCGGAACCGCCGCTAATCTTCTTGAACAGCCGCCAGCCGCTCA CCATGCCAGCCGACGTGGAGTCTGCCTCAGGTGGAGGAACC | IDT |
| SARS-CoV-2 NSP7 (WT) in pcDNA3.1D-V5-HIS | Fwd: caccATGAGCAAGATGAGCGACGTAA Rev: CTGCAATGTCGCCCGGTTGT | IDT |
| SARS-CoV-2 NSP7 K3R | Fwd: CGCCATGAGCcgcATGAGCGACG Rev: ATCGCTAGCCCTATAGTG | IDT |
| SARS-CoV-2 NSP7 K8R | Fwd: GAGCGACGTAcgcTGCACGTCAGTTG Rev: ATCTTGCTCATGGCGATC | IDT |
| SARS-CoV-2 NSP7 K28R | Fwd: GTCTAGTTCCcgcCTCTGGGCTCAATG Rev: TCCACTCTGAGTTGTTGC | IDT |
| SARS-CoV-2 NSP7 K44R | Fwd: TCTGTTGGCGcgcGATACCACGG Rev: ATGTCATTGTGAAGCTGAAC | IDT |
| SARS-CoV-2 NSP7 K52R | Fwd: GGCATTCGAGcgcATGGTCTCACTTC Rev: TCCGTGGTATCTTTCGCC | IDT |
| SARS-CoV-2 NSP7 K71R | Fwd: CGATATCAATcgcTTGTGTGAGGAAATGCTCGACAAC Rev: ACCGCCCCTTGCATTGAC | IDT |
| SARS-CoV-2 NSP7 S16A | Fwd: TGTTCTCTTGgcgGTCCTGCAACAAC Rev: ACTGACGTGCATTTTAC | IDT |
| SARS-CoV-2 NSP7 S26A | Fwd: AGTGGAGTCTgcgTCCAAGCTCTG Rev: CTGAGTTGTTGCAGG | IDT |
| SARS-CoV-2 NSP7 S55A | Fwd: GAAGATGGTCgcgCTTCTGAGCG Rev: TCGAATGCCTCCGTG | IDT |
| SARS-CoV-2 NSP7 S58A | Fwd: CTCACTTCTGgcgGTTCTCCTGTCAATG Rev: ACCATCTTCTCGAATGC | IDT |
| SARS-CoV-2 NSP7 D5A | Fwd: CAAGATGAGCgcgGTAAAATGCAC Rev: CTCATCTGCAATGTCG | IDT |
| SARS-CoV-2 NSP7 T9A | Fwd: CGTAAAATGCgcgTCAGTTGTTC Rev: TCGCTCATCTTGCTCATC | IDT |
| SARS-CoV-2 NSP7 M52A | Fwd: ATTCGAGAAGgcgGTCTCACTTCTGAG Rev: GCCTCCGTGGTATCTTTC | IDT |
| HCoV-OC43 NSP7 (WT) in pcDNA3.1D-V5-HIS | Fwd: caccATGTCAAAATTGACTGATGT Rev: CTGCAAAACAGTATTGTCCTT | IDT |
| HCoV-OC43 NSP7 K3R | Fwd: GTAAATGTCAcgcTTGACTGATGTCAAATGTGC Rev: TGCAAAACAGTATTGTCC | IDT |
| HCoV-OC43 NSP7 K8R | Fwd: GACTGATGTCcgcTGTGCTAATGTCG Rev: AATTTTGACATTTACTGCAAAAC | IDT |
| HCoV-OC43 NSP7 K28R | Fwd: TTCTAATTCTcgcTTGTGGCATTATTGTAGCAC Rev: GCAACATGCAAATGTTG | IDT |
| HCoV-OC43 NSP7 K52R | Fwd: TGCTTTTGAAcgcCTTGCTCAGTTATTAATTGTTTTG Rev: ACACTCAGATCCGAAG | IDT |
| HCoV-OC43 NSP7 K70R | Fwd: TGTGGATAGCcgcTGCCTGACTAGTATTG Rev: GCAGCTGGATTAGCAAAC | IDT |
| HCoV-OC43 NSP7 K84R | Fwd: TGATTACGCAcgcGACAATACTGTTTTGCAG Rev: TCGCAAACTTCTTCAATAC | IDT |
| qPCR IL-6 | Fwd: ACTCACCTCTTCAGAACGAATTG Rev: CCATCTTTGGAAGGTTCAGGTTG | IDT |
| qPCR CXCL1 | Fwd: AGCTTGCCTCAATCCTGCATCC Rev: TCCTTCAGGAACAGCCACCAGT | IDT |
| qPCR IFN-β | Fwd:ATGACCAACAAGTGTCTCCTCC  Rev:GGAATCCAAGCAAGTTGTAGCTC | IDT |
| qPCR IFN-λ | Fwd: GAGGCCCCCAAAAAGGAGTC Rev: AGGTTCCCATCGGCCACATA | IDT |
| qPCR CXCL10 | Fwd: GTGGCATTCAAGGAGTACCTC Rev: TGATGGCCTTCGATTCTGGATT | IDT |
| qPCR CXCL11 | Fwd: GACGCTGTCTTTGCATAGGC Rev: GGATTTAGGCATCGTTGTCCTTT | IDT |
| qPCR FBXO5 | Fwd: GCTGTCATGTATTGGGTCACC Rev: GTCTACTGGTCTCTAGTGCTTCT | IDT |
| qPCR BTRC | Fwd: ACCAACATGGGCACATAAACTC Rev: TGGCATCCAGGTATGACAGAAT | IDT |
| qPCR SARS-CoV-2 NSP7 | Fwd: ATGAGCAAGATGAGCGACGT Rev: TTGATATCGACCGCCCCTTG | IDT |
| qPCR OC43 gRNA | Fwd: ATGTGGTGTAAAGCAGGAACA Rev: GCAAGAACAGTCCACGGTATA | IDT |
| qPCR OC43-NP | Fwd: CATACTCTGACGGTCACAATAATA Rev: ACCTTAGCAACAGTCATATAAGC | IDT |
| qPCR β-actin | Fwd: CACCATTGGCAATGAGCGGTTC Rev: AGGTCTTTGCGGATGTCCACGT | IDT |
| SARS-CoV-2 NSP7 plasmid cDNA | atgagcaagatgagcgacgtaaaatgcacgtcagttgttctcttgagcgtcctgcaacaactcagagtggagtctagttccaagctctgggctcaatgcgttcagcttcacaatgacattctgttggcgaaagataccacggaggcattcgagaagatggtctcacttctgagcgttctcctgtcaatgcaaggggcggtcgatatcaataaattgtgtgaggaaatgctcgacaaccgggcgacattgcag |  |
| HCoV-OC43 NSP7 plasmid cDNA | atgtcaaaatttgactgatgtcaaatgtgctaatgtcgtcttgcttaattgcttgcaacatttgcatgttgcttctaattctaagttgtggcattattgtagcactttgcacaatgaaatacttgccacttcggatctgagtgttgcttttgaaaagcttgctcagttattaattgttttgtttgctaatccagctgctgtggatagcaagtgcctgactagtattgaagaagtttgcgatgattacgcaaaggacaatactgttttgcag |  |
|  |  |  |
