# Supplementary material for: NSP7 Molecular Degrader Attenuates Coronaviral Infection Through the β‐TrCP1/FBXO5 Axis
Source: Adv Sci (Weinh). 2025 Jun 27;12(36):e00798. doi: 10.1002/advs.202500798 (PMC12463105; doi:10.1002/advs.202500798)

ADVANCED SCIENCE
Open Access

Supporting Information

for *Adv. Sci.*, DOI 10.1002/advs.202500798

NSP7 Molecular Degrader Attenuates Coronaviral Infection Through the β-TrCP1/FBXO5 Axis

*Yao Tong, Travis B. Lear, Ferhan Tuncer, John J. Villandre, Áine N. Boudreau, Bo Lin, Irene Alfaras, Jason R. Kennerdell, Daniel P. Camarco, Mads B. Larsen, Yun Hua, Yanwen Chen, Meigin E. Chandler, Ricardo Pineda, Simon M. Barratt-Boyes, John W. Evankovich, Toren Finkel, Yuan Liu and Bill B. Chen**

Fig. 1 C

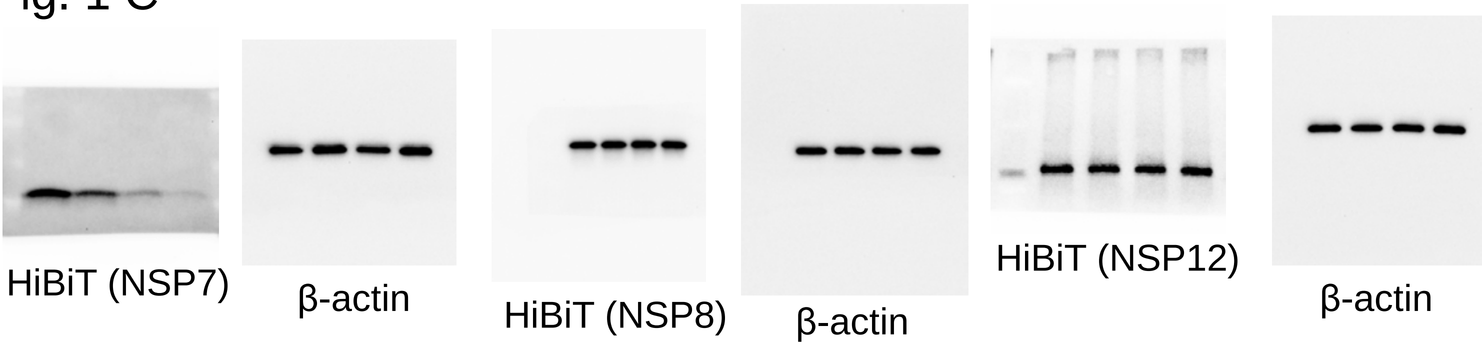

Fig. 1 D

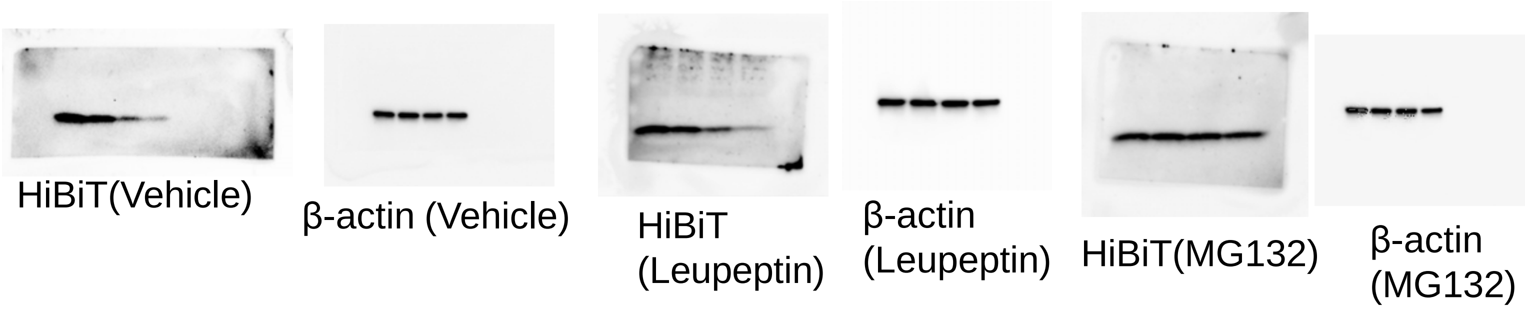

Fig. 1 E

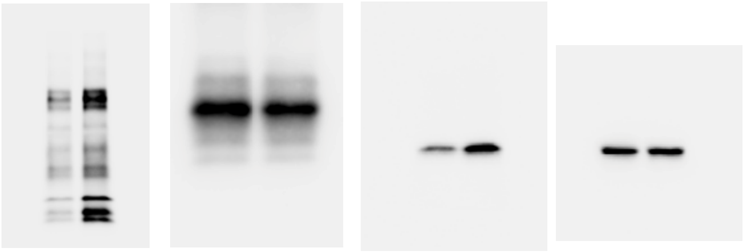

Fig. 1 J

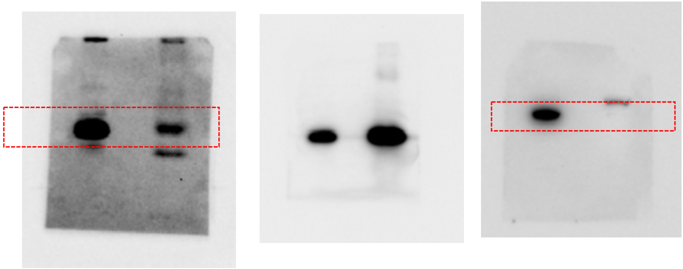

Fig. 1 L

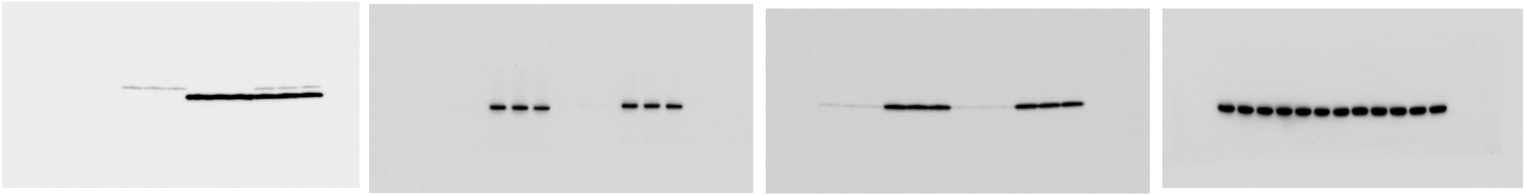

Fig. 1 M

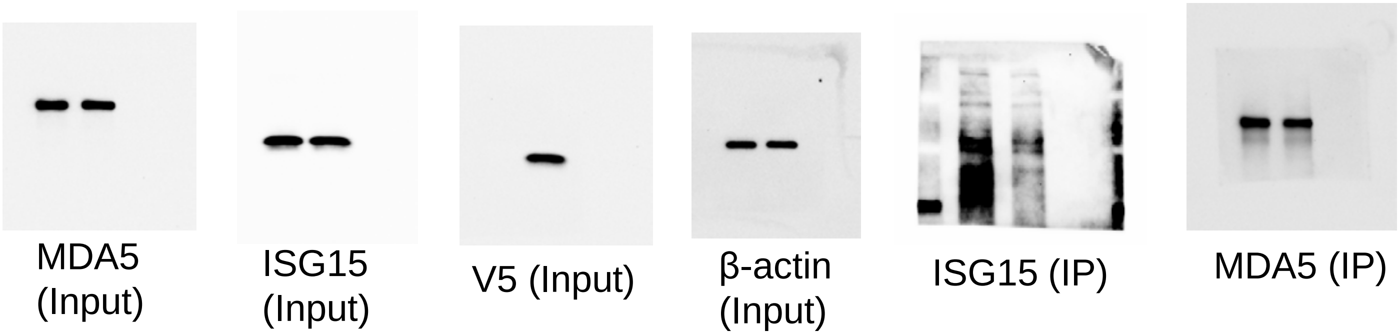

Fig. 2 B

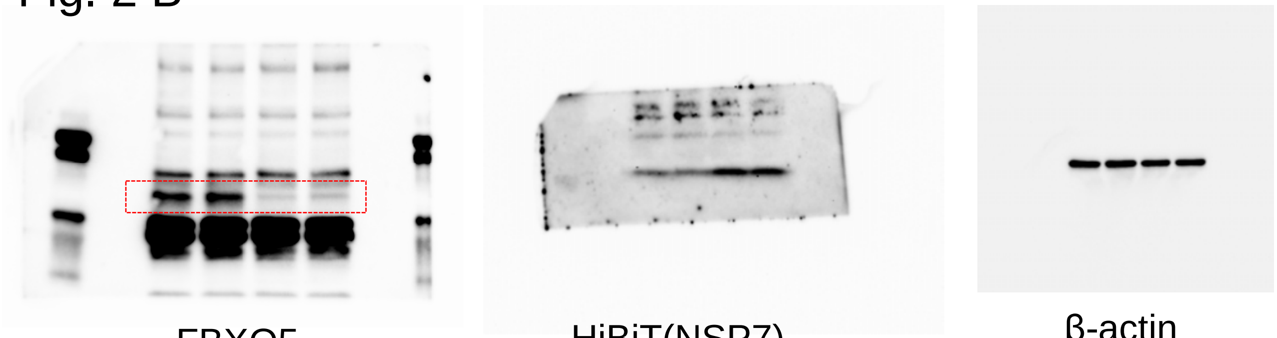

FBXO5 | HiBiT(NSP7) | β-actin

Fig. 2 D

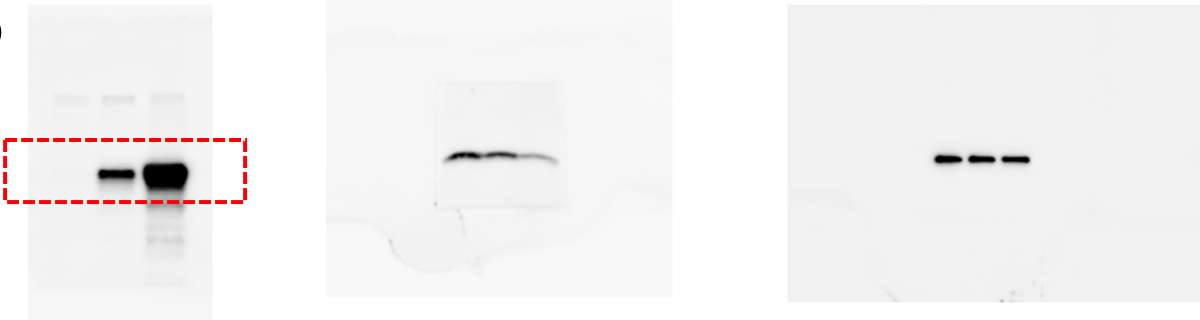

HA (FBXO5) | HiBiT(NSP7) | β-actin

Fig. 2 G

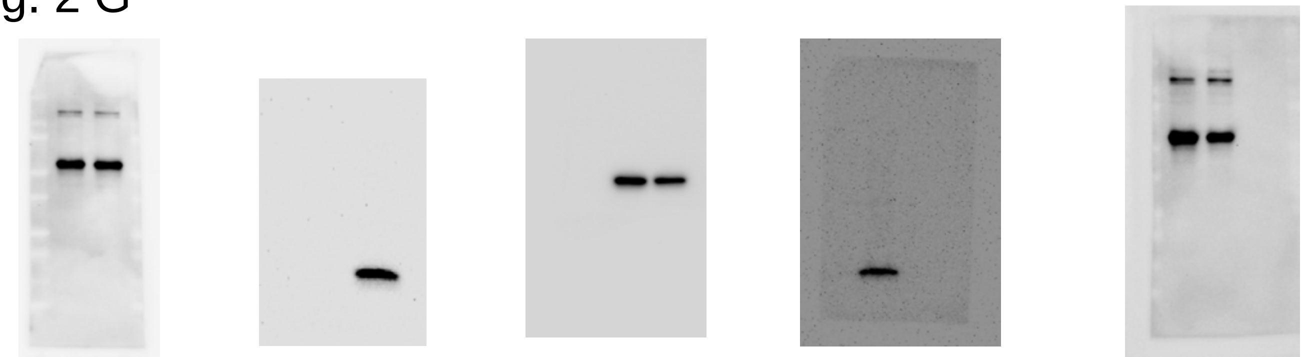

HA (Input) | HiBiT (Input) | β-actin | HiBiT (PD) | HA (PD)

Fig. 2 H

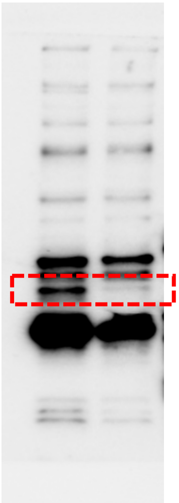

FBXO5 (Input)

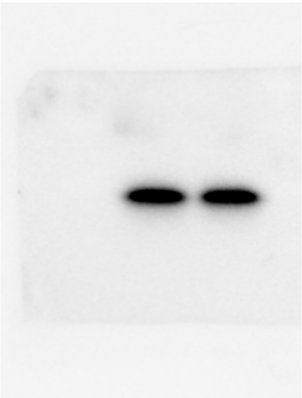

NSP7-V5 (Input)

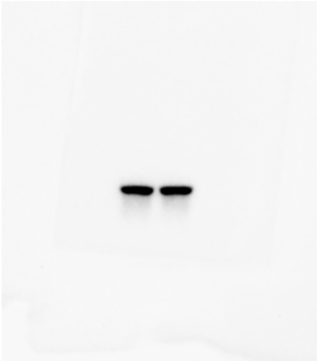

β-actin

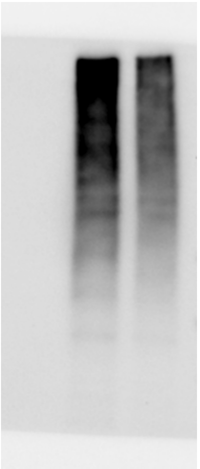

K48-Ub (PD)

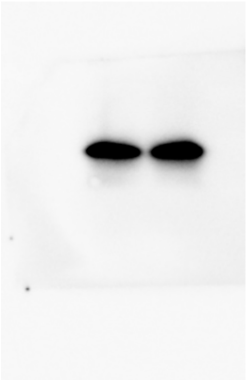

NSP7-V5 (PD)

Fig. 2 I

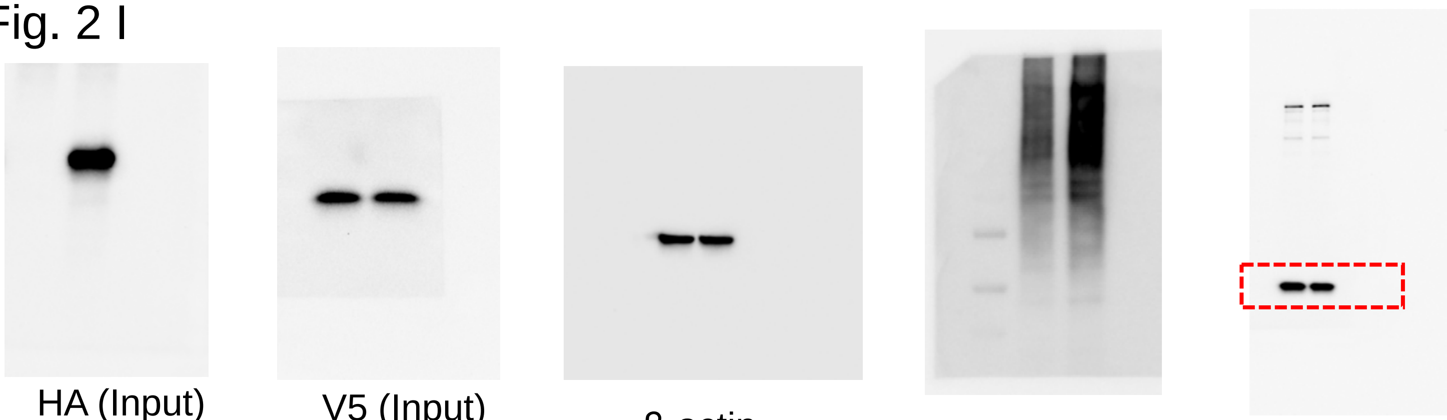

HA (Input) V5 (Input) β-actin K48-Ub (PD) V5 (PD)

Fig. 2 J

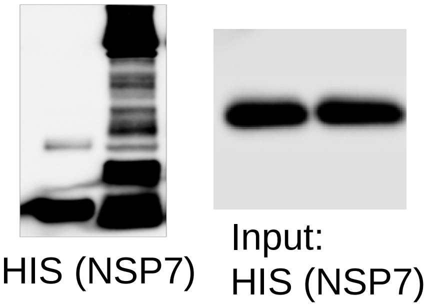

HIS (NSP7) Input: HIS (NSP7)

Fig. 2 M

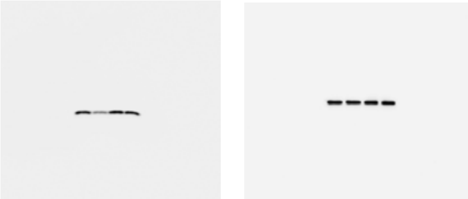

NSP7-HiBiT β-actin

Fig. 2 N

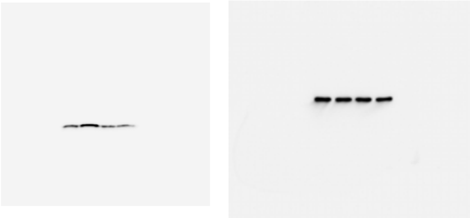

NSP7-HiBIT β-actin

Fig. 2 O

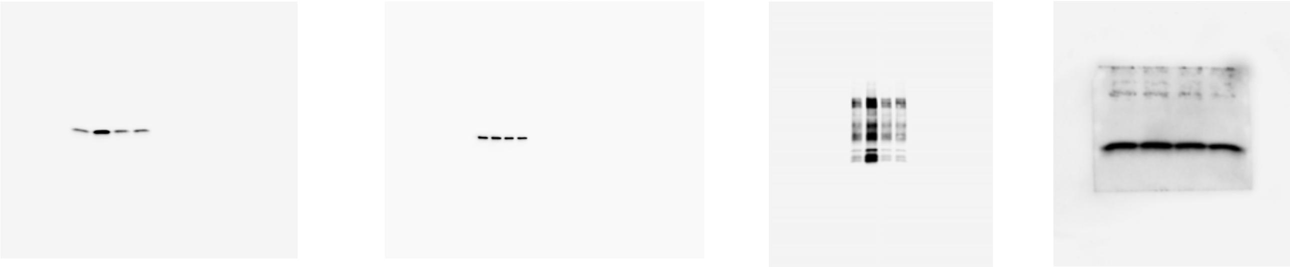

HiBiT (Input) β-actin K48-Ub (IP) HiBiT (IP)

Fig. 2 P

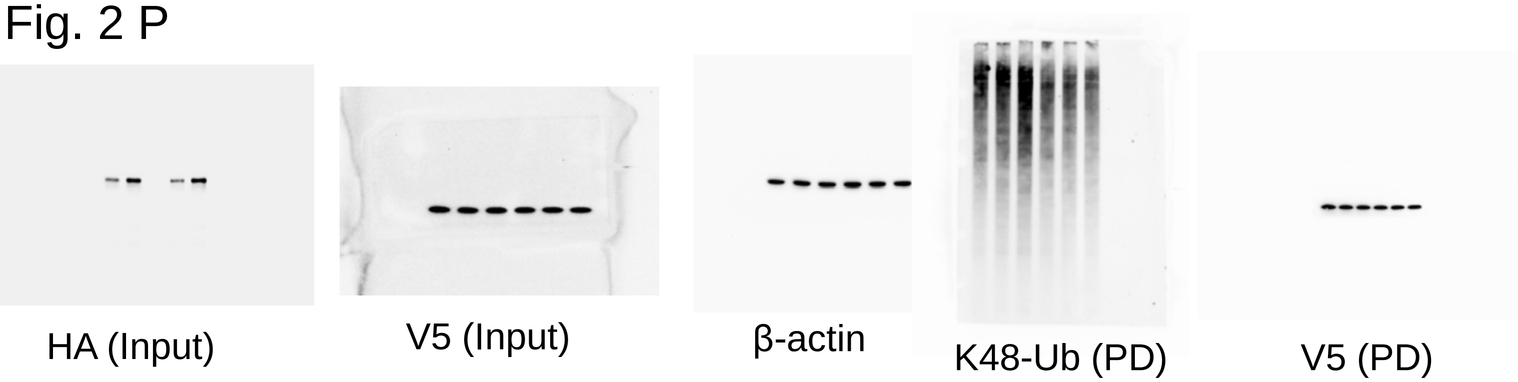

HA (Input) V5 (Input) β-actin K48-Ub (PD) V5 (PD)

Fig. 2 R

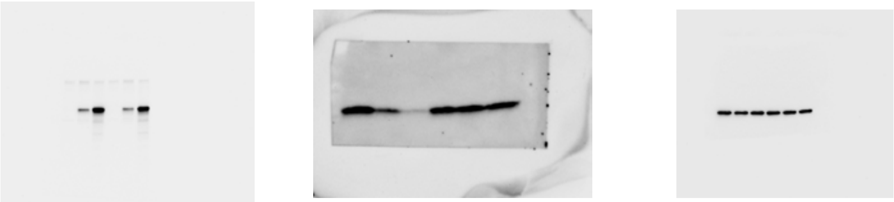

HA HiBiT (NSP7) β-actin

Fig. 3 B

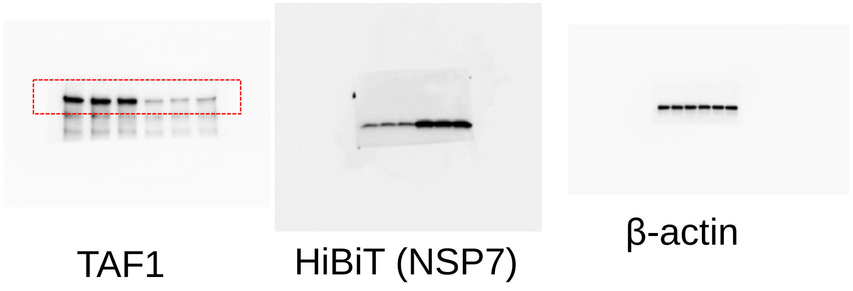

Fig. 3 C

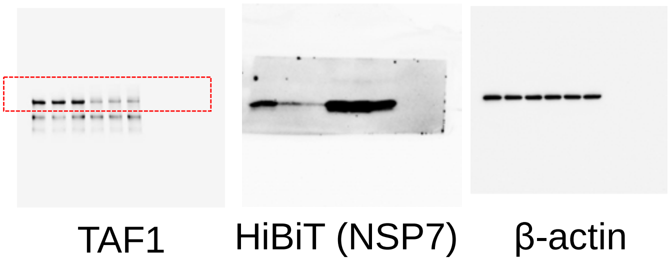

Fig. 3 D

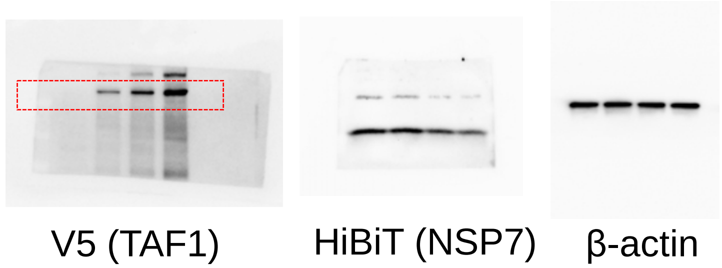

Fig. 3 E

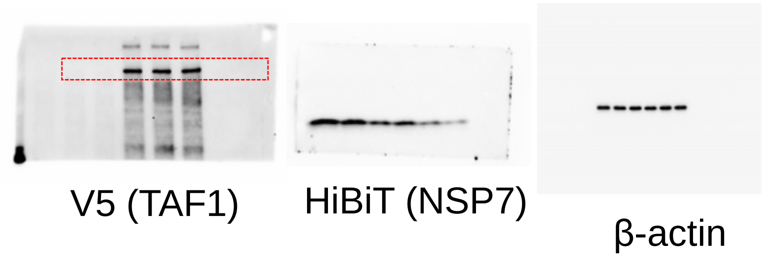

Fig. 3 F

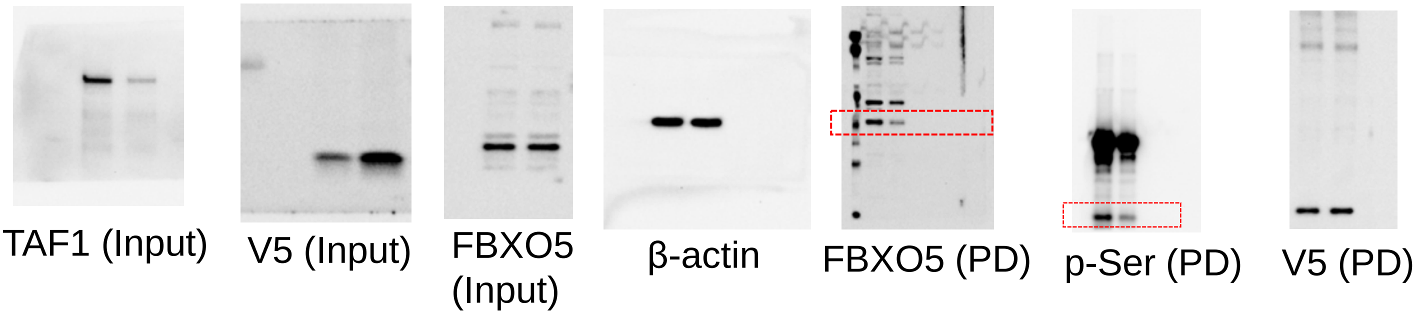

Fig. 3 G

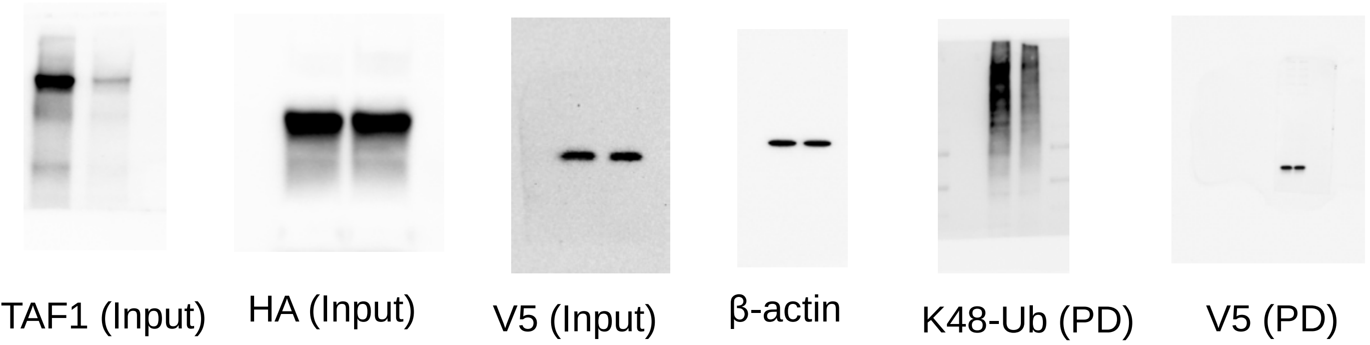

Fig. 3 H

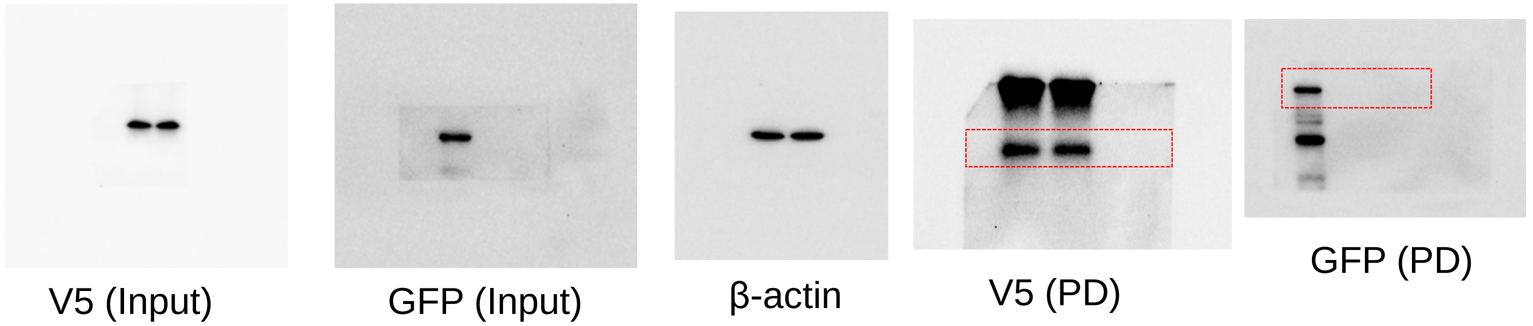

Fig. 3 K

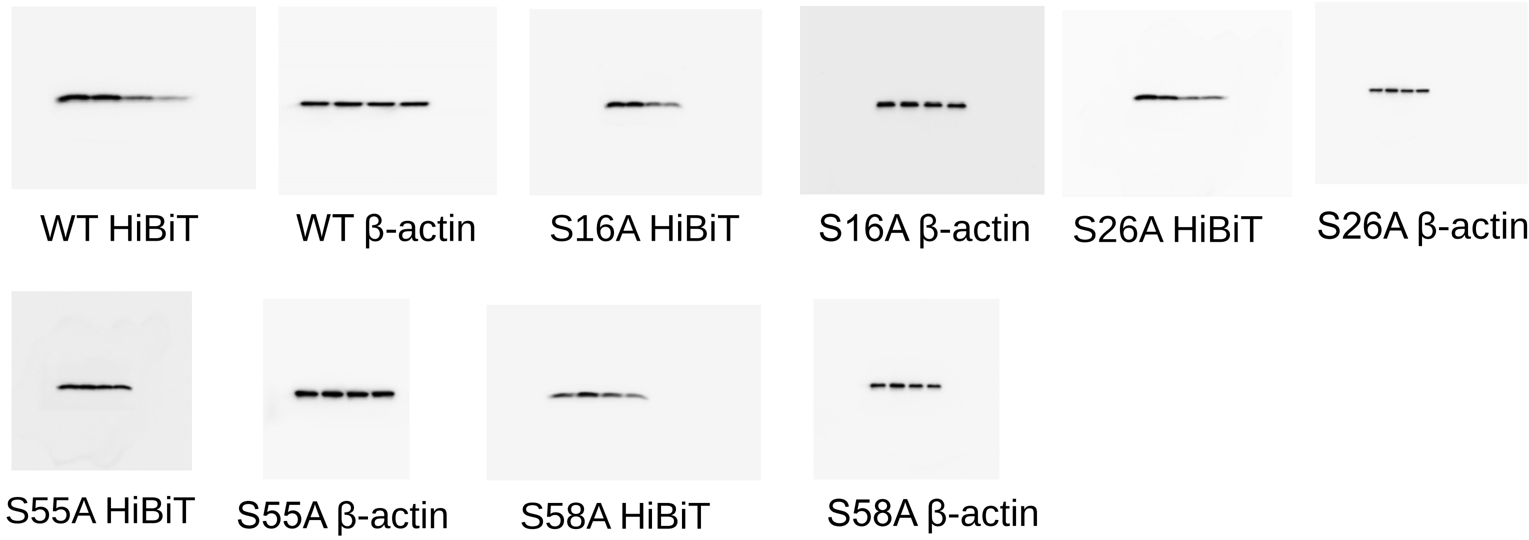

Fig. 3 L

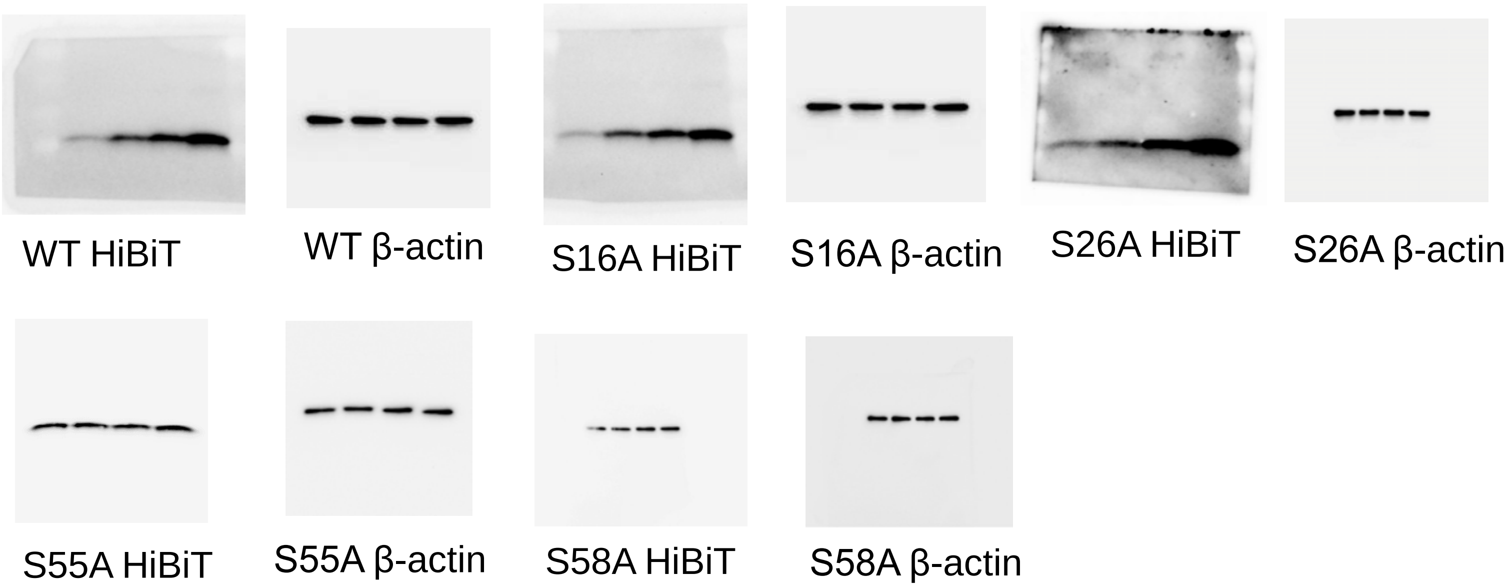

Fig. 3 N

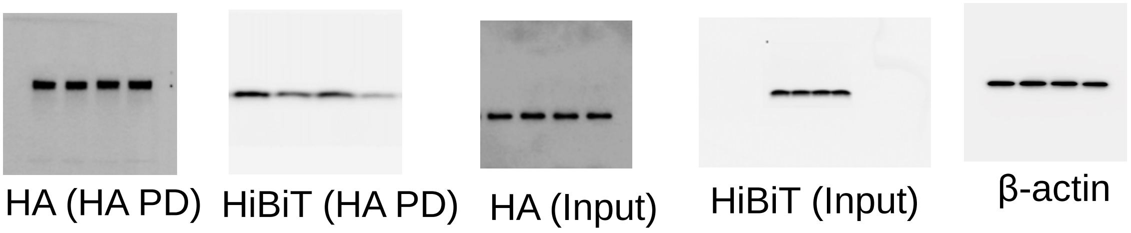

Fig. 3 O

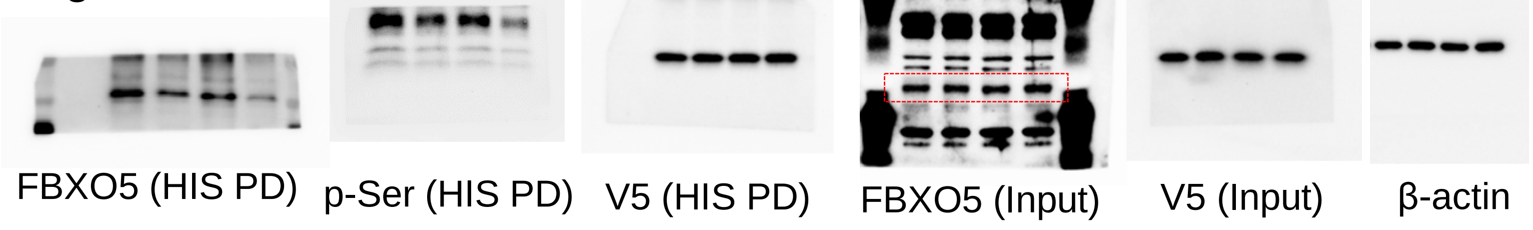

Fig. 3 P

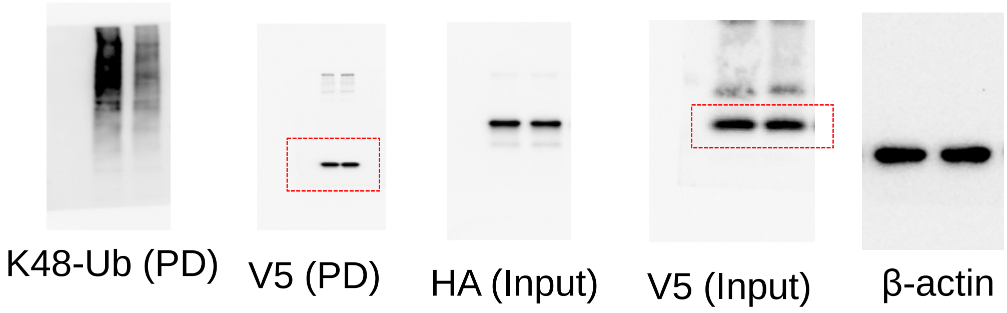

Fig. 4 B

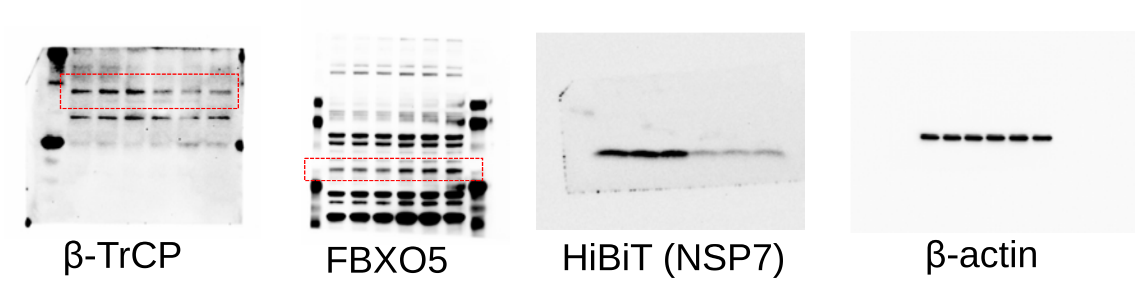

Fig. 4 C

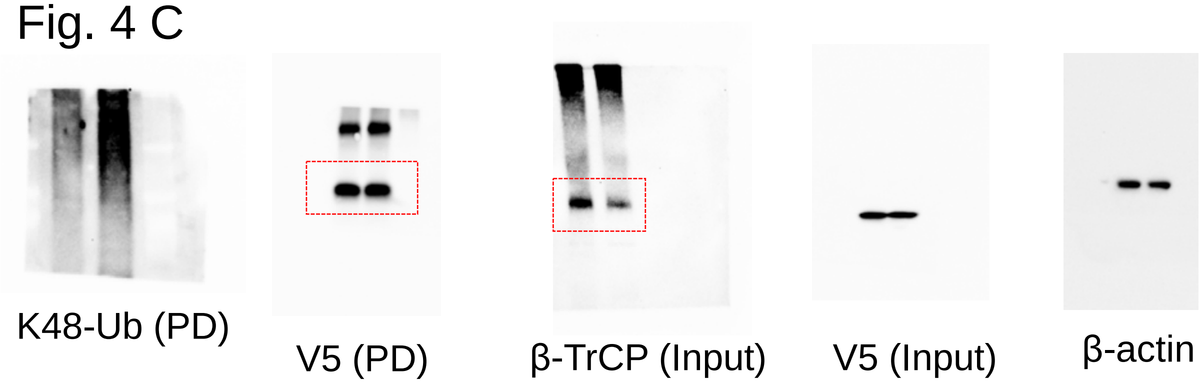

Fig. 4 D

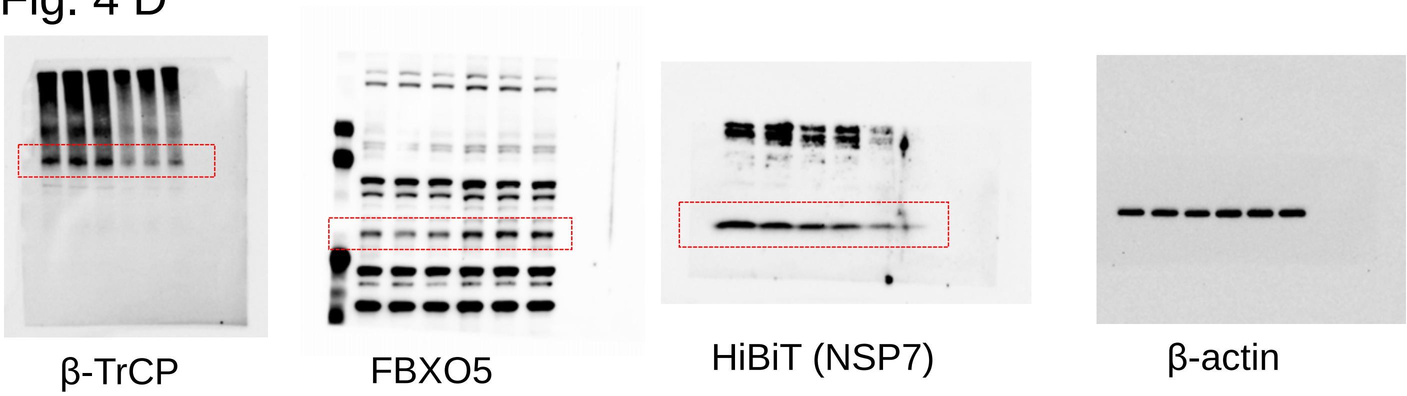

Fig. 4 E

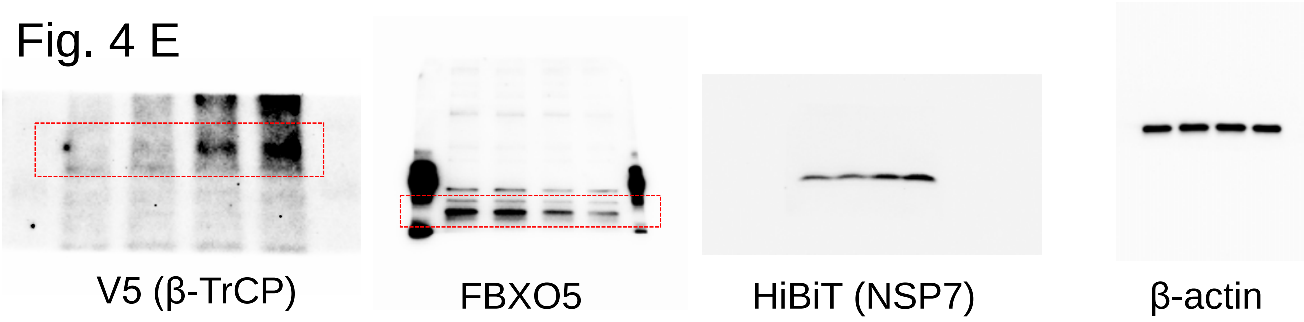

Fig. 4 F

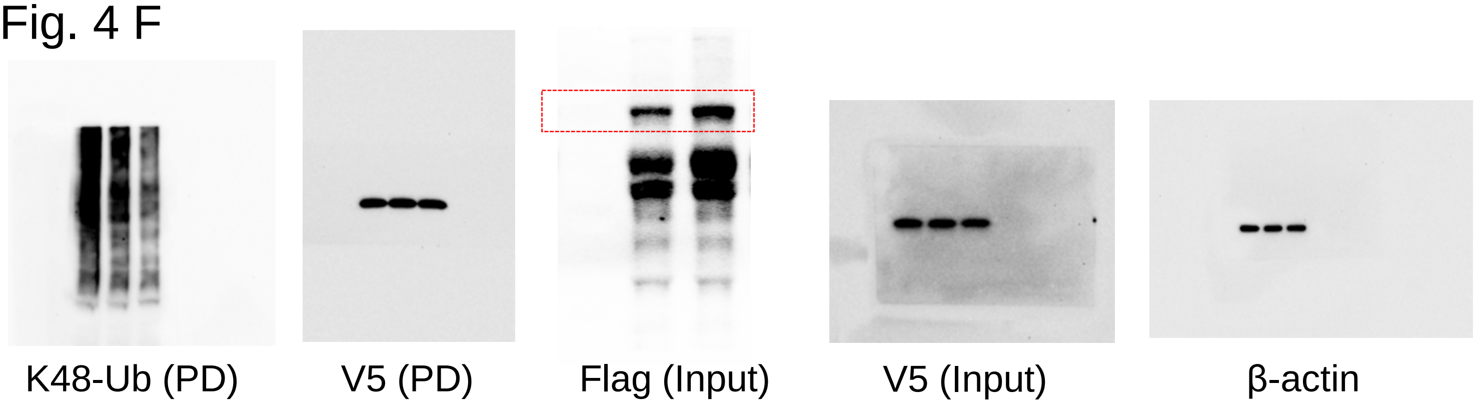

Fig. 4 G

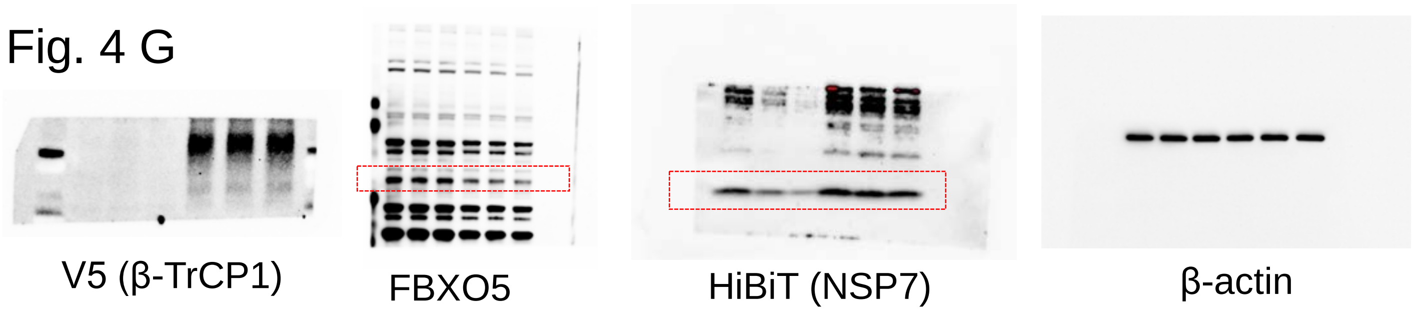

Fig. 4 H

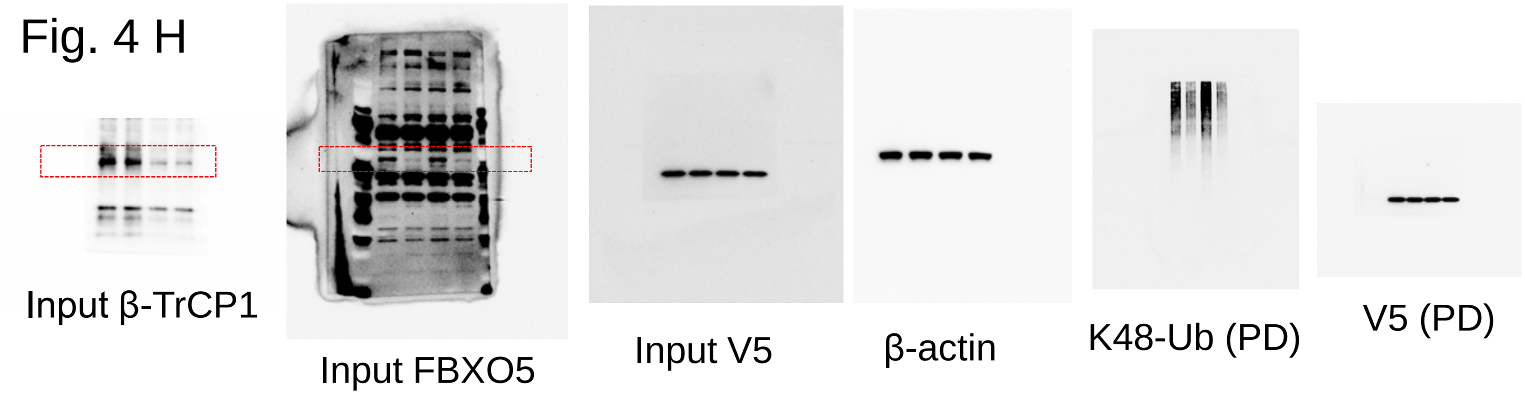

Fig. 6 A

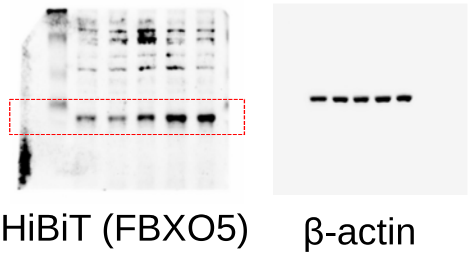

Fig. 6 B

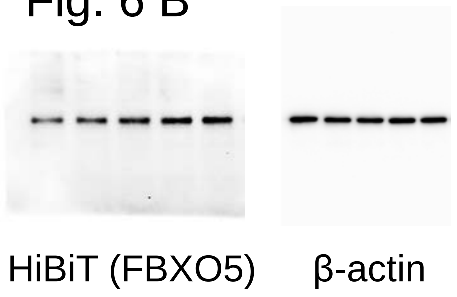

Fig. 6 C

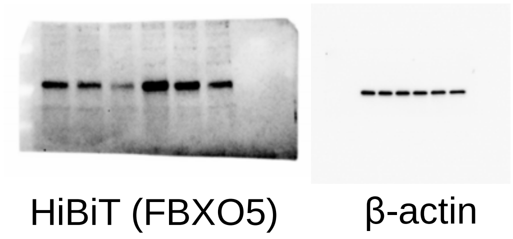

Fig. 6 D

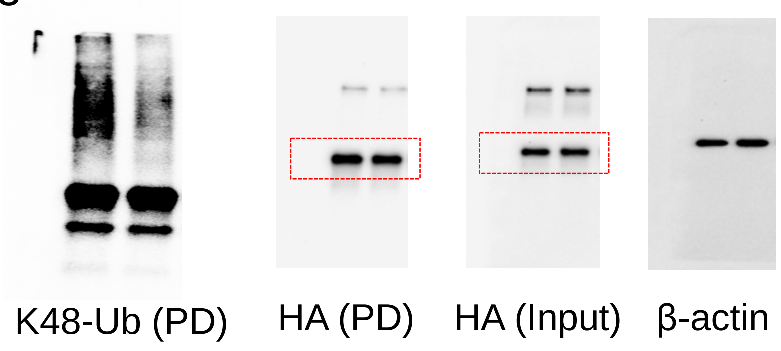

Fig. 6 E

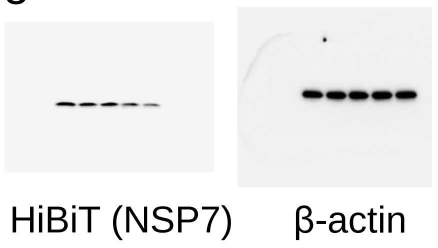

Fig. 6 F

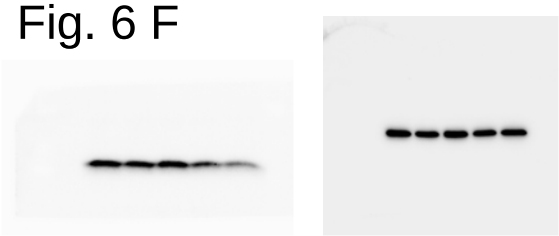

HiBiT (NSP7) β-actin

Fig. 6 G

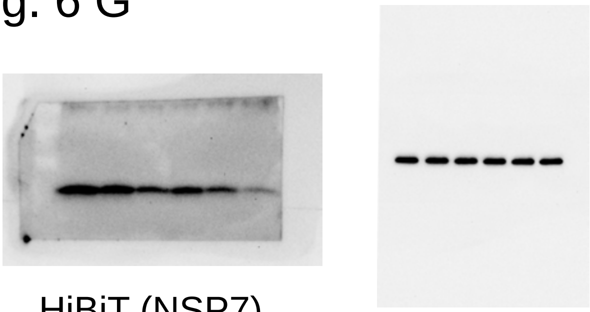

HiBiT (NSP7) β-actin

Fig. 6 H

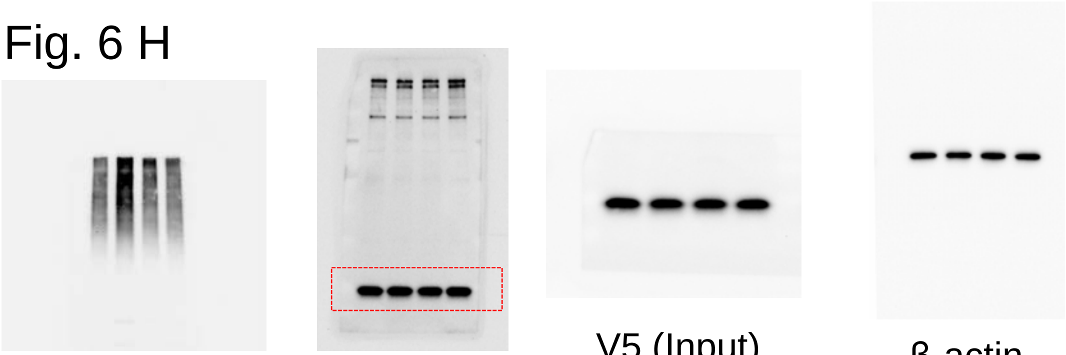

K48-Ub (PD) V5 (PD) V5 (Input) β-actin

Fig. 6 I

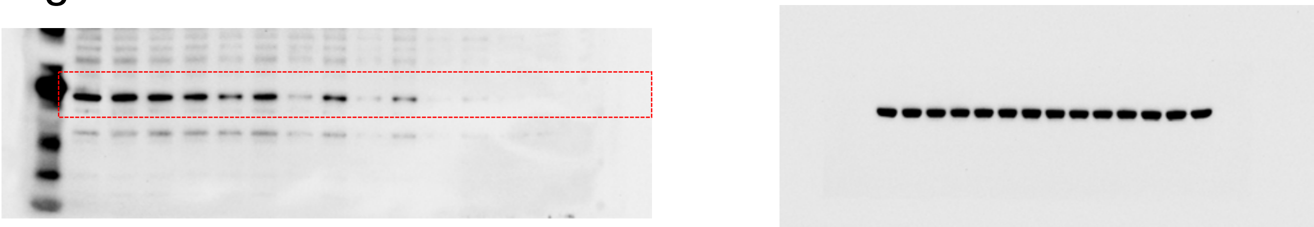

FBXO5 β-actin

Fig. 6 K

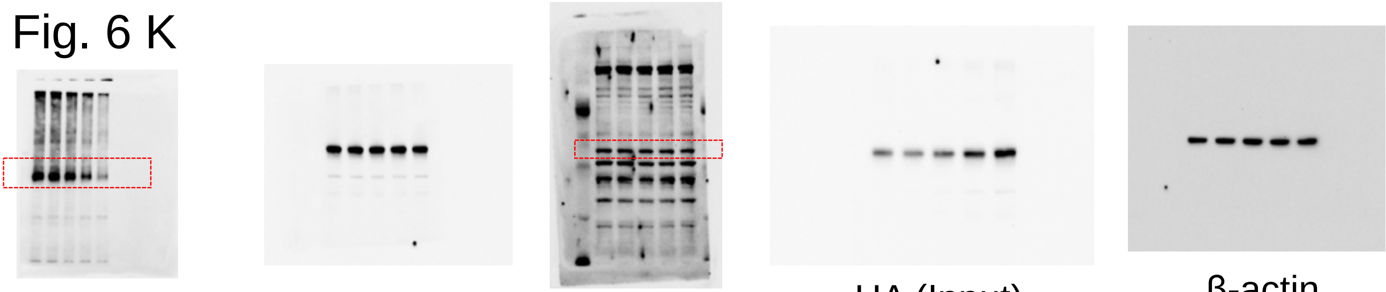

β-TrCP (PD) HA (PD) β-TrCP (Input) HA (Input) β-actin

Fig. 6 L

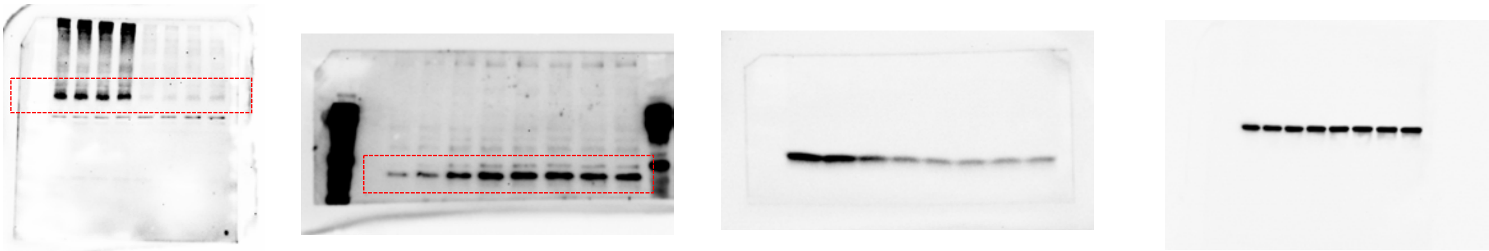

β-TrCP FBXO5 HiBiT (NSP7) β-actin

Fig. 7C

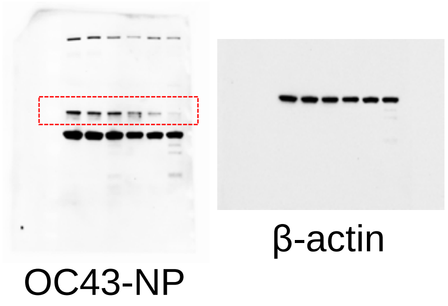

Sup Fig. 4A

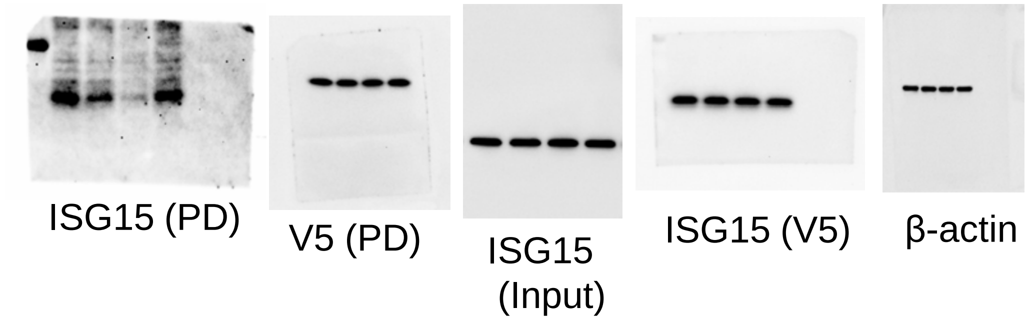

Sup Fig. 4B

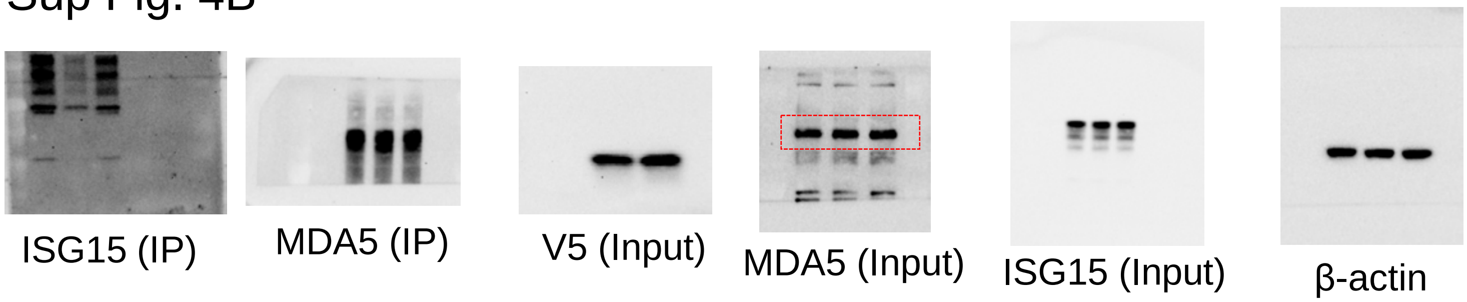

Sup Fig. 6A

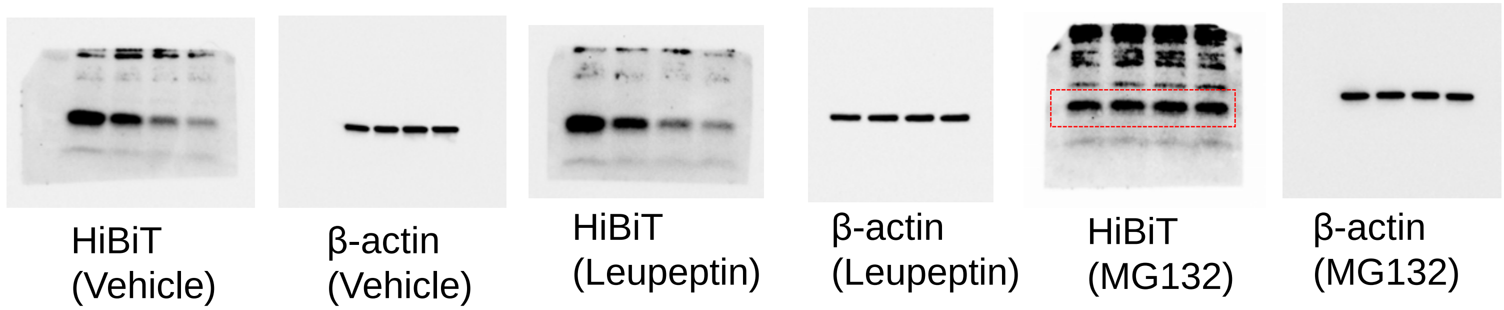

Sup Fig. 6B

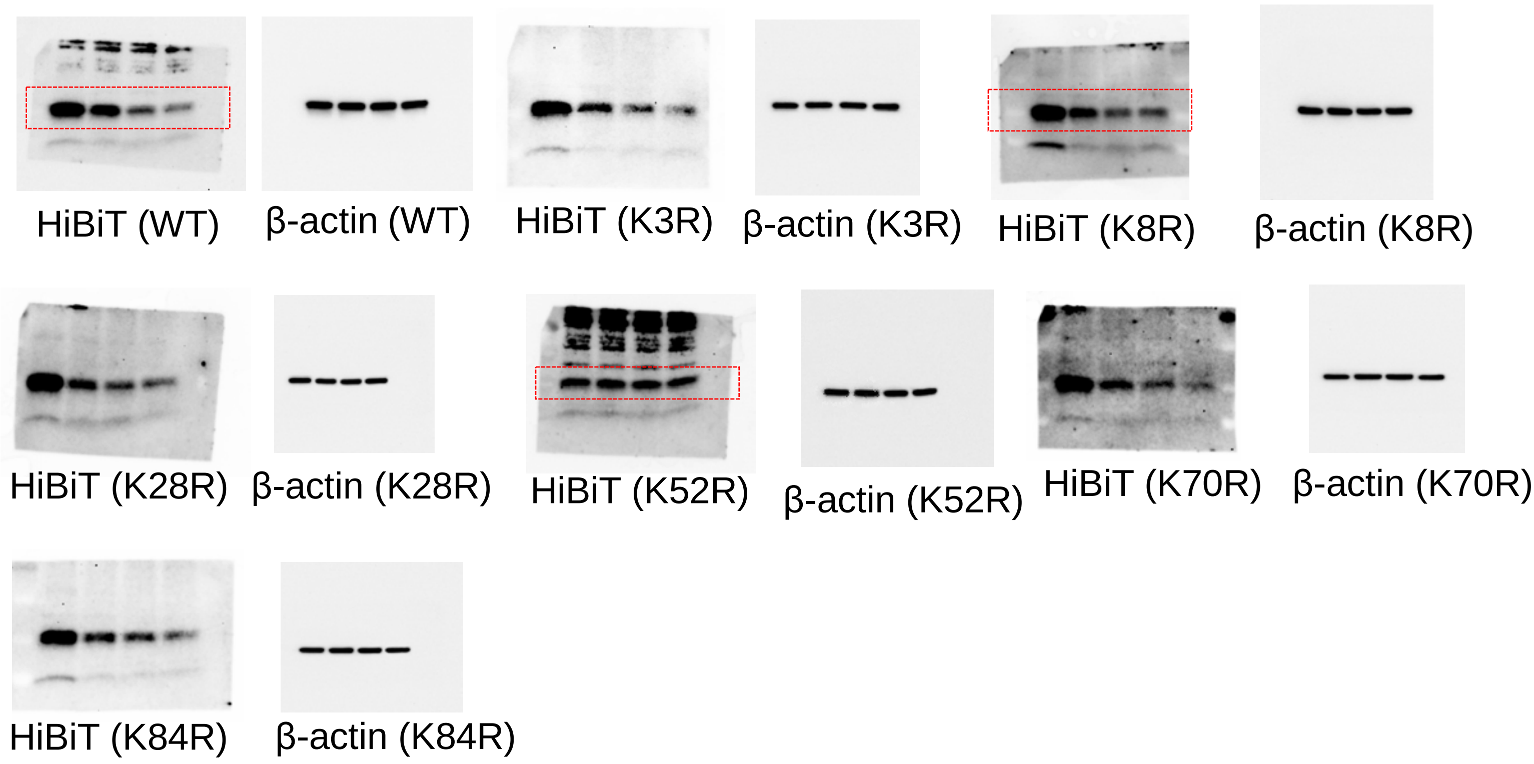

Sup Fig. 6C

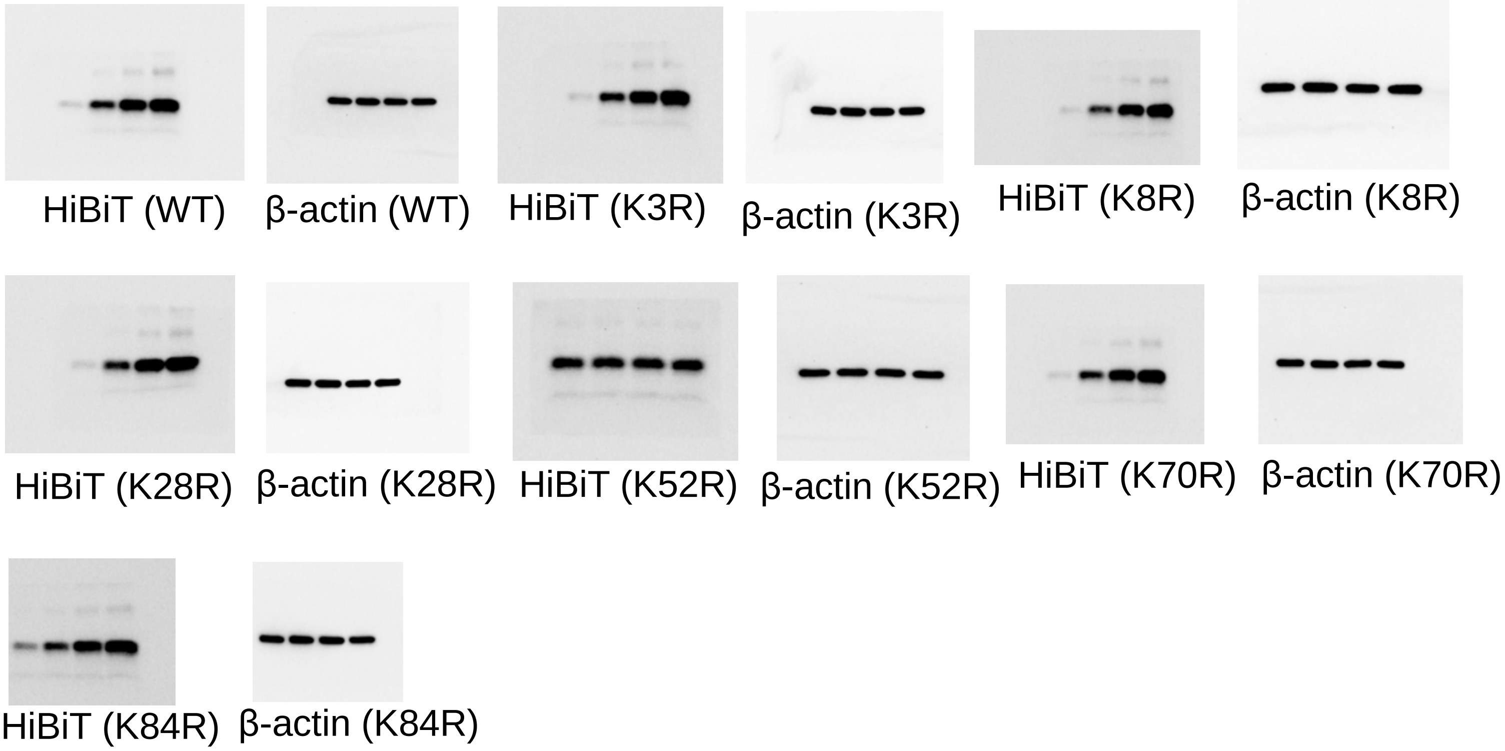

Sup Fig. 6D

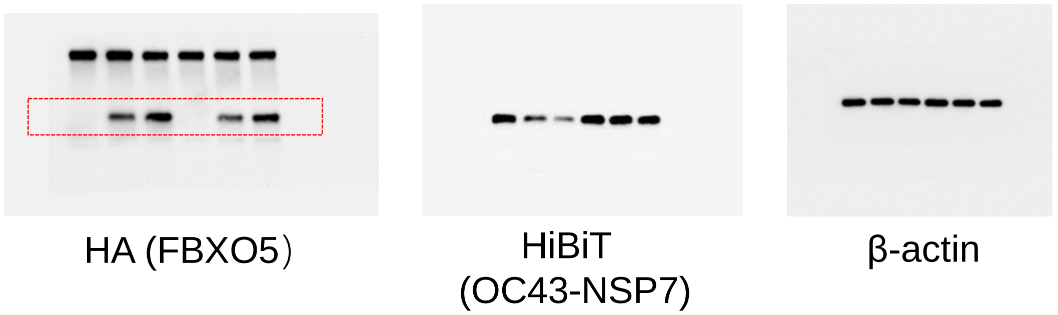

Sup Fig. 6E

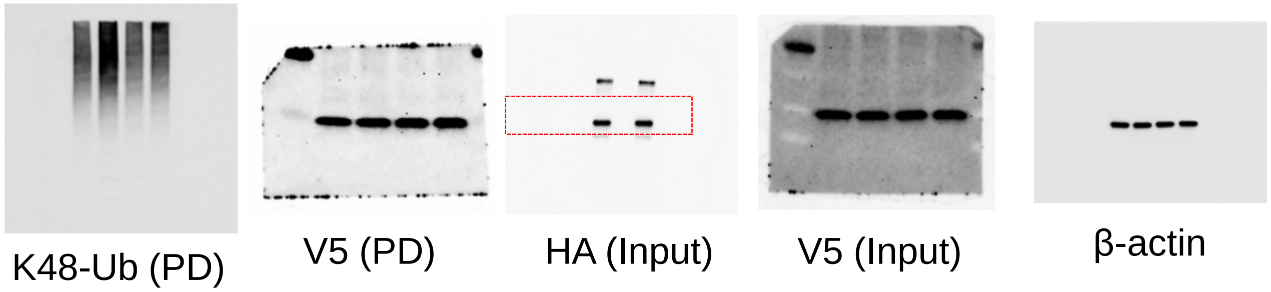

Sup Fig. 7A

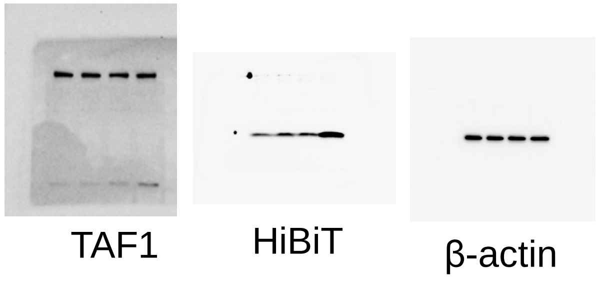

Sup Fig. 7B

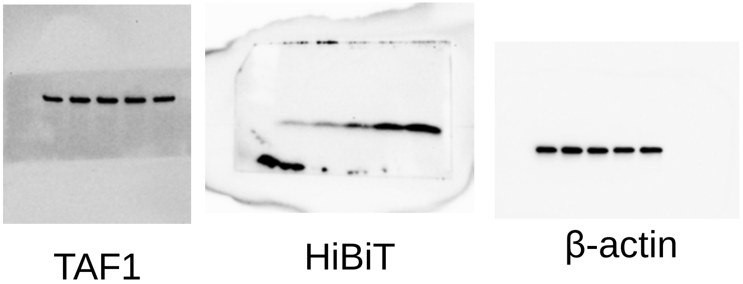

Sup Fig. 7C

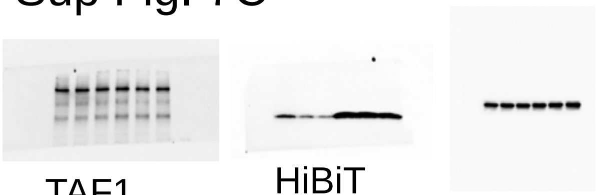

TAF1 | HiBiT | β-actin

Sup Fig. 7D

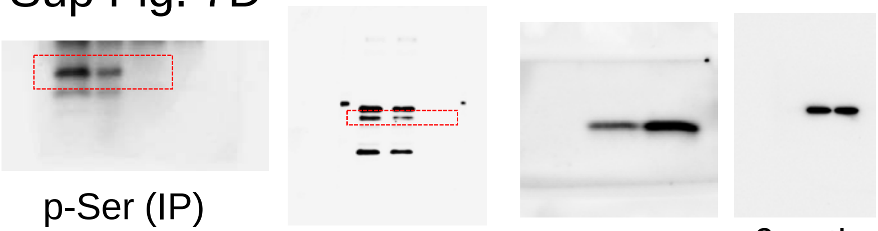

p-Ser (IP) | FBXO5 (IP) | HiBiT (Input) | β-actin

Sup Fig. 7E

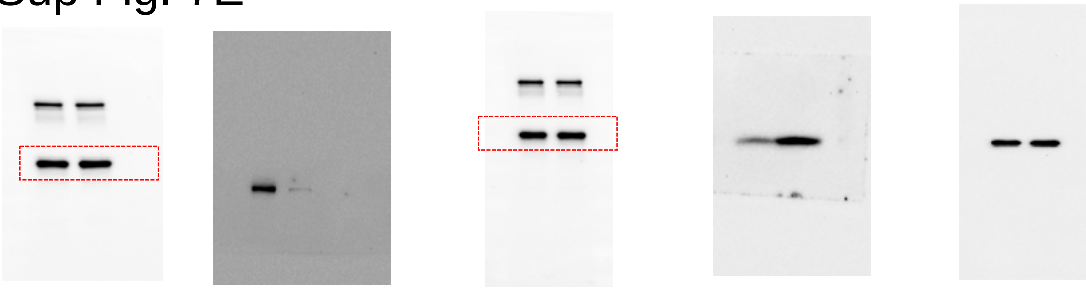

HA (PD) | HiBiT (PD) | HA (Input) | HiBiT (Input) | β-actin

Sup Fig. 7F

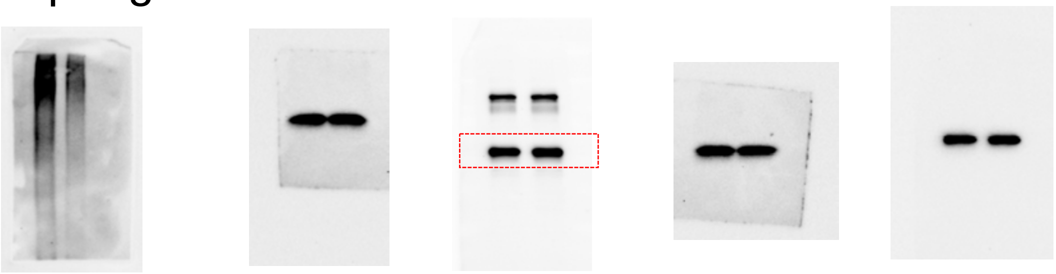

K48-Ub (PD) | V5 (PD) | HA (Input) | V5 (Input) | β-actin

Sup Fig. 8B

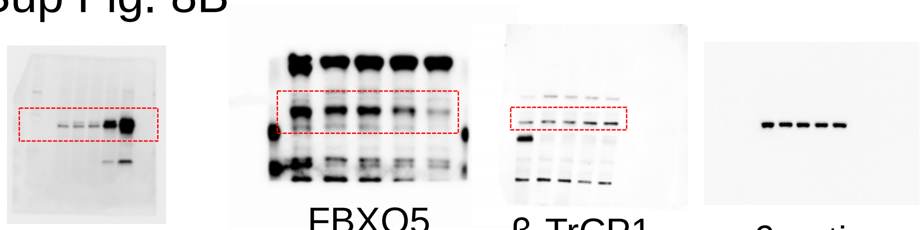

SARS-CoV-2 (NP) | FBXO5 | β-TrCP1 | β-actin

Sup Fig. 8C

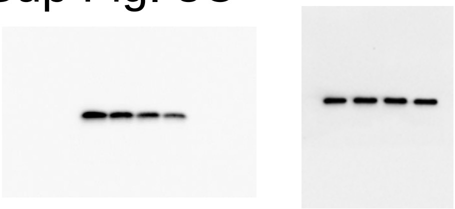

V5 (NSP7) | β-actin

Sup Fig. 8D

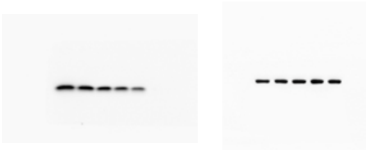

V5 (NSP7) | β-actin

Sup Fig. 8E

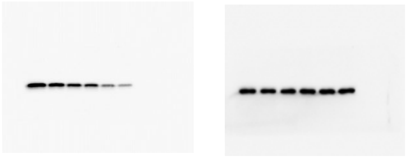

V5 (NSP7) | β-actin

Sup Fig. 8F

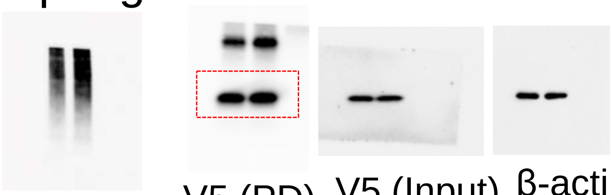

K48-Ub (PD) | V5 (PD) | V5 (Input) | β-actin

Sup Fig. 8G

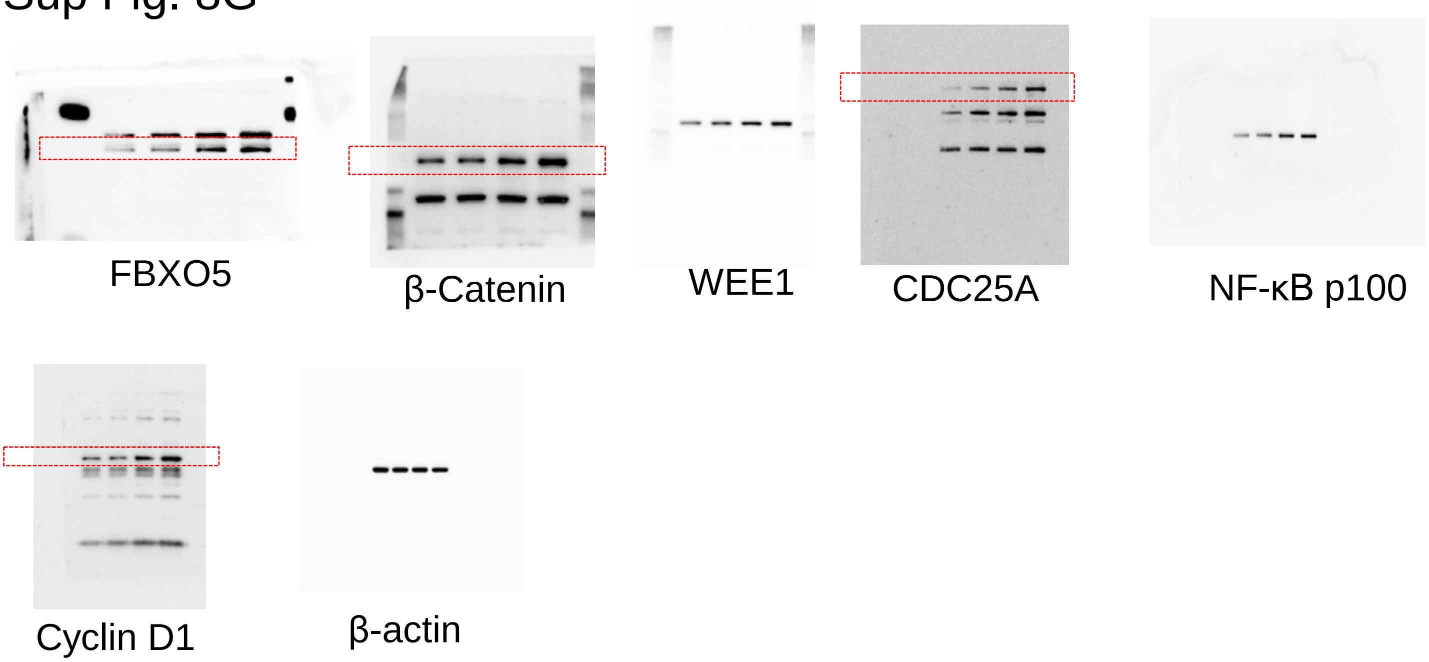

Sup Fig. 10A

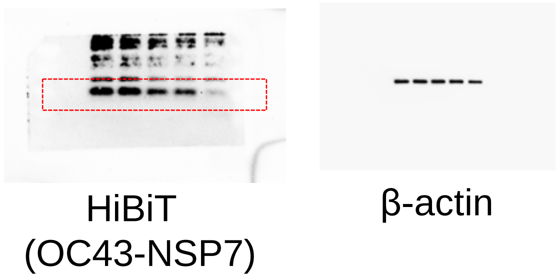

Sup Fig. 10B

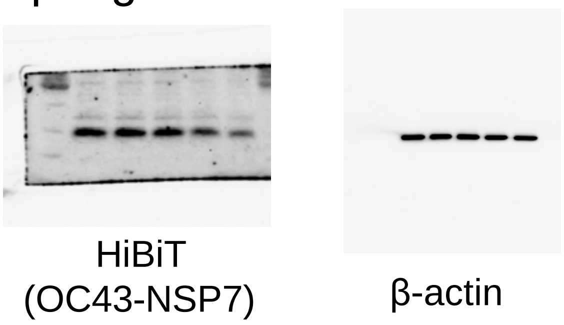

Sup Fig. 11C

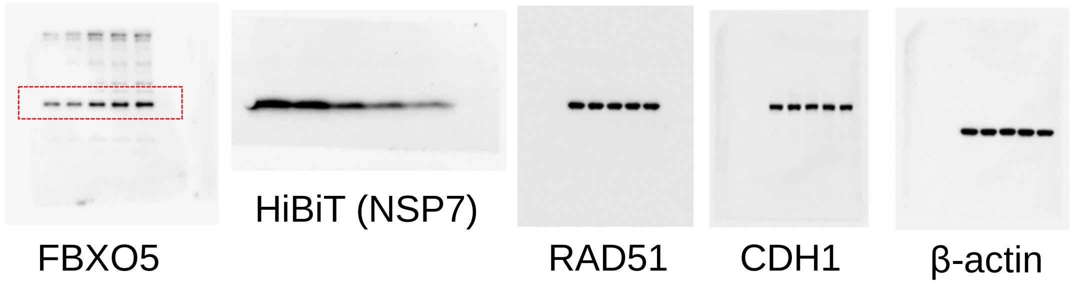

Sup Fig. 11D

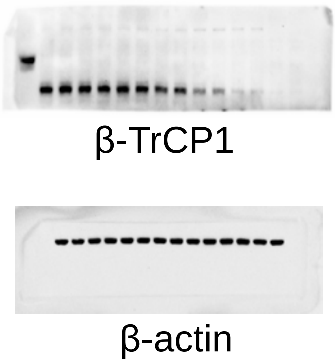

Sup Fig. 11E

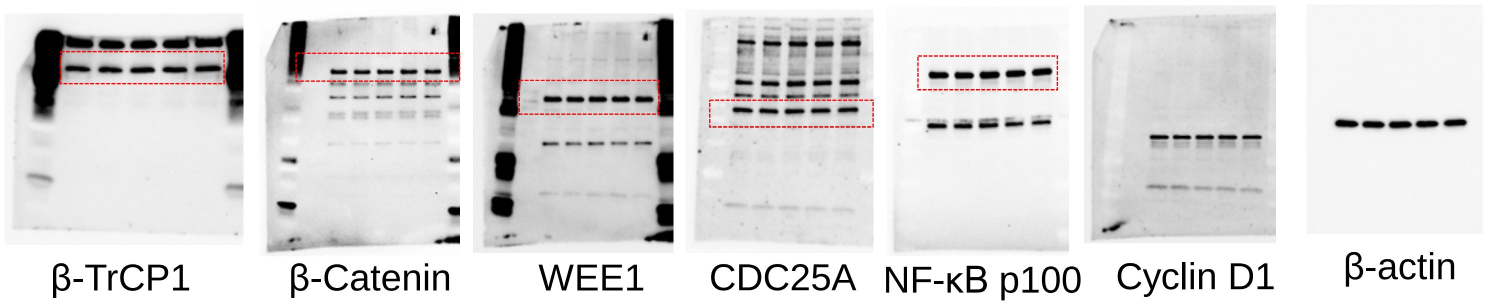

Supplement: Supplementary file 2 — Supporting Information [file ADVS-12-e00798-s002.pdf]
